# Dynamic effective connectivity in cortically embedded systems of recurrently coupled synfire chains

**DOI:** 10.1007/s10827-015-0581-5

**Published:** 2015-11-11

**Authors:** Chris Trengove, Markus Diesmann, Cees van Leeuwen

**Affiliations:** Perceptual Dynamics Laboratory, University of Leuven, Leuven, Belgium; Institute of Neuroscience and Medicine (INM-6) and Institute for Advanced Simulation (IAS-6) and JARA BRAIN Institute I, Jülich Research Centre, Jülich, Germany; Department of Psychiatry, Psychotherapy and Psychosomatics, Medical Faculty, RWTH Aachen University, Aachen, Germany; Department of Physics, Faculty 1, RWTH Aachen University, Aachen, Germany; TU Kaiserslautern, Kaiserslautern, Germany

**Keywords:** Synfire chains, Recurrent network dynamics, Background synaptic noise, Effective connectivity, Metastability, Combinatorial representation

## Abstract

As a candidate mechanism of neural representation, large numbers of synfire chains can efficiently be embedded in a balanced recurrent cortical network model. Here we study a model in which multiple synfire chains of variable strength are randomly coupled together to form a recurrent system. The system can be implemented both as a large-scale network of integrate-and-fire neurons and as a reduced model. The latter has binary-state pools as basic units but is otherwise isomorphic to the large-scale model, and provides an efficient tool for studying its behavior. Both the large-scale system and its reduced counterpart are able to sustain ongoing endogenous activity in the form of synfire waves, the proliferation of which is regulated by negative feedback caused by collateral noise. Within this equilibrium, diverse repertoires of ongoing activity are observed, including meta-stability and multiple steady states. These states arise in concert with an effective connectivity structure (ECS). The ECS admits a family of effective connectivity graphs (ECGs), parametrized by the mean global activity level. Of these graphs, the strongly connected components and their associated out-components account to a large extent for the observed steady states of the system. These results imply a notion of dynamic effective connectivity as governing neural computation with synfire chains, and related forms of cortical circuitry with complex topologies.

**Electronic supplementary material** The online version of this article (doi:10.1007/s10827-015-0581-5) contains supplementary material, which is available to authorized users.

## Introduction

Over many decades, researchers have considered various mechanisms as building blocks for neural computation: single neurons (Barlow 1972), cell assemblies (Aertsen et al. 1994; von der Malsburg 1986; Palm 1993), chains (Abeles 1982), or even cortical columns (Gray and Singer 1989). Their functional dynamics have been modeled in terms of population-averaged mean firing rates (Deco et al. (2008) and many others), collective oscillatory modes of activity (Fries et al. 2002; Gray et al. 1989; Singer 1993; Buzsaki and Andreas Draguhn 2004), or sequential generation of spikes by input synchrony: polychronous assemblies (Izhikevich 2006) or synfire chains (Abeles 1982; Diesmann et al. 1999).

For each of these systems the question arises, how computation supervenes on the dynamics; i.e. how can activity in such an architecture provide a suitable medium for parallel information processing and combinatorial representation.

Here we will address this question from the perspective of synfire chains (Abeles 1982). Synfire chains are composed of neurons that are grouped into pools; these are organized into chains by *links*. Each link consists of excitatory feed-forward connections from the neurons in one pool to those in another. The synfire chain architecture supports precisely-timed sequences of spikes; these occur in groups (called pulse packets) that activate successive pools sequentially to form a propagating wave of activity (Abeles 1982; Diesmann et al. 1999; Bienenstock 1995). Synfire wave propagation in noisy cortical environments is robust (Diesmann et al. 1999; Kumar et al. 2008a; Trengove et al. 2013b) and efficient: each neuron contributes a single spike, reliably and with precise timing, in response to a small number of coincidently arriving inputs. Consisting of only a few thousand neurons each, chains are compact meso-scale units suitable for sparse coding (Földiák 2002) and hence, in principle, for parallel processing and combinatorial representation.

Information processing using synfire chains requires structural couplings between chains. These couplings can be effectuated by links between pools of different chains (Abeles et al. 2004; Arnoldi and Brauer 1996; Schrader et al. 2010; Hanuschkin et al. 2010, 2011). Parallel coupling supports synchronized propagation on two or more chains simultaneously. This type of coupling has been used to implement feature binding in perception (Arnoldi and Brauer 1996; Abeles et al. 2004; Schrader et al. 2010). Sequential coupling allows activity to propagate on one or more chains in succession. This mechanism has been used to implement the syntax of motor sequence production (Jin 2009; Hanuschkin et al. 2010, 2011).

**Fig. 1:**
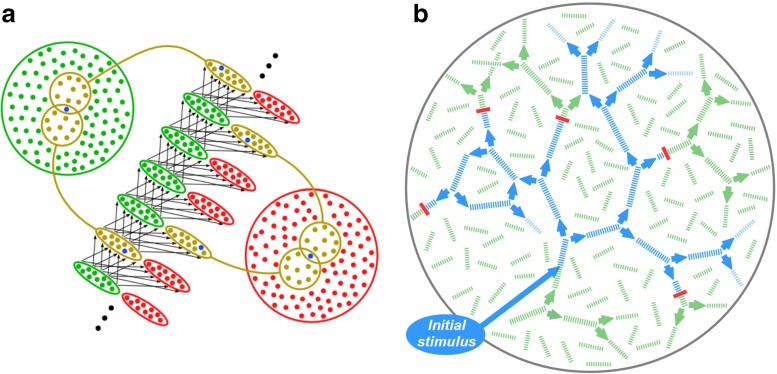
(color online) **a** Construction of synfire chain embedding in a population of neurons. A sequence of excitatory pools (*green ellipses*) is formed by randomly selecting *n*_E_ distinct neurons from the excitatory population (*large green circle*) with replacement to form each pool; two such selections are indicated in brown. A corresponding sequence of inhibitory pools (*red*) is formed from the inhibitory population (*red*). Each neuron appears in many pools; e.g. each of the neurons in blue appears in two of the pools shown. Links consist of all-to-all connections from each excitatory pool to the next excitatory pool as well as to the corresponding inhibitory pool (*arrows*). **b** Pairwise couplings (*green arrows*) between chains (*green line segments*) form a recurrent structure in which each chain has exactly two successors, chosen at random. For clarity the inhibitory pools are not shown. An initial pulse packet stimulus is the ancestor of a branching tree of ongoing pulse packet activity (*blue*), limited in size by extinctions (*red*). Loops may also be encountered

Via structural couplings, combinatorial activation has thus been realized, albeit for systems consisting of a few chains. We recently showed that synfire chain embedding on a large scale can be realized in a random network on the scale of a single cortical column (Trengove et al. 2013b). A single synfire chain comprising in the order of 10^5^ pools, each containing in the order of 100 neurons, was embedded in a network of order 10^5^ neurons (each neuron thus belonging to in the order of 100 pools; see Fig. 1a). This result implies that with a number of pools in the same order of magnitude, it is possible to embed a large number of chains in a random network.

A key problem for an embedded system of synfire chains is activity regulation. A synfire wave propagating on an embedded chain generates *background noise*: a quasi-stochastic stream of inputs that impinges on the rest of the network, potentially destabilizing it (Mehring et al. 2003). A key feature of the model of Trengove et al. (2013b) is that each individual pool contains not just excitatory but also inhibitory neurons. This results in background noise consisting of both excitatory and inhibitory inputs. Background noise is central to the dynamics of sparse recurrent random networks of excitatory and inhibitory neurons. Such networks are well described by a mean field analysis (Brunel 2000; Meffin et al. 2004; Kumar et al. 2008b). Trengove et al. (2013b) adapted this analysis to describe the dynamics of their network with synfire chains embedded in a recurrent network architecture. When background noise is suitably balanced, the network is globally stable in the presence of synfire waves, with all neurons exhibiting a low rate of irregular spiking activity, in which each spike either belongs to a wave or originates from membrane potential fluctuations driven by background noise. Background noise then has a destabilizing effect on wave propagation, increasing the probability of propagation failures. Since noise increases with wave activity, it acts as a negative feedback signal regulating the number of simultaneously propagating waves. The high chain-embedding capacity of the model, along with dynamics that support the regulated expression of wave activity, suggests a way to realize a large system of coupled chains that could support a vast repertoire of composite representations.

Using the model of Trengove et al. (2013b) as a starting point, here we introduce a large system of coupled synfire chains and examine its dynamical organisation. Rather than focusing on a function-specific coupling architecture, we will define a generic class of random recurrently coupled systems of many chains, and consider basic questions about their dynamics: how endogenously generated wave activity is sustained and regulated, how the activity modulates the structure of the system, and how the modulated structure in turn steers wave activity into stable or metastable configurations. The answers to these questions are centred on the concept of *dynamic effective connectivity*; a concept which will, we expect, offer crucial insights into how a large, recurrent synfire chain architecture may be configured to achieve information processing through ongoing synfire wave activity.

### **Model overview and effective connectivity analysis**

The current model groups the ∼10^5^ pools of Trengove et al. (2013b) into approximately 10^3^ sequentially coupled chains. The chains vary in strength according to the strength of the links between their pools. Strength variations may be considered a result of activity-dependent plasticity mechanisms such as Hebbian spike-timing dependent plasticity (STDP). The strength of a chain contributes positively to the probability it will be successfully traversed by a wave. Upon successful traversal, sequential couplings initiate wave propagation on successor chains. Each chain has two successors, i.e. the couplings form a directed random graph in which the chains are the nodes and all the nodes have an out-degree of two. This branching permits proliferation of waves which, however, is balanced by extinctions through noise feedback. In the ongoing activity that results, the number of waves fluctuates around a mean level, as does the concomitant noise feedback. The waves more likely to survive in the face of noise feedback are those that travel on stronger chains.

To explain the emergent configurations of ongoing activity, we determine the effective connectivity of each chain. This is the probability that a wave having been initiated on a chain will successfully traverse it, given the current noise environment. This probability depends on a chain’s structure – its strength and length - as well as on the fluctuating level of the noise. The latter depends, in turn, on the fluctuating number of waves; hence the effective connectivity is dynamic. Structural variability across chains induces a *topography* on the system. We quantify this topography for each chain as the maximum mean activity level for which wave traversal is deemed reliable; that is, for which the probability of traversal is above a certain threshold value. The topography then defines a nested family of effective connectivity graphs (ECGs). The ECG at a given activity level is derived from the underlying coupling graph by removing all chains which are deemed unreliable at that activity level. We use this ECG family to explain the way in which activity is distributed over the chains, relating the observed activity patterns to the strongly connected components and their associated out-components within the ECGs. That is to say, we identify certain peak regions in the topography as ‘islands of circulation’ and measure the extent to which they account for the observed patterns of activity.

Our findings demonstrate the critical importance of background noise - a generic feature of cortical networks - in modulating the effective meso-scale topology of the network. We expect that in any network containing meso-scale paths of propagation based on input synchrony, background noise will have a critical role in determining the effective connectivity and functioning of the circuitry.

To thoroughly investigate the range of activity patterns exhibited by this system we found it helpful to use a much simpler, mesoscopic model. This *reduced model* (RM) is quantitatively derived from the full model (FM) via a mean field analysis. The analysis quantifies how the probability that a wave will fully traverse a chain depends on the strength of the chain, its length, and the number of co-active waves.

Whereas the basic units of the FM are model neurons and synapses, in the RM the basic structural units are pools and links. The state is the set of active pools, updated by probabilistic propagation of activity from active pools to their successors. There is a unique RM associated with all FM instances with the same mesoscopic structure; that is, with the same couplings, chain lengths, and chain strengths. The RM can be considered as a theory for the behavior of the FM. We validate the theory by comparing the activity patterns generated by instances of both with the same mesoscopic structure.

We use the RM to efficiently characterize how system behavior varies across model instances with differing random structural parameters and degrees of strength variability. We characterize the behavior of each model instance on a run-by-run basis by key features such as the duration of ongoing activity, the mean number of waves, and, given a vector of wave activity over chains, how uniformly this activity is distributed. For each model (FM or RM), a collection of wave activity vectors is obtained over runs. The variance of the RM collection gauges the variety of RM behavior exhibited, while subjecting the RM collection to principal components analysis (PCA) and plotting the first two principal components (PCs) of the activity vectors offers a visual depiction of this variety, for both the RM and the FM. We use this depiction, in conjunction with plots of the time course of activity of particular runs, in order to identify steady states and transitions typifying each model instance.

## Methods

### The full model

This present model extends the model of Trengove et al. (2013b) by introducing (a) heterogeneity in both the strengths and lengths of chains, and (b) a system of recurrent inter-chain couplings (Fig. 1b). Values for the model parameters are given in Table 1.

**Table 1:** Model parameters

Parameters for both full and reduced models:			
*N*	number of chains	1020	–
*p*	number of pools	51020	–
*L*_mean_	mean chain length (*p*/*N*)	50.02	–
***L***	vector of *N* chain lengths	40–60	–
*G*_*μ*_	mean chain strength	0.005	–
*G*_*σ*_	chain strength variability	0–0.002	–
***G***	vector of *N* chain strengths	∼N(*G*_*μ*_, *G*_*σ*_)	–
Suc	$[N]\rightarrow [N]\times [N]$ chain coupling map	random	–
***S***	*N*×*N* adjacency matrix of chain couplings	$S_{yx}=\mathbb {1}_{y\in \text {Suc}(x)}$	–
Parameters for full model only:			
*N*_E_	number of excitatory neurons	80000	–
*N*_I_	number of inhibitory neurons	20000	–
*C*_E_	mean number of excitatory afferents per neuron	8000	–
*C*_I_	mean number of inhibitory afferents per neuron	2000	–
*n*_E_	size of excitatory pools	112	–
*n*_I_	size of inhibitory pools	28	–
*V*_E_	excitatory reversal potential	0	mV
*V*_I_	inhibitory reversal potential	−80	mV
*V*_P_	resting potential	−70	mV
*V*_R_	reset potential	−70	mV
*V*_Θ_	spike threshold	−55	mV
*τ*_P_	passive membrane time constant	20	ms
*τ*_ref_	refractory period	2	ms
*g*_I_	inhibitory synaptic conductance	0.11	–
$\tau _{\min }$	minimum transmission delay	0.5	ms
*τ*_A_	link delay spread	4.0	ms
*τ*_B_	intra-link delay spread	0.5	ms

The model uses conductance-based integrate and fire neurons with instantaneous synaptic conductance responses (Meffin et al. 2004). The evolution of the sub-threshold membrane potential of a neuron obeys the following equation:

1 $$\begin{array}{@{}rcl@{}} \frac{\mathrm{d} V}{\mathrm{d} t}&= & \frac{V_{\mathrm{P}} - V}{\tau_{\mathrm{P}}} + (V_{\mathrm{E}} - V) \sum\limits_{j,k} g_{\mathrm{E},j} \delta\left( t-t_{\mathrm{E},j}^{k}\right)\\ && + (V_{\mathrm{I}}-V)\sum\limits_{j,k}g_{\mathrm{I}}\delta\left( t-t_{\mathrm{I},j}^{k}\right) \end{array} $$

where $t_{\mathrm {E},j}^{k}$ ($t_{\mathrm {I},j}^{k}$) is the *k*th spike arrival time on the *j*th excitatory (inhibitory) synapse and *g*_E, *j*_ (*g*_I_) is the amplitude of the delta-function conductance response of the synapse.^1^ When *V*(*t*) = *V*_Θ_ the neuron fires and the potential is set to *V*_R_ for the refractory period, *τ*_ref_.

The current network structure is based on that of Trengove et al. (2013b), in which a large number *p* of excitatory pools were formed, each comprising *n*_E_ distinct neurons drawn randomly from the excitatory population of *N*_E_ neurons, so each neuron appeared in *pn*_E_/*N*_E_ pools on average. An equal number of inhibitory pools was formed in the same way from the inhibitory population and paired with the excitatory pools in a one-to-one manner. The excitatory and inhibitory pool sizes were related by *n*_E_/*n*_I_ = *N*_E_/*N*_I_ = 4. A single cyclic synfire chain was then created by arranging the excitatory pools in a cyclic sequence and for each pool *A* in the sequence creating a link (*A*, *A*^′^) comprising all-to-all synaptic connectivity from *A* to *A*^′^, the next pool in the sequence. For each link (*A*, *A*^′^) in the chain, a link (*A*, *B*^′^) was created from pool *A* to *B*^′^, the inhibitory pool paired with *A*^′^ (Fig. 1a). This results in an average of *C*_E_ afferent excitatory connections per neuron, with $C_{\mathrm {E}} = p n_{\mathrm {E}}^{2}/N_{\mathrm {E}}$. We set *N*_E_ = 10*C*_E_ so that the network has 10 % of full connectivity. In addition, each neuron receives afferent connections from neurons drawn at random from the inhibitory population, sufficient to make the ratio of excitatory to inhibitory afferent connections equal to 4. Hence *C*_E_/*C*_I_ = 4.

Transmission delays for synaptic connections were variable, being the sum of two components distributed uniformly over [*τ*_min_, *τ*_min_ + *τ*_A_] and [0, *τ*_B_] respectively. The first component, referred to as the link delay, was constrained to be identical for all synapses within a link so as not to disrupt the input synchrony of propagating waves. This constraint does not apply to inhibitory delays. The second component was drawn independently for every synapse, and provided intra-link delay variability.

For the present model the following modifications are made to this network. The single cyclic chain of *p* = 51,020 pools specified in Trengove et al. (2013b) is broken up by deleting *N* = 1020 links to obtain *N* chains of varying lengths with a mean of *L*_mean_ = *p*/*N* = 50.02. These lengths follow a centrally peaked ‘triangular’ distribution of values in the set $[40,60] \cap \mathbb {Z}$, specified by a vector ***L*** = (*L*_*x*_)_*x*∈[*N*]_ where [*N*]≡{1,…, *N*}. Chains are assigned differing *strengths*, ***G*** = (*G*_*x*_)_*x*∈[*N*]_. All the excitatory synapses within a link have the same conductance; this value is the strength of a link. Likewise, all the links within a chain *x* have the same strength; this is the strength *G*_*x*_ of the chain. Across chains, the strength follows a normal distribution of mean *G*_*μ*_ and standard deviation *G*_*σ*_ with negative values replaced by zeros. Pairwise couplings between chains are introduced, whereby each chain has exactly two successor chains randomly chosen from the *N* chains, thus forming a recurrently coupled system (Fig. 1b). The coupling from a chain to a successor consists of a link from the last pool of the one to the first pool of the other. The strength of this link equals the strength of the *successor* chain; i.e. the strength of the links making up the successor chain.^2^ The full set of couplings between chains is described by a chain successor map Suc:[*N*]→[*N*]×[*N*]. Suc(*x*) gives the two successor chains of each chain *x*∈[*N*]. The couplings form a graph *G*_0_ = (*V*_0_, *E*_0_) with chains as vertices (*V*_0_≡[*N*]) and couplings as edges. The set of edges is given by *E*_0_ = {(*x*, *y*):*S*_*yx*_ = 1} where ***S*** = (*S*_*yx*_)_*x*, *y*∈[*N*]_ is the adjacency matrix of *G*_0_, given by *S*_*yx*_ = 1 if *y*∈Suc(*x*) else 0. Equivalently Suc(*x*) = {*y*∈[*N*]:*S*_*yx*_ = 1}.

### The reduced model

The reduced model (RM) describes the network of coupled chains at the meso-scale, where the pools and links between them are the basic units. The reduced model is updated in discrete time, each time step Δ*t* being the time taken for a wave to propagate from one pool to a successor; unlike in the FM, this time interval is therefore unchanging and identical for all links. At each time step a pool is in one of two possible states: carrying a pulse packet or not. The state of the system at time *t* is the state of all the pools collectively and is specified by *W*(*t*), the set of pools at which pulse packets (waves) are present. The structure of an RM instance equals the mesoscopic structure of an FM instance, as encompassed by ***L***, ***G*** and ***S***.

A wave propagates from one pool to a successor pool with a probability *P*_s_(*h*, *g*_E_), assumed to be a function of the strength of the link over which it propagates (*g*_E_) and the number of currently active waves *h*, given by *h* = |*W*(*t*)|. The *h*-dependence of the propagation probability encapsulates the negative feedback effect of background noise whereby wave activity is regulated.

The RM update rule is as follows. For each pool *w*∈*W*(*t*), let *x*(*w*) denote the chain to which it belongs and *μ*(*w*)∈{1,…, *L*_*x*(*w*)_} its position on that chain. For 1 ≤ *μ*(*w*)<*L*_*x*(*w*)_, activity propagates probabilistically to the next pool on the same chain with probability *P*_s_(*h*, *G*_*x*(*w*)_). For *μ*(*w*) = *L*_*x*(*w*)_, the wave is on the last pool of the chain and propagates to the first pool of each successor chain *y*∈Suc(*x*(*w*)) with probability *P*_s_(*h*, *G*_*y*_). Let *V*_*w*_(*t*+Δ*t*) be the pools to which the wave at *w* propagates. Then *W*(*t*+Δ*t*) = ∪_*w*∈*W*(*t*)_*V*_*w*_(*t*+Δ*t*).^3^

The function *P*_s_(*h*, *g*_E_) for the probability of propagation over single link is related to *P*_s_(*h*, *g*_E_, *L*), the probability of propagation over a chain of length *L* and strength *g*_E_ when *h* waves are present:

2 $$ P_{\mathrm{s}}(h, g_{\mathrm{E}}) \equiv P_{\mathrm{s}}(h,g_{\mathrm{E}}, 1) = P_{\mathrm{s}}(h,g_{\mathrm{E}}, L)^{1/L} ~ .  $$

We obtain the form of *P*_s_(*h*, *g*_E_, *L*) at *L* = *L*_0_ = 50≈*L*_mean_ from a function that models propagation probability in the full model: $\hat {P}_{\mathrm {s}}(\lambda _{\mathrm {E}}, g_{\mathrm {E}}, L=L_{0})$.^4^ This function gives the probability that a wave will successfully traverse a chain of length *L* given background noise in the form of excitatory and inhibitory Poisson processes with rates *λ*_E_ and *λ*_I_ = *λ*_E_/4 and conductances *G*_*μ*_ and *g*_I_ respectively.^5^

**Fig. 2:**
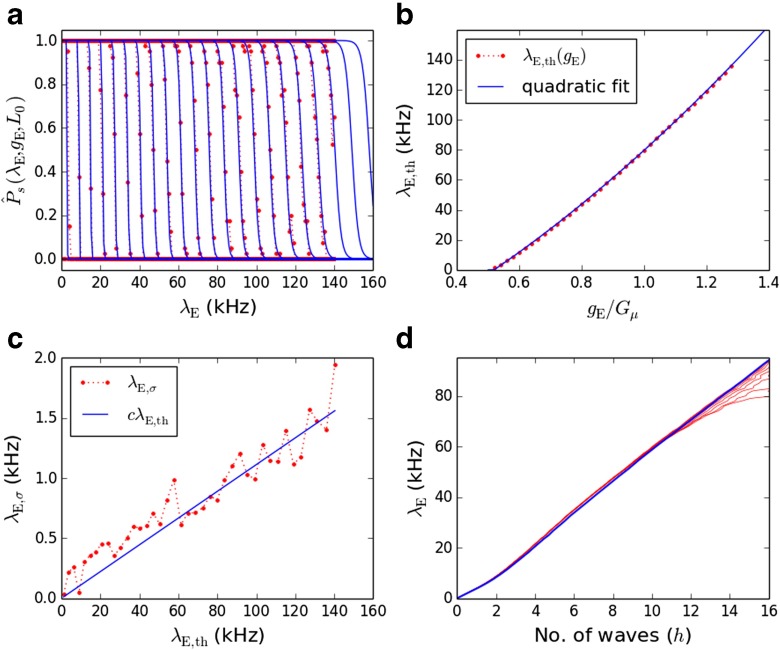
(color online) **a** Wave propagation probability $\hat {P}_{\mathrm {s}}(\lambda _{\mathrm {E}},g_{\mathrm {E}},L_{0})$ estimated from simulations (*red*) and from the model given by Eq. (3) (*blue*) for *g*_E_/*G*_*μ*_ ∈ {0.5,0.54,…,1.4}. **b** *g*_E_-dependence of *λ*_E,th_, the threshold for 50 % wave propagation probability from sigmoidal fits to $\hat {P}_{\mathrm {s}}(\lambda _{\mathrm {E}},g_{\mathrm {E}},L_{0})$, for individual *g*_E_ values (*red*) and fitted to a quadratic (*blue*). **c** The relationship between the sigmoid widths *λ*_E, *σ*_ and the thresholds *λ*_E,th_ is approximated by a linear relationship *λ*_E,σ_ = *c* *λ*_E,th_. **d** The *h*- *λ*_E_ relationships based on the expected contribution to the population mean firing rate made by each each wave, *ν*_W,1_(*λ*_E_, *G*_*σ*_) (*red lines*); and setting *ν*_W,1_ to a constant value of *ν*_W,1_(*λ*_E,th_(*G*_*μ*_),0.0015) (*blue line*)

We numerically estimate $\hat {P}_{\mathrm {s}}(\lambda _{\mathrm {E}}, g_{\mathrm {E}}, L=L_{0})$ by simulations of wave propagation on individual chains of length *L*_0_, for ordered pairs (*g*_E_, *λ*_E_) on a rectangular grid, with *n* = 40 trials for each case. The data estimating $\hat {P}_{\mathrm {s}}$ for each *g*_E_ value are well-fitted by a sigmoidal function of the form *σ*((*λ*_E,th_−*λ*_E_)/*λ*_E,σ_) where *σ*(*z*) = 1/(1 + *e*^−*z*^) (Fig. 2a). The threshold noise levels for 50 % propagation probability, *λ*_E,th_(*g*_E_), increase smoothly from zero as *g*_E_ increases from *g*_E,0_ = 0.00259 and are well-fitted by a quadratic threshold function *λ*_E,th_(*g*_E_) (Fig. 2b). Over most of the *g*_E_ range the width of the sigmoid, *λ*_E, *σ*_(*g*_E_), is approximately proportional to the threshold (Fig. 2c). Therefore we assumed strict proportionality, replacing *λ*_E,σ_(*g*_E_) with *cλ*_E,th_ where *c* = 0.01112 is the average of *λ*_E, *σ*_(*g*_E_)/*λ*_E,th_(*g*_E_) over a suitable *g*_E_ range (0.0045< = *g*_E_<=0.0065). Thus we obtained our $\hat {P}_{\mathrm {s}}$ model:

3 $$ \hat{P}_{\mathrm{s}}(\lambda_{\mathrm{E}}, g_{\mathrm{E}}, L_{0}) = \sigma\left( \frac{\lambda_{\text{E,th}}(g_{\mathrm{E}}) - \lambda_{\mathrm{E}}}{c\lambda_{\text{E,th}}(g_{\mathrm{E}})}\right) {\Theta}(g_{\mathrm{E}} - g_{\mathrm{E,0}})  $$

where Θ(⋅) is the Heaviside step function.

To translate $\hat {P}_{\mathrm {s}}(\lambda _{\mathrm {E}}, g_{\mathrm {E}}, L_{0})$, which models wave propagation in the FM, into *P*_s_(*h*, *g*_E_, *L*_0_), which models wave propagation in the RM, we need *λ*_E_ as a function of *h*, the number of synfire waves. For the model of Trengove et al. (2013b) this function *λ*_E_(*h*) is determined by

4 $$ \lambda_{\mathrm{E}} = C_{\mathrm{E}} (\nu_{\mathrm{S}}(\lambda_{\mathrm{E}}) + \nu_{\mathrm{W}}(h, \lambda_{\mathrm{E}})) \; ,  $$

where *ν*_S_(*λ*_E_) is the rate of stochastic spiking per neuron in response to background input and *ν*_W_(*h*, *λ*_E_) is the rate per neuron of spikes belonging to waves. In Trengove et al. (2013b) *ν*_W_(*h*, *λ*_E_) = *hν*_W,1_(*λ*_E_, *g*_E_) where *ν*_W,1_ is the contribution to *ν*_W_ made by each wave. In the present model, however, due to variable chain strengths, *ν*_W,1_ will generally differ for each wave in *W*(*t*), as it depends upon the strength of the specific chain on which a wave propagates. We can regain *ν*_W_ as a function of *h* by replacing *ν*_W,1_ with the *expected* contribution to *ν*_W_ made by each wave, based on a distribution for the strengths of the chains on which the current waves reside, given background rate *λ*_E_. This leads to a *λ*_E_(*h*) function that depends upon strength variability (red curves in Fig. 2d). For the RM model we further simplified *λ*_E_(*h*) by using a *G*_*σ*_-independent fixed value for *ν*_W,1_ (blue curve in Fig. 2d). For details, see Online Resource 1, Section 1.

### Numerical experiments

#### **Design**

We studied the behavior of both the FM and RM with numerical simulations. Ninety individual RM instances were generated from 9 settings of chain strength variability (*G*_*σ*_), in combination with 10 random settings of the remaining structural parameters specifying an instance: chain couplings, chain lengths and *relative* chain strengths. These are referred to as RM parameter settings (RMPs). For each RMP setting, as *G*_*σ*_ is varied the relative chain strengths ((***G***−*G*_*μ*_)/*G*_*σ*_) stay the same; only the magnitudes of the deviations from the mean change. The resulting 90 RMs are identified by the extended RMP index 10*i* + *j* where *i* is the *G*_*σ*_-index (*G*_*σ*_/*G*_*μ*_ = {0.05*i*; *i* = 0,…,8}) and *j* is the RMP index, *j*∈{0,…,9}. For each of these 90 RMs we conducted 5000 RM simulations (runs) and 10 FM simulations (runs). Besides the parameters which a given FM shares with the corresponding RM, the FM has additional random parameters: pool constituents, delays, inhibitory connections, initial pulse packet stimulus jitter and transient background noise. These random parameters varied over runs and *G*_*σ*_ settings but not over RMPs. For each FM and RM run a pool was independently and randomly selected as the target of the initial pulse packet stimulus. Each FM run had a duration of 150,000 ms and began with 200 ms of external balanced noise input, after which the external noise was turned off and the initial pulse packet stimulus was delivered. In the RM runs this was done by initializing the state of the system at *t* = 0 with one wave present at the selected target pool. The durations of the RM runs for all RMs with a given *G*_*σ*_ were matched to the corresponding FM run durations by setting Δ*t* = *T*, the mean pool-to-pool wave propagation time averaged over the 100 FM runs at that *G*_*σ*_ (see Post-processing). We thereby took into account a small but steady drift in *T* with *G*_*σ*_ in excess of the typical variations in *T* over runs with the same *G*_*σ*_.

#### **Implementation**

The FM simulations were programmed in Python and implemented in NEST (Gewaltig and Diesmann 2007) via the PyNest interface (Eppler et al. 2009). Each simulation used a time-step of 0.1 ms. Simply as an efficient device for detecting and recording all pulse packets, readout neurons, one for each pool, were included. Each readout neuron is an integrate and fire neuron with *n*_E_ afferent current-based synapses, one from each excitatory neuron in its associated pool, that deliver instantaneous voltage increments of 1 mV when activated. As shown in Table 2, the firing threshold is *n*_E_/2 mV above the resting potential. The neuron has a short time constant chosen so that it responds very reliably to pulse packets of narrow dispersion (below 0.5 ms) such as occur in the system, and virtually never to background activity given the firing rates occurring in the network, which are below 50 Hz. Very occasionally a pulse packet is missed, to negligible impact. Exploiting the parallel processing capability of NEST, each simulation required up to 96 GB of memory, used 48 processors in a cluster of 960 processors and took about 3 h to complete. With about 8–12 simulations running in parallel, the total of 900 simulations took approximately 270 h to complete. The RM model was implemented in Python and took approximately 31 h to complete using trivial parallelization to distribute the 450,000 simulations over 20 cores of a multi-core machine.

**Table 2:** Parameters of pulse-packet detector neurons

*V*_rest_	resting potential	0	mV
*V*_reset_	reset potential	0	mV
*V*_Θ_	spike threshold (*n*_E_/2)	56	mV
*τ*_m_	membrane time constant	2.5	ms
*τ*_ref_	refractory period	2	ms

#### **Post-processing**

For each FM run, the spike trains of 5 % of the excitatory neurons were recorded and used to gauge the mean firing rate of the network in 10 ms time bins.

Very infrequently the FM model exhibits an explosive instability in which the network goes into a state in which all neurons fire at high rate limited only by the refractory period. This state is an artifact of the 0.1 ms simulation time step and the instantaneous post-synaptic conductance responses. The instability was encountered at the two highest choices of chain strength variability: in 1 of 100 runs at *G*_*σ*_/*G*_*μ*_ = 0.35 and in 6 of 100 runs at *G*_*σ*_/*G*_*μ*_ = 0.4. These runs were omitted from further analysis.

For each FM and RM run we calculated *T*, the mean pool-to-pool propagation time, and $\bar {h}$, the average number of simultaneously propagating waves during the interval in which ongoing activity was sustained, as follows. We let $\mathcal {P} = \{\pi _{k}\equiv (t_{k}, p_{k}), k = 1, \ldots , n_{\text {pp}}\}$ denote the set of detected pulse packets, where *t*_*k*_, *p*_*k*_ are the time and pool at which the *k*th pulse packet occurred and *n*_pp_ is the number of pulse packets. In the FM case, we identify wave propagation between two successive pulse packets (*t*_*i*_, *p*_*i*_), (*t*_*j*_, *p*_*j*_) if there exists a link (*p*_*i*_, *p*_*j*_) and the time interval *t*_*j*_−*t*_*i*_ is within a tolerance interval around the expected time for a wave to propagate over the link given the link delay; i.e. if $t_{j} - t_{i} - \tau _{p_{i} p_{j}} - t_{\text {rise}} \in [-t_{\epsilon }, t_{\epsilon }]$ where *t*_*𝜖*_ = 0.5 ms, $\tau _{p_{i} p_{j}}$ is the link delay and *t*_rise_ = 0.23 ms is an estimate of the mean lag from the mean arrival time of a pulse packet’s inputs to a neuron to the time of the resulting spike. Let $\mathcal {L} \subset \mathcal {P} \times \mathcal {P}$ denote the set of all pulse packet pairs over which wave propagation has been identified. Then the mean number of waves during the time interval [*t*_*a*_, *t*_*b*_], where *t*_*a*_ (*t*_*b*_) is the time of the first (last) pulse packet in $\mathcal {P}$, is given by $\bar {h} = T_{\text {sum}} / (t_{b} - t_{a})$ where $T_{\text {sum}} = {\sum }_{\{i,j | (\pi _{i}, \pi _{j}) \in \mathcal {L}\}} t_{j} - t_{i}$. The mean pool-to-pool propagation time *T* is given by $T = T_{\text {sum}} / \#\mathcal {L}$. In the RM case, *T* is determined a priori from the corresponding FM runs (as noted above) and $\bar {h}$ is simply the average of *#W*(*t*) over the interval of ongoing activity.

#### **Characterization of activity patterns**

For each FM or RM run we characterized the temporal patterns of pulse packet activity in the network by the set of *end events* (EEs), namely, all pulse packets which occurred on the last pool of a chain: $\mathcal {E} = \{(t, x(p))\, :\, (t,p) \in \mathcal {P}, \mu (p)=L_{x(p)}\}$, and calculated the end event count (EEC): the number of end events on each chain, as a vector, $\boldsymbol {C} \equiv (C_{k})_{k=1,\ldots ,N} = (\#\{(t,k)\in \mathcal {E}\})_{k=1,\ldots ,N}$, which we normalized by its sum to obtain a normalized EEC (NEEC):

$$\boldsymbol{D} = (D_{k})_{k=1,\ldots,N} $$

where $D_{k} = C_{k} / {\sum }_{j=1}^{N} C_{j}$.

We obtained a measure of uniformity of activity over chains by considering an NEEC as a probability distribution over chains and computing its entropy:

$$H(\boldsymbol{D}) = - \sum\limits_{j=1}^{N} D_{j} \log_{2} D_{j}. $$

Each RM and each set of mesoscopically equivalent FMs may be identified by the triplet *ρ* = (*α*, *γ*, *m*), where *α* gives the RMP index, *γ* the *G*_*σ*_ index and *m* the model type (RM or FM). For each *ρ*, we removed runs in which ongoing activity failed to persist for more than 10000 ms. This left *M*_*ρ*_ runs, with *M*_*ρ*_ ranging from 4089 to 5000 over the 90 RMs with a mean of 4796. For the 90 sets of mesoscopically equivalent FMs, *M*_*ρ*_ ranged from 0 to 10 with a mean of 9.

To characterize the behavior of each RM instance *ρ* we performed PCA on the set of NEEC vectors, $\mathcal {D}^{\rho } = \{\boldsymbol {D}^{\rho }_{j}\,;\, j=1,\ldots ,M_{\rho }\}$. These NEEC vectors constitute an *N*-by- *M*_*ρ*_ matrix *D*^*ρ*^ where $(D^{\rho }_{ij})_{i=1,\ldots ,N} = \boldsymbol {D}^{\rho }_{j}$. We obtained the eigenvectors $\boldsymbol {E}^{\rho }_{j}$ and eigenvalues ${\Lambda }^{\rho }_{j}$ (*j* = 1,…, *M*_*ρ*_) of the covariance matrix $M_{\rho }^{-1}(D^{\rho } - \bar {\boldsymbol {D}}^{\rho }\mathbf {1}^{T}_{M_{\rho }})(D^{\rho } - \bar {\boldsymbol {D}}^{\rho }\mathbf {1}^{T}_{M_{\rho }})^{T}$, where $\bar {\boldsymbol {D}}^{\rho } = M_{\rho }^{-1}{\sum }_{j=1}^{M_{\rho }} \boldsymbol {D}^{\rho }_{j}$ is the run-averaged NEEC and $\mathbf {1}_{M_{\rho }}$ is a vector of *M*_*ρ*_ ones. We plotted the first two PCs, $\{((\boldsymbol {D}^{\rho }_{j}-\bar {\boldsymbol {D}}^{\rho })\cdot \boldsymbol {E}^{\rho }_{1}, (\boldsymbol {D}^{\rho }_{j}-\bar {\boldsymbol {D}}^{\rho })\cdot \boldsymbol {E}^{\rho }_{2})|j=1,\ldots ,M_{\rho }\}$ to provide a visual representation of the range of behaviors (the 2PC projection). In cases where it was visually evident that this representation was strongly influenced by a small number of outliers, the PCA was repeated with outliers excluded to obtain another 2PC projection. We used the 2PC projections in conjunction with raster plots of end events for selected runs to elucidate the nature of the steady states of the system, their variability across runs, and the transitions between them.

### Effective connectivity analysis

We seek to understand the relationship between structure and steady states of ongoing activity in a system of coupled chains. Our hypothesis is that a steady state consists of activity on one or more *islands of circulation* upon which propagation of activity is effectively confined, due to noise feedback. To identify such islands, we introduce a method of analysis based on the *effective connectivity structure* (ECS) that is steady state dependent. An ECS for a given RM includes a set of *expected* chain traversal probabilities:

$$\{P(x|p(h)); x \in [N]\}, $$

where *p*(*h*) is the probability distribution function for the number of waves present over the duration of a steady state. To obtain these probabilities we assume that, within a steady state, *h*(*t*) does not typically vary much over *L*_*x*_ consecutive values $(h_{1}, \ldots , h_{L_{x}} )$ of *h*(*t*), where *L*_*x*_ is the length of a chain (*L*_*x*_ ∈ [40,60]). The probability that a wave will successfully traverse a chain is then given by:

5 $$\begin{array}{@{}rcl@{}} P(x|p(h)) & =& \left\langle \prod\limits_{i=1}^{L_{x}} P_{s}(h_{i}, G_{x}, 1) \right\rangle_{p(h_{1},\ldots,h_{L_{x}})}\\ && \approx \left\langle P_{s}(h, G_{x}, 1)^{L_{x}} \right\rangle_{p(h)} \end{array} $$

where $p(h_{1},\ldots ,h_{L_{x}})$ is the multivariate probability distribution for a sequence of *L*_*x*_ consecutive values of *h* within a steady state.

#### **Effective connectivity graphs**

An ECS consists of both a set of expected chain traversal probabilities {*P*(*x*|*p*(*h*))} and an underlying coupling graph *G*_0_ = (*V*_0_≡[*N*], *E*_0_). An ECS admits a family of effective connectivity graphs (ECGs) parametrized by *θ*, a pruning threshold for the traversal probability:

6 $$\begin{array}{@{}rcl@{}} G(\theta; p) & = & (V, E) \end{array} $$

7 $$\begin{array}{@{}rcl@{}} V & = & \{x\in V_{0}:P(x|p(h)) \geq \theta\} \end{array} $$

8 $$\begin{array}{@{}rcl@{}} E & = & \{(x,y) \in E_{0}: x,y \in V\} \end{array} $$

That is, we prune from *V*_0_ the sub-threshold chains {*x*:*P*(*x*|*p*(*h*))<*θ*}.

We obtain what we term the *empirical* ECS by setting *p*(*h*) = *p*_emp_(*h*), the empirical *h* distribution for ongoing activity observed over all time steps and runs of the model instance. To handle the presence of multiple steady states one would ideally segment the data according to steady states and compute a *p*(*h*) and an ECG for each steady state. For simplicity, we work with the assumption that *p*(*h*) = *p*_emp_(*h*) will be adequate to characterize the steady states which predominate.

The empirical ECS determines a *θ*-family of ECGs, *G*(*θ*;*p*_emp_). Given this family one may seek optimal values of *θ*: those for which the island structure in the ECG best describes the observed distribution of activity over chains. However, instead of using this *θ*-family we set the pruning threshold to a fixed value^6^ and introduce a parameter, $\bar {h}$, that allows us to shift the mean of the distribution *p*(*h*) away from that of *p*_emp_(*h*). More specifically, we define a family of bell-shaped distributions $p(h|\bar {h})$ with the same variance as *p*_emp_(*h*) parametrized by the mean value, $\bar {h} > 0$. This gives rise to an $\bar {h}$-dependent family of graphs $G(\bar {h}) \equiv G(\theta ; p(h|\bar {h}))$. The form of $p(h|\bar {h})$ is obtained by discretizing a normal distribution $\mathrm {N}(\bar {h}^{\prime },\sigma )$ to bins centred around 0,1,…, *h*_max_ = 40 and normalizing, $\bar {h}^{\prime }$ chosen such that the result has mean $\bar {h}$. As $\bar {h}$ increases, $P(x|p(h|\bar {h}))$ decreases for all *x*∈[*N*]. This property allows us to define a topography (or landscape) on the system of coupled chains. The ‘height’ of chain *x* is an activity threshold, $\bar {h}_{\text {th}}(x)$, defined as $\bar {h} > 0$ such that $P(x|p(h|\bar {h})) = \theta $, or zero if no such $\bar {h}$ exists. Hence $x \in V(\bar {h}) \,\text {iff} \, \bar {h} \leq \bar {h}_{\text {th}}(x)$. Hence the size $V(\bar {h})$ is non-increasing and decreases each time $\bar {h}$ rises above the activity threshold of a chain, at which point it is pruned.

We will obtain $\bar {h}$ values which optimize the relationship between the structure of $G(\bar {h})$ and the observed patterns of activity. These $\bar {h}$ values are predictions for the empirically observed mean *h* and how it varies across models.

#### **Graph structure**

For a given graph $G(\bar {h})$ in the $\bar {h}$-family we identify $\text {SC}(\bar {h})$, the set of strongly connected components – or strong components (SCs) for short – and their associated out-components (OCs). An SC is a maximal subset of $V(\bar {h})$ with the property that there are directed paths in both directions from every vertex in the SC to every other vertex in the SC (Newman 2010). Note that by this definition an SC is either *cyclic* (i.e contains a cyclic path) or is a single node without a self-connection. The latter we will ignore because we are only interested in SCs which support ongoing circulation of pulse packet activity. The OC of an SC, OC(SC), is the set of vertices reachable from any node in the SC and includes the SC itself. We let $\text {SC}(\bar {h})$ denote the set of SCs in $G(\bar {h})$, $\text {OC}(\bar {h}) = \{\text {OC}(\text {SC})|\text {SC}\in \text {SC}(\bar {h})\}$ the corresponding out-components, $\text {USC}(\bar {h})$ the union of the SCs in $\text {SC}(\bar {h})$ and $\text {UOC}(\bar {h})$ the union of the OCs in $\text {OC}(\bar {h})$. SCs are always non-overlapping but OCs may overlap: the OC of one SC (SC_1_) may contain part of the OC of another (SC_2_); this part either excludes SC_2_ entirely or it includes SC_2_ and therefore OC_2_ entirely.

Because $V(\bar {h}_{2}) \subset V(\bar {h}_{1})$ if $\bar {h}_{2} > \bar {h}_{1}$, $G(\bar {h}_{2})$ is a sub-graph of $G(\bar {h}_{1})$. It follows that every SC in $G(\bar {h}_{2})$ is a subset of an SC in $G(\bar {h}_{1})$, and likewise for the corresponding out-components. Likewise, $\text {UOC}(\bar {h}_{2}) \subset \text {UOC}(\bar {h}_{1})$ if $\bar {h}_{2} > \bar {h}_{1}$. Thus for a monotonic increasing sequence of $\bar {h}$-values the sets $\text {UOC}(\bar {h})$ form a sequence of nested subsets. We may liken them to nested regions in a landscape that circumscribe peaks by sectioning them at different altitudes. Note that while any chain with a high activity threshold can be considered as a peak in the landscape, only peaks belonging to the $\text {UOC}(\bar {h})$ family are accessible to ongoing activity which, due to its recurrent nature, can act as a regenerative source.

On the basis of the nested structure of $\text {UOC}(\bar {h})$, we assign to each chain a measure of the maximum activity level that still permits a chain to participate in ongoing activity:

9 $$ \bar{h}_{\text{circ}}(x) = \max\{\bar{h} : x \in \text{UOC}(\bar{h}) \}  $$

The function $\bar {h}_{\text {circ}}(x)$ provides a ranking of the chains which can be compared with one based on their observed EE activity, $\bar {\boldsymbol {D}}^{\rho }$. A rank correlation of the two, $\text {RC}(\bar {\boldsymbol {D}}^{\rho }, \bar {h}_{\text {circ}}^{\rho })$, provides a scalar measure of the extent to which the $\bar {h}$-family of ECGs explains the overall distribution of wave activity for model instance *ρ*.

When a system finds its equilibrium at a mean level $\bar {h}$, we predict that $\text {UOC}(\bar {h})$ will best account for the locus of wave activity. Conversely, if a particular $\text {UOC}(\bar {h})$ best accounts for the observed wave activity, then $\bar {h}$ is the *predicted* equilibrium level of activity. The predicted value for $\bar {h}$ is determined by considering the trade-off between capturing as much of the EE activity as possible and keeping the size of $\text {UOC}(\bar {h})$ as small as possible. We define the normalized size of the UOC: $\text {Size}(\bar {h}) = |\text {UOC}(\bar {h})| /|\text {UOC}_{0}|$ where UOC_0_ is the union of OCs of SCs in *G*_0_; and we let $\text {Frac}(\bar {h}) = {\sum }_{x \in \text {UOC}(\bar {h})} (\bar {\boldsymbol {D}}^{\rho })_{x}$, the fraction of EE activity accounted for by chains in $\text {UOC}(\bar {h})$. We consider two methods for optimizing $\bar {h}$: in method 1 the value which maximizes the function $\text {Frac}(\bar {h}) (1 - \text {Size}(\bar {h}))$; in method 2 the value where $\text {Frac}(\bar {h})=1-\text {Size}(\bar {h})$. We will use both methods for comparing the optimal $\bar {h}$ with that found in the simulations.

We visualize the SC and OC structure of the graph $G(\bar {h})$ in order to shed light on how the effective connectivity structure is responsible for the observed patterns of activity. For a given graph $G(\bar {h})$ we create a special condensed graph $\text {CG}(\bar {h})$ based on a partition of the vertices of $G(\bar {h})$ into subsets of nodes that share the same profile of membership across the SCs and OCs. Writing $\text {SC}(\bar {h}) = \{\text {SC}_{1},\ldots ,\text {SC}_{n}\}$ and $\text {OC}(\bar {h}) = \{\text {OC}_{1},\ldots ,\text {OC}_{n}\}$, we partition [*N*] into the following subsets: (a) each of the SCs themselves; and (b) the sets which partition $\text {UOC}(\bar {h}) - \text {USC}(\bar {h})$ according to which OCs each chain belongs to, ie. all non-empty sets *S*_*π*_, *π*∈{0,1}^*n*^, $S_{\pi } = \{x \in [N] \,:\, x \in \text {OC}_{k}\, \text {iff}\, \pi _{k}=1, k = 1,\ldots ,n\}- \text {USC}(\bar {h})$. Each subset in this partition constitutes a vertex in $\text {CG}(\bar {h})$. There is a directed edge (*u*, *v*) in $\text {CG}(\bar {h})$ if and only if *u*≠*v* and there exists *x*∈*u*, *y*∈*v* for which (*x*, *y*) is an edge in $G(\bar {h})$. The condensed graph is a directed acyclic graph.^7^ We assign a color to each vertex in $\text {CG}(\bar {h})$ and the same color to the points representing the EEs of the corresponding chains in the EE raster plots. Rows in the raster plots are permuted so that those belonging to the same CG vertex (and thus sharing the same color) are grouped together.

## Results

We created 90 model instances. Between them, both the chain strength variability parameter *G*_*σ*_/*G*_*μ*_, and the random structural parameters (couplings and relative chain strengths) were systematically varied. We created both FM and RM versions of each instance and conducted multiple runs of both. From the data we collected we are able to describe the nature of the observed activity patterns (Section 3.1) and apply the principle of effective connectivity to identify the components of the network structure that are predominantly responsible for the patterns of ongoing activity seen in each instance (Section 3.2).

**Fig. 3:**
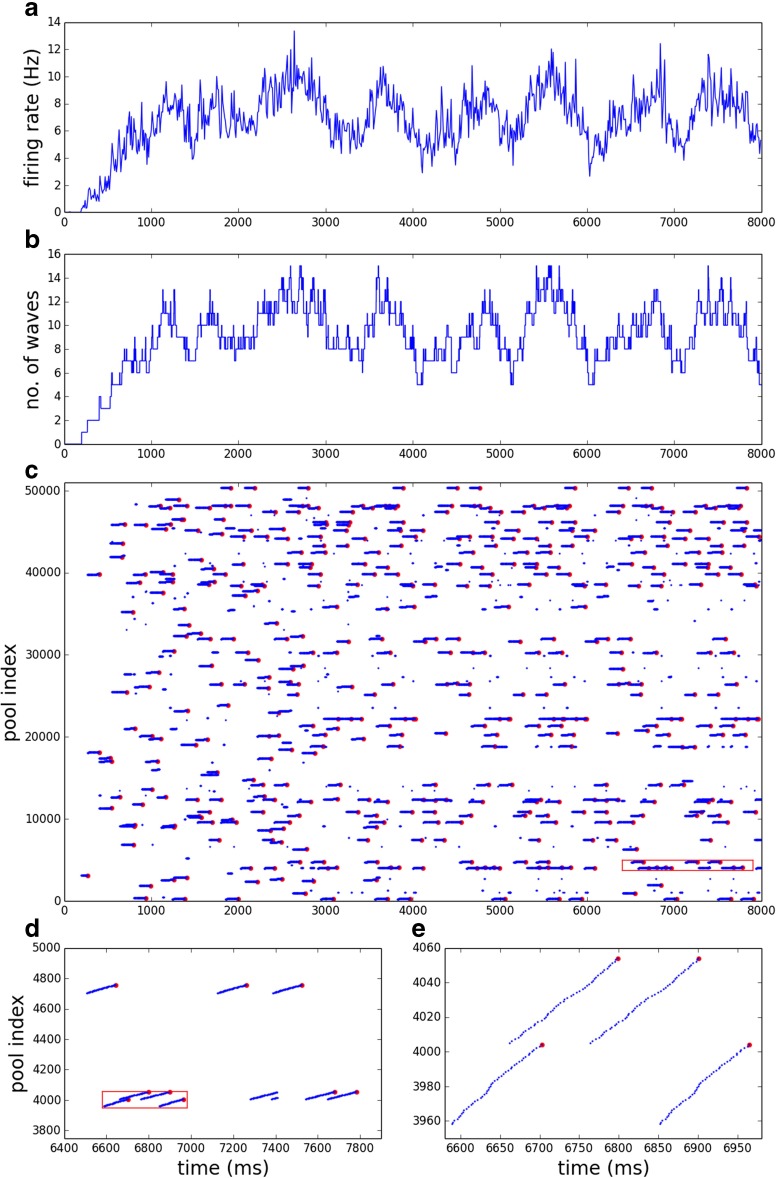
Ongoing activity versus time during the first 8000 ms of an FM run (RMP 3, *G*_*σ*_/*G*_*μ*_ = 0.3, run 8). **a**, **b**: firing rate and number of waves versus time. **c**: all detected pulse packets (*blue*) versus time. Those which are end events are also marked in *red*. **d**, **e**: enlargements of boxed areas in **c**, **d** respectively. Each *dot* in **c**–**e** represents a temporally localized packet of spikes in a pool of neurons

### The nature of ongoing activity patterns

#### **Ongoing activity**

Both FM and RM versions of the system maintain ongoing endogenous activity in the form of propagating synfire waves. Figure 3 shows the first 10000 ms of a typical FM run. After stimulation by a single pulse packet, the number of waves initially increases steadily with time, due to the branching coupling structure. After a few generations of branching (i.e. about one thousand milliseconds), the number of waves reaches an equilibrium where the wave-branching rate is counterbalanced by the extinction rate, due to the noise-mediated negative feedback (Fig. 3b). In this equilibrium, the number of waves fluctuates. While the fluctuations are modest on the time scale of a wave traversing the length of a chain (∼140 ms), over time scales of 1–2 seconds or longer the number of waves shows large fluctuations around the mean. The population averaged neuronal firing rate closely tracks the number or waves but is noisier (Fig. 3a).

Once equilibrium is reached, synfire waves propagate through the system in a quasi-random but patterned fashion, as the raster plot of pulse packets reveals (Fig. 3c). The bulk of the pulse packets occur in the course of successful chain traversals. Most unsuccessful traversals are brief. A run is therefore well-characterized by its set of end events (EEs) which mark successful traversals.

#### **Duration of ongoing activity**

For most FM and RM instances, in nearly all runs activity is sustained throughout (Supplementary Fig. 1). For a few runs in some RM and FM instances, activity is transient and dies out very early, before the equilibrium number is reached. For some FM instances there are runs in which activity persists for a long time but dies out before the end of the simulation.

For RM instances, transient activity is always of short duration. The percentage of RM runs exhibiting transient activity is zero at low chain strength variabilities and increases steadily to between 11 and 18 % of runs at *G*_*σ*_/*G*_*μ*_ = 0.4. The mean and maximum durations of transient activity increase with strength variability, but are always less than 50 ms and 900 ms respectively. Transient activity occurs either when the initially stimulated chain is too weak to sustain propagation even when only one wave is present, or has several very weak successors. Since it takes 110–160 ms to traverse a single chain, short durations are mostly of the former kind. Because the number of very weak chains increases with chain strength variability, so too does the duration and occurrence rate of transient activity.

The FM exhibits a comparable fraction of runs in which, for the same reason, activity dies out very early. However, at both zero and high strength variability a sizeable number of FM runs exhibit activity that survives for a large fraction of the run duration before dying out. At zero strength variability this is most likely because, on top of the expected firing rate for a given number of waves, the population-averaged firing rate – and hence the noise – also exhibits fluctuations (Fig. 3a). As a result, the noise can reach a level at which all waves simultaneously become highly susceptible to transmission failure. The explanation for the long transient runs at high strength variability will become more apparent in the next section.

#### **Mean number of waves**

**Fig. 4:**
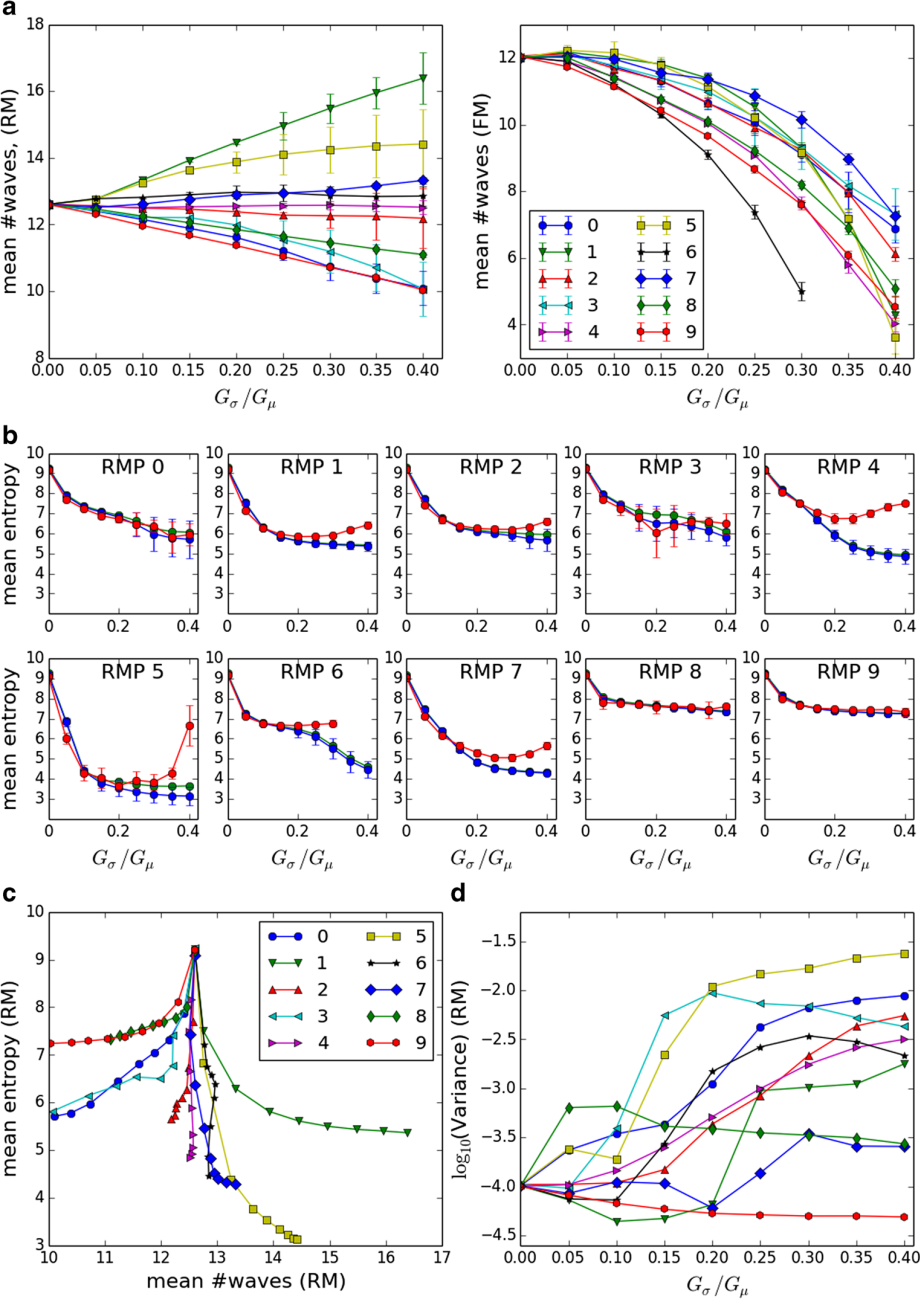
**a** Mean number of waves versus chain strength variability for RMPs 0,…,9, left panel FM, *right panel* RM; **b** Entropy of RM (*blue*) and FM (*red*) NEECs averaged over runs versus chain strength variability for RMPs 0,…,9. Also shown is the entropy of the mean NEEC (*green*). Error bars in A and B give standard deviation over runs. **c** Mean number of waves versus mean entropy for RMPs 0,…,9 and *G*_*σ*_/*G*_*μ*_ ∈ {0.0,0.05,…,0.4}. The curves for different RMPs diverge from the common point as *G*_*σ*_/*G*_*μ*_ increases. **d** Variance of RM NEECs over runs

While the number of waves is regulated, the mean number of waves varies systematically across models. Figure 4a shows for each RM and FM instance the mean number of waves per run averaged over all runs that exhibited ongoing activity for at least 10,000 ms. At zero strength variability the mean number of waves is almost the same across the 10 RMPs: 12.60 ±0.02 for the RM and slightly lower 12.03±0.08 for the FM. For the RM, as strength variability increases the mean number of waves exhibits a steady trend upwards or downwards depending on the RMP, starting from the common value of 12.6 at *G*_*σ*_/*G*_*μ*_ = 0. and reaching values between 10 and 16.4 at *G*_*σ*_/*G*_*μ*_ = 0.4. Similarly to the RM, for the FM the mean number of waves initially trends upwards or downwards depending on the RMP. However, this RMP-specific initial trend is soon replaced by an increasingly strong downward trend, at a rate that varies across RMPs. This explains why ongoing activity in the FM is more vulnerable to extinction at high strength variabilities than in the RM. This effect is exemplified by RMP 6, which shows the steepest drop in number of waves and the greatest number of transient activity runs at higher strength variabilities. The reasons for the substantial deviation between the RM and FM models at higher strength variabilities are discussed in Online Resource 1, Section 3.

#### **Steady state patterns of activity**

**Fig. 5:**
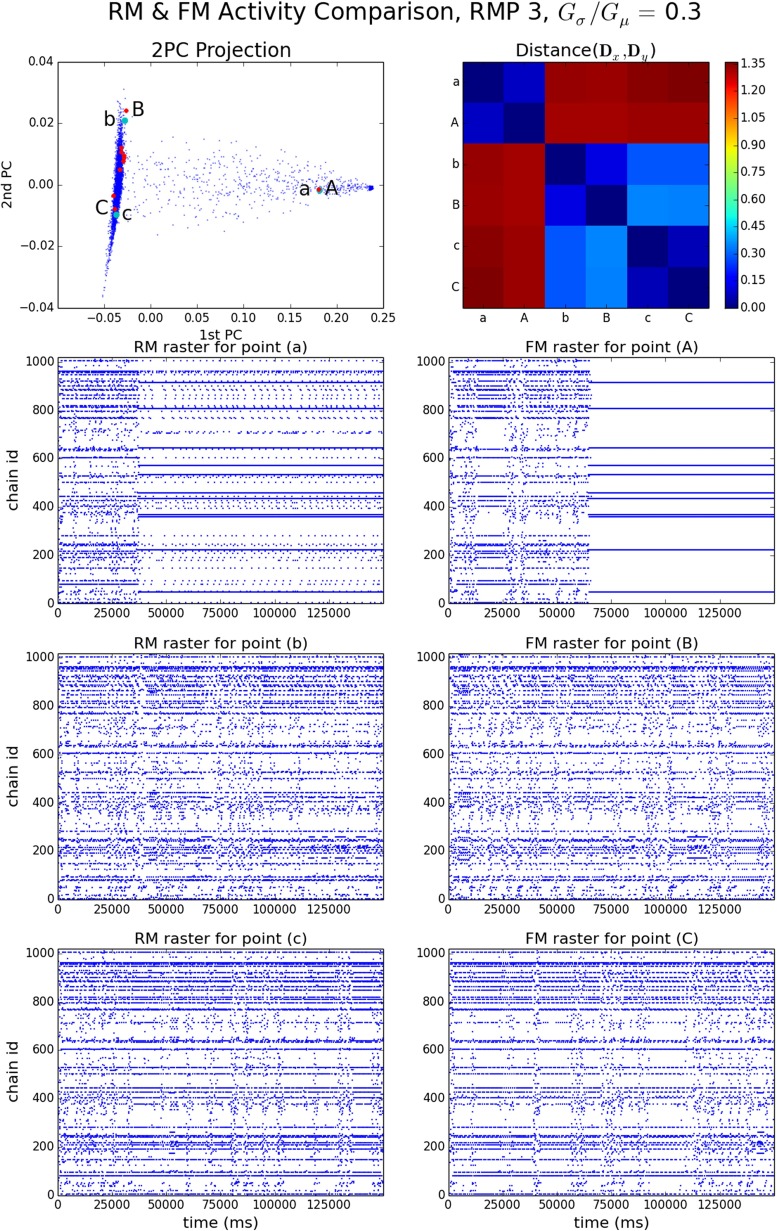
RM & FM EE activity for RMP 3, *G*_*σ*_/*G*_*μ*_ = 0.3. *Top left panel*: 2PC projection of NEECs of RM (*blue*) and FM (*red*) runs. Three RM runs (*green*, labels a, b, c) and three FM runs (labels A, B, C) are selected. *Top right panel*: matrix of distances between these six NEECs. *Remaining panels*: EE rasters for RM runs a-c (*left column*) and FM runs A–C (*right column*). FM run ’C’ is the run shown in more detail in Fig. 3

The set of EEs and the normalized vector of EEs per chain (NEEC) serve to characterize the patterns of ongoing synfire wave propagation. Figure 5 shows behaviors of a model instance with moderately high strength variability (RMP set 3, *G*_*σ*_/*G*_*μ*_ = 0.3), in both its FM and RM realizations. The 2PC projection is a plot of the first two PCs of the NEECs of all RM runs (blue) and FM runs (red) based on PCA of the RM data. The first two PCs account for more than 98 % of the RM run population variance (Supplementary Fig. 10). Three FM runs were chosen and each was paired with the RM run which minimized the L1 distance between their respective NEECs. Whereas the distances between individual RM runs can be substantial (in excess of 1 where the maximum possible distance is 2), those between the FM-RM pairs are much less. Raster plots of end events for the 3 RM and 3 FM runs are shown in the lower six panels of Fig. 5.

The EE rasters in Fig. 5 illustrate a generic feature of the system: it exhibits one or more distinct *steady states* in which it spends most of its time, while also making transitions between these states. A steady state can be characterized by a vector of end event counts per chain over its duration. More precisely, we identify a steady state as prevailing during in an interval of time *T*_ss_ = [*t*_0_, *t*_0_ + *T*] if for some choice of window *w*≪*T*, for any set of time-windowed end event count vectors $\{\mathcal {V}(t,w) : [t,t+w] \subset T_{\text {ss}}\}$ where $\mathcal {V}(t,w) = \left (|\{(t^{\prime },x)\in \mathcal {E} \, : \, t^{\prime } \in (t, t+w)\}|\right )_{x \in [N]}$, the variance of $\{\mathcal {V}(t,w)\}$ is small. Then $\mathcal {V}(t_{0},T)$ characterizes the steady state.^8^

As illustrated in Fig. 5, identification of steady states depends on window size. Using a long window of the order of, say, 20,000 ms, two distinct steady states are apparent. In the 2PC projection plot there are two dense clusters of points at the left and right extremes of the plot which correspond, respectively, to RM runs which spend almost all of their time in one or the other of these two steady states. RM runs that fall at intermediate locations (e.g. RM run ‘a’) spend some time in the left-cluster state before making a transition to the right-cluster state. The horizontal (1st PC coordinate) distance of a point from the right cluster is proportional to the fraction of time spent in the left-cluster state (and vice versa). Most RM runs lie within the left cluster. This suggests that the transition to the right cluster is a stochastic event with an expected waiting time much longer than the run duration. Using a short window such as 2000 ms reveals that the ‘left’ cluster steady state shows brief intermittent excursions from a relatively stable and predominant steady state to other, less stable and more dispersed configurations. The 2nd PC coordinate appears to reflect small variations in the mean duration and frequency of such excursions, these being visibly greater for runs ‘b’ and ‘B’ than for runs ‘c’ and ‘C’. Section 3.2 will shed light on the structures responsible for these excursions.

The behaviors of the 10 FM runs generally conform to those of the RM. Most FM runs and RM runs fall within the left RM cluster. However, the right cluster state seen in FM run ‘A’ is slightly more concentrated (lower in entropy) than the RM one seen in run ‘a’. The RM right state includes some activity on some chains that are highly active in the left state, while the FM right state does not. This is why the transition to the right cluster state occurs later in ‘A’ than in ‘a’, even though they have the same 1st PC coordinate. A putative right FM cluster, unobserved because of the small number of FM runs, would lie to the right of the right RM cluster.

#### **Behavior at zero strength variability**

In contrast to the multiple steady states and metastability seen in Fig. 5, at zero strength variability the behavior of the system is qualitatively the same for all RM and FM instances (RMPs). As illustrated by Supplementary Fig. 2 for RMP 4, each model instance exhibits a specific steady state, largely characterized by the mean NEEC over runs. Compared with the moderately large strength variability case shown in Fig. 5, the EE activity is distributed more broadly across chains (i.e. the entropies of the NEEC vectors are higher). The 2PC projection of the NEECs forms a single unstructured symmetric cluster around the mean, with small and approximately equal variances in the first two PCs. These two variance components are the same across RMPs. Moreover, the proportion of variance captured by the 2PC projection is very small (Supplementary Fig. 10), so distances in the 2PC projection are small and do not reflect the true distances between NEECs. While for the case of moderate strength variability in Fig. 5 the 2PC projection reveals large pairwise NEEC distances (≲1.35), at zero strength variability it does not. As we discuss further below, the variance of the NEEC population is uniformly low across models at zero strength variability, but is typically much higher for models with moderate to high strength variability. While the diversity of steady states in the latter results in large inter-cluster distances that are largely captured by the first 2 PCs, at zero strength variability pairwise distances are invariably more moderate (∼0.4). However, nearest neighbour distances are *larger* than at higher strength variabilities, because the NEEC vector population is less dimensionally constrained (Supplementary Fig. 11). In sum, the behavior at zero strength variability is both less structured and less dimensionally constrained, despite having a low variance compared with what is typically found when strength variability is present.

#### **Entropy**

Figure 4b shows the mean NEEC entropy across model instances. In the RM, the mean entropy shows a systematic tendency to decrease as variability in chain strengths increases. The overall decrease varies considerably across RMPs, dropping from a common value of about 9 at *G*_*σ*_/*G*_*μ*_ = 0 (c.f. the maximum possible value, log2(*N*)≈10) to between about 3.2 and 7.2 at *G*_*σ*_/*G*_*μ*_ = 0.4. The FM entropy is generally in good agreement with RM entropy, (unlike the mean number of waves), although for some RMPs at higher chain strength variabilities moderate to large discrepancies develop, with FM entropy rising relative to RM entropy.

There is a noticeable tendency for models with a higher mean number of waves to show lower NEEC entropies (Fig. 4c). This result is not surprising, as the waves making up that activity will be largely restricted to chains that are strong enough to be reliably traversed at that level of activity. Moreover, as proposed, wave activity will be restricted to islands of circulation among these chains. These islands will decrease in size as the level of activity increases, as noted in Section 2.4. Exactly how large these islands of circulation are depends on the chain strengths and the coupling structure of the model, which may go towards explaining departures from the overall trend in Fig. 4c such as the contrast between RMPs 1 and 5. This leaves open the question of why the specific pattern of couplings and chain strengths of one model instance leads to a high number of waves and low entropy NEECs, while that of another model leads to the opposite. We pick up these matters in Section 3.2.

#### **NEEC variance across runs**

The variance in the NEEC vectors is a measure of the diversity of behavior exhibited by a given model. It reflects the existence of multiple steady states, their frequency of occurrence, and how distant they are from one another. Across model instances, the variance of the population of NEECs varies greatly (Fig. 4d). At zero chain strength variability the variance takes a uniformly low value across RMPs and generally increases with strength variability, although not for all RMPs and not always monotonically. At moderate to high chain strength variability we observe high (RMPs 0, 3, 5), intermediate (RMPs 2, 4, 6) and low (RMPs 1, 7, 8, 9) variances. These range over nearly three orders of magnitude.

The same pattern is observed in the entropy results. Figure 4b shows, in addition to the mean RM NEEC entropy (blue), the entropy of the mean NEEC of the RM population (green). The entropy of the mean NEEC is necessarily greater than the mean of the entropies of the NEECs, unless the NEECs are all identical. Therefore the gap between the entropy of the mean NEEC and the mean entropy of the population serves as an indicator of variance in the NEEC population.

RM variance generally increases with strength variability while at the same time entropy decreases. This implies an increase in the diversity of steady states. At zero strength variability the system exhibits a single, homogeneous steady state of high entropy. In a steady state of high entropy, waves wander over a larger set of chains than in a low entropy steady state, so it takes longer to get an accurate estimate of the long term frequency of end events on each chain. Hence if a single steady state prevailed across a set of runs of fixed duration, the smaller the entropy of this state the smaller the NEEC variance would be. The fact that variance increases and entropy decreases with strength variability shows that at higher strength variabilities, the NEECs do not merely sample fluctuations in a single steady state, but encompass genuine diversity in steady states.

Further insight into the distribution of NEEC activity as a function of chain strength variability is gained by examining how the variance is distributed over PCs. For every RMP, the variances of the first few PCs increase with strength variability (not always monotonically), while the variances of the remaining components decrease (Supplementary Fig. 9). For most RMPs the former outweigh the latter and hence the total variance increases. Thus the first few PCs capture the increasing diversity of steady states. The proportion of the variance accounted for by the first 10 PCs is about 20 % at zero strength variability and increases monotonically with strength variability, attaining levels very close to 100 % for 8 of the 10 RMPs (Supplementary Fig. 10). At higher strength variabilities over 90 % of the variance is, for most RMPs, accounted for by the first 2 PCs. Of the two exceptions (RMPs 8 and 9), RMP 9 is a case in which the variance actually decreases, the contributions of the first few components increasing only modestly. Indeed RMP 9 is the only RMP in which variance decreases monotonically as entropy decreases. This is what we would expect if only a single steady state prevails over all runs, as indeed is the case, notwithstanding noticeable fluctuations on time scales up to about 5000 ms (Supplementary Fig. 8).^9^ RMP 8 is similar in behavior to RMP 9 apart from exhibiting, at non-zero strength variabilities, a minority of runs in which the predominant steady state is modified by the presence of activity on a self-looping chain; i.e. a chain which is its own successor (Supplementary Fig. 7). This accounts for the initial increase in the variance of RMP 8, largely captured by the first PC. Subsequently its variance declines like that of RMP 9.

#### **General features of 2PC projections**

As chain strength variability increases, the variance of the population of NEEC vectors generally increases while becoming more constrained to lie within a low-dimensional sub-space, with eventually most of the variance being captured by the first 2 PCs. Typically there is a shift from a single cluster of moderate variance corresponding to a steady state of high entropy, to multiple clusters with individually smaller variances, the corresponding steady states having lower entropy, while the total variance is considerably larger due to the separation of the clusters. Transitions between steady states appear to be stochastic, with a distribution of waiting times. When the mean waiting times for transitions between steady states are long compared with the run duration, NEECs sparsely fill the space between the corresponding clusters, and correspond to a minority of runs which exhibited a transition. In contrast, frequent bidirectional transitions between two steady states result in a single cluster of which the dimension of greatest variability reflects the relative amounts of time spent in each steady state.

#### **Comparison of FM and RM activity patterns**

We noted a systematic difference between the FM and RM models in the mean number of waves: in contrast to the RM models, the mean number of waves in the FM models fall increasingly sharply with increasing strength variability (Fig. 4a). The reasons for this discrepancy are discussed in Online Resource 1, Section 3.

Of complementary interest for the RM-FM comparison are the NEEC vectors, which being normalized, factor out the contribution of the mean number of waves. We compared the collections of NEEC vectors obtained over FM and RM runs respectively, and obtained a scalar measure of their difference, which we term Discrepancy. Discrepancy values are very low at low strength variabilities and, for some RMPs, at high strength variabilities too, even when there are large RM-FM differences in the mean number of waves. A large Discrepancy tends to occur when the mean number of waves in the FM model is very low and the RM entropy is low, and shows up as an increase in FM entropy relative to RM entropy. Even then, the set of chains upon which activity is essentially confined differs little between RM and FM; only the relative amounts of activity within this set differ. Thus an explanation of the distribution of activity in terms of islands of circulation may succeed for both RM and FM models even when the Discrepancy is high. See Online Resource 1, Section 2 for further details.

#### **Steady states reflect islands of circulation**

At moderate to high levels of chain strength variability, multiple steady states are predominantly observed, which may be stable or metastable (i.e. they transition to a different state at some point). The EE rasters and 2PC projections of a few of these are depicted in Supplementary Figs. 3–8. Some of these suggest that steady state patterns are due to propagation of activity on islands of circulation.

The distinct steady states of a model instance may sometimes be entirely dissimilar: their NEECs may be distant or indeed close to orthogonal. More often, however, they share some common component of activity. In RMP 3 *G*_*σ*_/*G*_*μ*_ = 0.3 (Fig. 5), we observed that certain very active chains in the high entropy ‘left’ RM state continue to be active, albeit at a reduced level, in the low entropy RM ‘right’ state. Similarly, in model instance RMP 6, *G*_*σ*_/*G*_*μ*_ = 0.35 (Supplementary Fig. 6) we see in runs a, b and c two metastable steady states, one of relatively low entropy and one of higher entropy, with transitions in both directions. This time very active chains in the low-entropy steady state continue to be active at a reduced level in the high-entropy one. Both cases suggest two islands of circulation joined by a one-way bridge. When the island that is upstream of the bridge is active, some activity can cross the bridge and activate chains in the downstream island. However, the downstream island’s activity has to compete with that of the upstream island. If activity on the upstream island dies out then the downstream island can maintain a higher level of activity.

Sometimes a transition between two steady states is mediated by a third one that endures for some interval in-between, and appears to be a superposition of the two. The NEEC of the EEs in the transition interval is essentially a weighted sum of the NEECs of the preceding and subsequent steady states. For instance, in RMP 5, *G*_*σ*_/*G*_*μ*_ = 0.2 (Supplementary Fig. 5), activity on one island of circulation manages to find its way to a second island, circulates on both islands for a while, then dies out on the first while continuing to on the second.

Two distinct steady states may sometimes look very similar. In RMP 8, *G*_*σ*_/*G*_*μ*_ = 0.15 (Supplementary Fig. 7) all runs look very similar but the 1st PC distinguishes from the majority of runs a minority exhibiting activity on three chains that are silent in the rest. These chains are a self-looping chain, its other successor, and one of the successors of the latter chain. They form a small island on which activity can circulate alongside the predominant steady state. This situation also occurs in RMP 5, *G*_*σ*_/*G*_*μ*_ = 0.2 (Supplementary Fig. 5).

Lastly, in some RM model instances (RMPs 1 and 5 with *G*_*σ*_/*G*_*μ*_≥0.25) activity is very occasionally confined to a single, strong self-looping chain. Such states have small basins of attraction: they occur only for the roughly 1/*N* runs in which the self-looping chain was activated by the initial stimulus. Despite being rare, such runs can have a disproportionate effect on the PCA, manifesting in the 2PC projection as outliers located well away from the rest of the run population (e.g. RMP 1, *G*_*μ*_/*G*_*σ*_ ∈ {.25,.3,.35,.4}, Supplementary Fig. 3).

#### **Spatiotemporal patterns and periodicity within steady states**

Steady states, being characterized by a vector of activity over a window of 2 seconds or more, leave ample room for variability in the spatio-temporal arrangement of waves. A variety of such arrangements of waves can be observed, which often may be periodic. Consider for instance the close up of the first 8000 ms of FM run C from Fig. 5 shown in Fig. 3. It reveals a periodic pattern of pulse packets and end events which emerges as part of the ‘left’ state, after 3000 ms, with a period of about 2000 ms. However, other patterns of activity occur in the ‘left’ state, as Fig. 5 shows. While periodic oscillations in the vicinity of 0.5–1.5 Hz are common, they tend to be transient. Low entropy steady states are the exception; e.g. RMP 5, *G*_*σ*_/*G*_*μ*_ = 0.4 exhibits stable oscillations of long duration. The basis for periodic oscillations is identified next, by effective connectivity analysis.

### Effective connectivity analysis

An *effective connectivity structure* (ECS) is the underlying graph of couplings (*G*_0_) in conjunction with $\{P(x|p(h|\bar {h})) ; x \in [N]\}$, the set of probabilities for successful traversal of the chains by a wave, taking into account the noise feedback due to fluctuating activity. From the ECS we obtain $G(\bar {h})$, a family of effective connectivity *graphs* (ECGs) parametrized by the mean activity level, $\bar {h}$ (Section 2.4).

Within each ECG we identify the components potentially responsible for the ongoing activity: the islands of circulation. Each island is the outcomponent (OC) of a strongly connected component (SC) within the graph (the OC being a superset of the SC) and their union is denoted $\text {UOC}(\bar {h})$. As $\bar {h}$ varies, a nested sequence of sets of islands results, each island at one $\bar {h}$-level being contained within an island at a lower $\bar {h}$-level. We let $\text {Size}(\bar {h})$ denote the normalized size of $\text {UOC}(\bar {h})$ and $\text {Frac}(\bar {h})$ the fraction of EE activity averaged over runs (i.e. the mean NEEC, $\bar {\boldsymbol {D}}$) that is located on chains in $\text {UOC}(\bar {h})$.

**Fig. 6:**
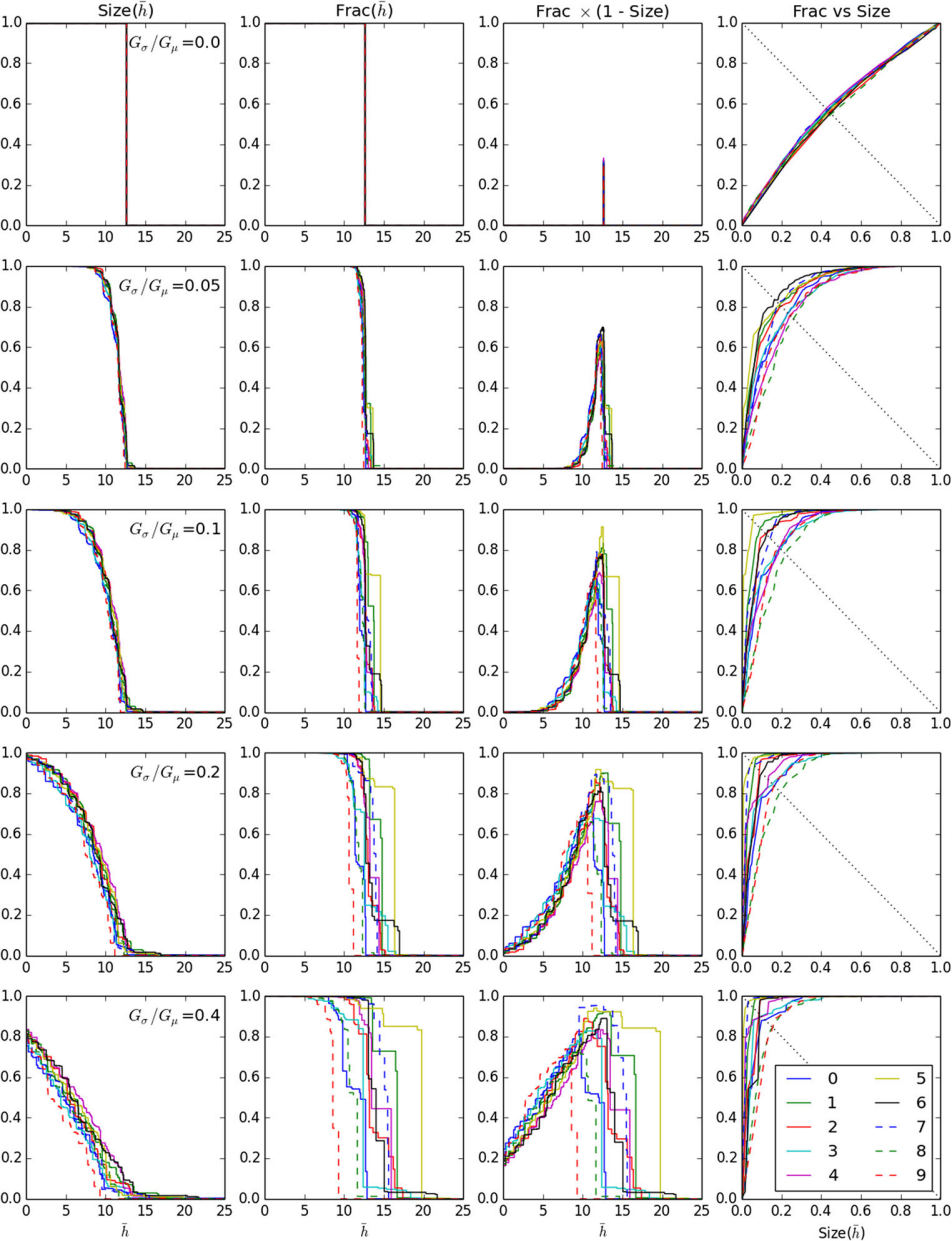
Relationship between EE activity and the activity-level dependent family of ECGs, $G(\bar {h})$. *Rows 1–5*: *G*_*σ*_/*G*_*μ*_ = 0.0,0.05,0.1,0.2,0.4 respectively. *Columns 1–4*: $\text {Size}(\bar {h})$ versus $\bar {h}$, $\text {Frac}(\bar {h})$ versus $\bar {h}$, $\text {Frac}(\bar {h}) (1 - \text {Size}(\bar {h}))$ versus $\bar {h}$, $\text {Frac}(\bar {h})$ versus $\text {Size}(\bar {h})$ respectively

Figure 6, first and second columns, shows Size and Frac versus $\bar {h}$ respectively for all RMPs at selected values of chain strength variability *G*_*σ*_/*G*_*μ*_ increasing as we go down each column. While both Size and Frac decrease with $\bar {h}$, Size decreases faster than Frac. Hence there exists a range of $\bar {h}$-values where Frac is high but Size is low. This is the range where the $\text {UOC}(\bar {h})$ best captures the observed distribution of activity in the system. This range is identified in the third column which shows Frac×(1−Size) versus $\bar {h}$, while the fourth column shows Frac versus Size. The favourable range is where Frac×(1−Size) attains high values and where the Frac-versus-Size curve approaches the top left corner of the plot.

For *G*_*σ*_/*G*_*μ*_ = 0 there is a very narrow interval of $\bar {h}$ values within which all chains exit $G(\bar {h})$ as $\bar {h}$ increases. In other words the landscape is almost flat; the remaining small variations are due to variability in chain lengths. As strength variability increases, the favourable range becomes broader and more pronounced. The variability in Frac and Size across RMPs increases with strength variability, but the pattern of differences across RMPs remains largely consistent.

This is because strength variability scales the $\bar {h}$ range over which chains exit $G(\bar {h})$. This scaling leaves invariant the *order* in which chains fall out (neglecting the minor effect of length variability). Thus the sequence of distinct graphs in $G(\bar {h})$ remains essentially the same as strength variability increases, but simply spread over a wider range of $\bar {h}$ values.

#### **How well does the**$G(\bar {h})$-**family account for activity?**

**Fig. 7:**
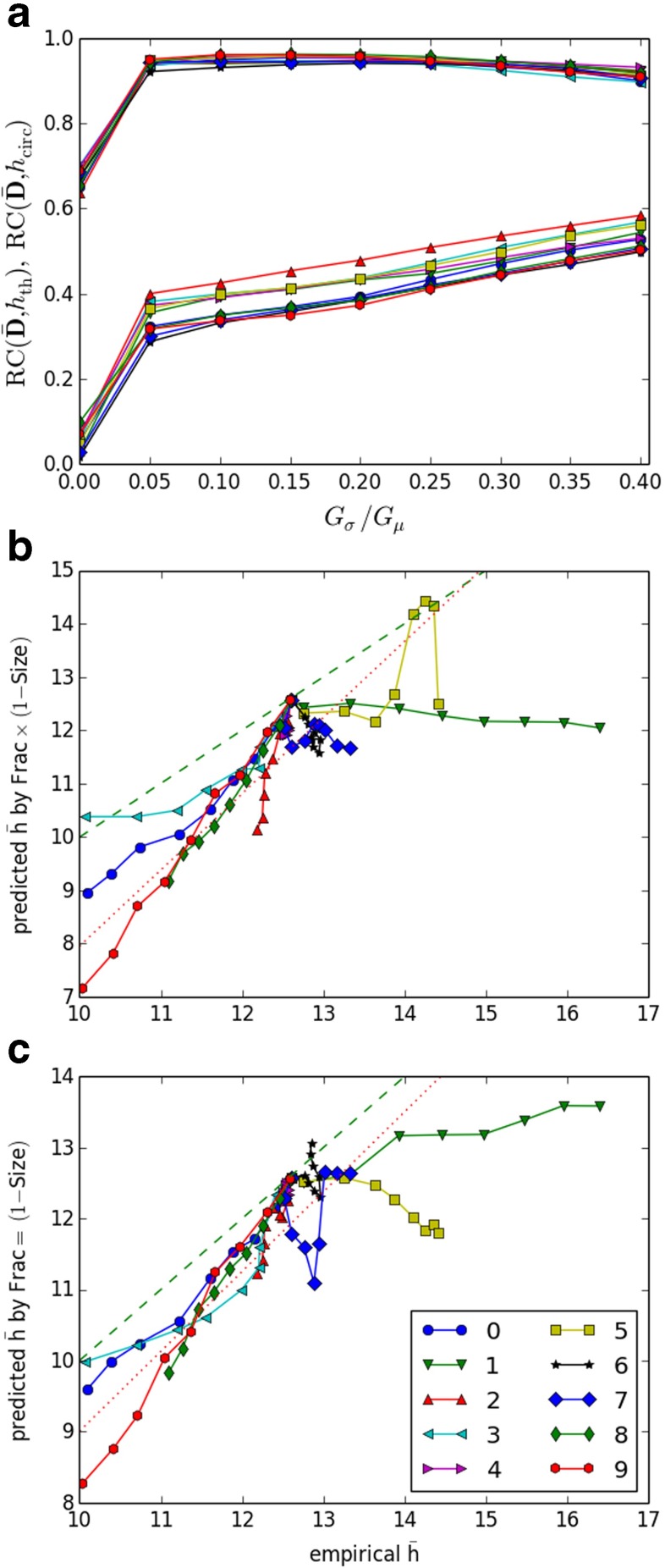
**a** rank correlation between chain activation frequency ($\bar {\boldsymbol {D}}$) and the $\bar {h}_{\text {circ}}$ measure of participation in $\text {UOC}(\bar {h})$ (*upper curves*) and between $\bar {\boldsymbol {D}}$ and $\bar {h}_{\text {th}}$ (*lower curves*). **b**, **c**: predicted mean number of waves ($\bar {h}$) versus empirical mean number of waves by (b) peak value of $\text {Frac}(\bar {h}) \times (1-\text {Size}(\bar {h}))$, (c) $\text {Frac}(\bar {h}) = (1-\text {Size}(\bar {h}))$; identity line, *green dashed*; regression line, *red dotted*. For each RMP the sequence of points for *G*_*σ*_/*G*_*μ*_ = 0.0,0.05,…0.4 is connected by line segments. The first point (*G*_*σ*_/*G*_*μ*_ = 0.0) is close to the identity line and is almost the same for all sequences

As $\bar {h}$ increases, the size of $\text {UOC}(\bar {h})$ drops. We can rank chains according to the value of $\bar {h}$ at which they drop out of $\text {UOC}(\bar {h})$, namely $\bar {h}_{\text {circ}}(x)$. We can also rank chains according to $\bar {\boldsymbol {D}}$, the normalized end event count averaged over runs. The rank correlation of these two rankings, $\text {RC}(\bar {\boldsymbol {D}}, \bar {h}_{\text {circ}})$, given in Fig. 7a, provides a measure of how well the $\text {UOC}(\bar {h})$ family captures the variations in the level of activity observed across the chains. The rank correlation is largely between 0.9 and 0.95 for all non-zero values of strength variability. At zero strength variability, it is still around 0.65–0.7. The distribution of activity across chains thus strongly mirrors the nested structure of islands of circulation in the $G(\bar {h})$ family. In contrast, we can rank chains purely by their wave-traversal effectiveness, which is measured by $\bar {h}_{\text {th}}$, the $\bar {h}$-value at which they drop out of $G(\bar {h})$. The rank correlation of $\bar {h}_{\text {th}}$ with $\bar {\boldsymbol {D}}$ is around 0.3–0.6 for non-zero values of strength variability, much less than that of $\bar {h}_{\text {circ}}$, indicating that the nested structure of islands of circulation in the $G(\bar {h})$-family explains the distribution of activity much better than the wave-traversal effectiveness of the chains alone.

#### **Relating optimal and empirical**$\bar {h}$**values**

How well does the ECG family $G(\bar {h})$ explain relative levels of activity seen across RMPs (Fig. 4a)? In particular, if we choose a value of $\bar {h}$ for which $G(\bar {h})$ is somehow *optimal* in explaining the observed distribution of activity over chains, does this value predict the empirical $\bar {h}$, or at least correlate with it across models?

To account for activity $G(\bar {h})$ must include SCs and hence $\text {Size}(\bar {h})$ must be non-zero. Figure 6 reveals large differences in the $\text {Size}(\bar {h})$ curves across RMPs when strength variability is large. RMPs 9 and 5 stand out as opposite extremes. At *G*_*σ*_/*G*_*μ*_ = 0.4, RMP 9 loses all islands of circulation at a low value of $\bar {h} \approx 9.3$ while such islands remain present in RMP 5 up to a high value of $\bar {h} \approx 23.2$. For comparison, the empirical $\bar {h}$ is approximately 10 for RMP 9 and 14.4 for RMP 5. In RMP 5, there are SCs in $G(\bar {h})$ at high $\bar {h}$-values; i.e. SCs comprised entirely of strong chains. In RMP 9, SCs are only present at much lower $\bar {h}$-values, and therefore include much weaker chains. Accordingly, RMP 9 equilibrates at a lower level of activity than RMP 5. However, the presence of an SC in $G(\bar {h})$ is, at best, only a necessary condition for an equilibrium at that level. To further constrain $\bar {h}$ we therefore take into account $\text {Frac}(\bar {h})$, the fraction of activity accounted for by the islands of circulation in $G(\bar {h})$.

We optimized $\bar {h}$ in terms of how well $G(\bar {h})$ accounts for the observed activity, using methods 1 and 2, (Section 2.4). Each of these is used to predict the equilibrium level of activity in the system. Comparisons between predictions and the empirical mean activity are shown in Fig. 7b and c. The correlations between the predicted and actual levels of activity, across all model instances, are 0.75 and 0.82 for methods 1 and 2 respectively. While these correlations are substantial, in absolute terms neither method is very good at predicting the empirical $\bar {h}$. Both methods give the right answer at zero strength variability and tend to introduce a negative bias into the way $\bar {h}$ trends as strength variability increases. Both methods predict the empirical $\bar {h}$ better when it decreases with strength variability than when it increases. Oddly, method 2 predicts that for RMP 5 $\bar {h}$ will decrease with strength variability where empirically it increases. However it is better than method 1 at predicting the increase in the empirical $\bar {h}$ for RMP 7.

In short, our methods for predicting the equilibrium level of activity based on the properties of the $\bar {h}$-family of ECGs are only partially successful. Ideally one would like a dynamical systems explanation. A reduced dynamical system based on activation rates might provide this, as we discuss in Section 4.

#### **Using condensed graphs to interpret EE rasters**

Suitably chosen ECGs reveal much about the organisation of steady state activity patterns and the structures underlying them. To aid in visualizing the structure of the ECG we use a convenient reduced representation, a condensed ECG (cECG).

**Fig. 8:**
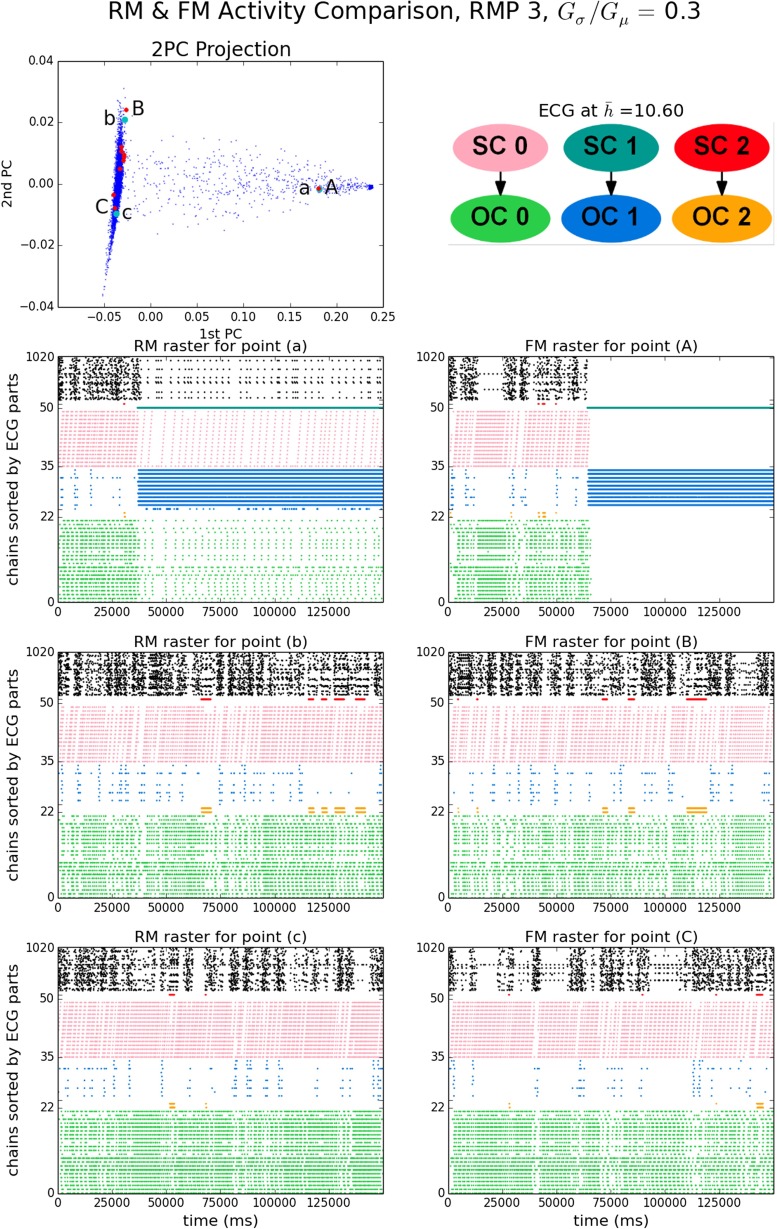
Condensed ECG (cECG) for $\bar {h}=10.6$, 2PC projection of NEECs, and selected EE rasters for RMP 3, *G*_*σ*_/*G*_*μ*_ = 0.3. *Top left panel*: As in Fig. 5. *Top right panel*: cECG. *Remaining panels*: EE rasters of Fig. 5 with chains colored according the node in cECG to which they belong, and rows permuted so that chains in the same cECG node are adjacent

**Fig. 9:**
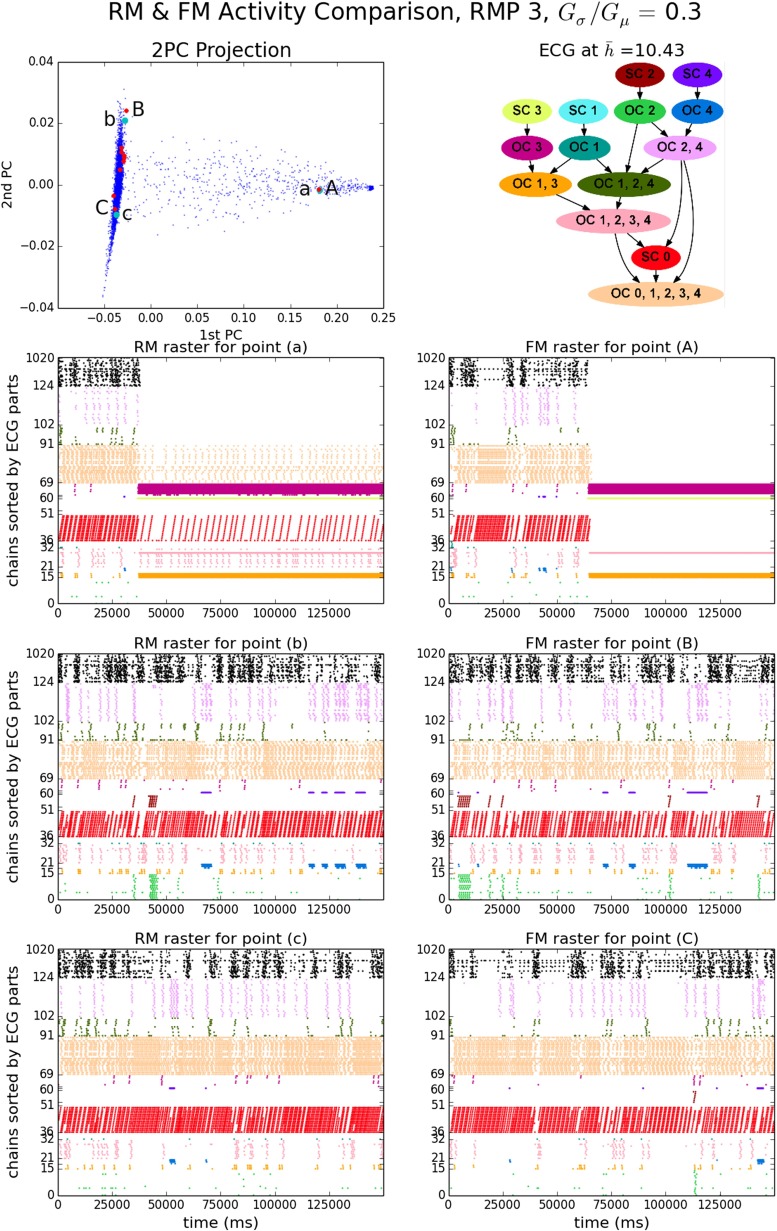
As for Fig. 8 except the ECG is chosen by the criterion $\text {Frac}(\bar {h}) = 1 - \text {Size}(\bar {h})$ giving $\bar {h}=10.43$

The choice of mean activity level, $\bar {h}$, has a large effect on what the condensed graph $\text {CG}(\bar {h})$ reveals. Consider the case of RMP 3 at *G*_*σ*_/*G*_*μ*_ = 0.3 which exhibits the ‘left’ and ‘right’ states as discussed earlier and depicted in Fig. 5. In Figs. 8 and 9, the same EE rasters are presented again, colored and sorted according to which SC or distinct OC part they belong to, along with the 2PC projection and the condensed ECG, for $\bar {h}=10.60$ and $\bar {h}=10.43$ respectively.

Consider the higher $\bar {h}$-level first. It identifies 521 supra-threshold chains as nodes of $G(\bar {h})$, of which 52 belong to the $\text {UOC}(\bar {h})$. These chains account for 78.3 % of the total EE activity obtained over runs (i.e. $\text {Frac}(\bar {h})$). In the upper portion of each raster plot and colored black are the EEs of all chains which do not belong to $\text {UOC}(\bar {h})$. The vertical extent of this portion is $(1 - \text {Frac}(\bar {h}))$ of the total, here and in all other figures showing EE rasters colored by ECG parts.

We see that large parts of the EEs constituting the left and right states, respectively, can be distinguished from one another according to which parts of the ECG they belong to. The left state is substantially due to recurrent activity on SC 0 (pink, 15 chains) which flows out to OC 0 (green, 22 chains). We determined that SC 0 is a simple loop. The diagonal lines of pink dots in the figure are paths of propagation around the loop. This path turns out to have a period of about 2009 ms and is therefore the origin of the periodic patterning of end events observed in Fig. 3, in which 3 waves simultaneously propagate on the loop.

A small amount of left state activity is due to intermittent bursts of recurrent activity on SC 2 (red), a single self-looping chain which activates the two chains in OC 2 (orange); this activity must contribute to the 2nd PC since it occurs considerably more often in ‘b’ and ‘B’ than in ‘c’ and ‘C’. However, a substantial amount of left-state EE activity is not accounted for by the ECG at this level (the EEs in black). The right state is, in the FM at least, entirely accounted for by recurrent activity on SC 1, a single self-looping chain (dark aqua) which activates the eleven chains in OC 1 (blue). In the RM, the right state also includes activation of SC 0 and OC 0, at a much reduced level to that which occurs in the left state. This difference explains why the 2PC projection of FM run ‘A’ is as far to the right as that of RM run ‘a’ even though the left state gives way to the right state much later in ‘A’ than in ‘a’. The condensed ECG at this $\bar {h}$ level does not show the pathways by which SC 0 and OC 0 continue to be re-activated in the RM right state. Nor does it show the pathway by which activity reached SC 1 at the point of transition to the right state.

When we move to the slightly lower threshold level ($\bar {h}=10.43$) the number of chains in the ECG increases from 521 to 538 and the $\text {UOC}(\bar {h})$ increases in size from 50 to 124 and accounts for 86.7 % of the total EE activity. Due to the additional chains a considerably richer structure of SCs and OCs can be observed. There are now five SCs instead of three. SC 0 and OC 0 remain unchanged (now colored red and salmon, respectively) and OC 0 is labeled OC 0,1,2,3,4 because it is now also reachable from SCs 1, 2, 3 and 4. The former SC 1 becomes SC 3 (lime) the former OC 1 breaks up into OC 3 (magenta, 7 chains), OC 1,3 (orange, 3) and 1 chain of OC 1,2,3,4 (pink). The other 10 chains of OC 1,2,3,4 are new additions to $G(\bar {h})$, as are SC 1 (sky blue, 2), OC 1 (dark green, 4), SC 2 (brown, 7), OC 2 (green, 15), OC 1,2,4 (khaki, 11) and OC 2,4 (mauve, 22). The former SC 2 becomes SC 4 (indigo) and OC 2 becomes OC 4 (blue), increasing in size from 2 to 3.

With its increased size and complexity, this condensed ECG explains more features of the steady states. We now see an additional component of left state activity due to brief transient activation of the new SC 2 and OC 2, which contributes to the 2nd PC differences between runs ‘b’ and ‘B’ and runs ‘c’ and ‘C’ in the same way as SC 4 (previously SC 2) does. The ECG now explains the component of intermittent activity on SC 0 and OC 0 in the RM right-state (run ‘a’). Close inspection of the raster for run ‘a’ reveals a stereotypical pattern. Each wave of activity on SC 0 in the right state is triggered by a burst of activity on OC 1,2,3,4, presumably driven by the highly active pathway from SC 3 via OC 3 and OC 1,3. The wave on SC 0 and the resulting activity on OC 0,1,2,3,4 appears to be responsible for terminating the OC 1,3,4 burst as it does not re-occur until after the wave on SC 0 ends. The wave on SC 0 appears to bring about its own termination after one cycle by producing a peak in activity just as it is about to complete the cycle.

Still, this ECG is unable to explain all the activity on the chains it includes. Some of the activity on OC 2,4 (mauve) during the left state is not due to SC 2 or SC 4 activity, and must therefore be brought about by activity on chains that are not included in this ECG. Likewise, during the left state there is activity on OC 3, OC 1,3 and OC 1,2,3,4 which cannot be traced via paths in the ECG from SC 0, the source of ongoing left state activity.

As the threshold is lowered further, and the ECG grows, more SCs and OCs are present but at the same time the ECG becomes complex and hard to interpret. With further reduction the result is distinctly sub-optimal: the ECG grows but components are merged. This merging is misleading, since they are activated independently in different steady states.

Further examples of ECGs and the steady state activity structures they reveal are given in Supplementary Figs. 13–17.

We conclude that, while a single ideal ECG is impossible to find, there is an optimal range of ECGs which sheds light on what is going on. An optimal ECG captures the most probable paths of propagation within the system, and there is a strong relationship between the islands of circulation and the steady states. This relationship is not in general a simple one-to-one mapping. Rather, each steady state has a distinctive SC signature: the SCs which are active in that steady state. Coupling and competition between islands of circulation is observed. For instance, when the ECG includes two SCs, a ‘lower’ one in the OC of the ‘upper’, two steady states may be observed: one in which the upper SC dominates with a small amount of activity percolating into the lower; another in which the lower SC is strongly active while the upper one is silent. Such competition is seen for example in RMP 3, *G*_*σ*_/*G*_*μ*_ = 0.3 (Fig. 9), as well as in RMP 2, *G*_*σ*_/*G*_*μ*_ = 0.3; RMP 4, *G*_*σ*_/*G*_*μ*_ = 0.4 and RMP 6, *G*_*σ*_/*G*_*μ*_ = 0.3 (Supplementary Figs. 13, 14 and 16). Co-existence of activity independently circulating on multiple islands of circulation is seen in RMP 2, *G*_*σ*_/*G*_*μ*_ = 0.3; RMP 5, *G*_*σ*_/*G*_*μ*_ = 0.2 and RMP 9, *G*_*σ*_/*G*_*μ*_ = 0.3 (Supplementary Figs. 13, 15 and 17).

Note that although the condensed ECG is acyclic, sub-threshold chains (which do not form part of the ECG) provide routes for recurrent circulation outside of the cyclic SCs. Successful traversals of sub-threshold chains are indicated by the presence of black-colored points in the colored EE raster. Indeed, while some transitions between steady states can be explained simply by the chance extinction of activity on an SC, others must involve traversals of chains which do not form part of the ECG. If there is no route in the condensed ECG from the active SCs to an inactive SC then the latter can only be activated by traversal of sub-threshold chains.

## Discussion

The cortical connectome may include an extensive system of meso-scale circuits of a certain kind: ones in which streams of spike activity propagate by means of synchronously converging inputs and interact via inter-circuit couplings. As an exemplar of such a system, we studied a model consisting of a large number of synfire chains cortically embedded in random superposition and linked by a random recurrent network of sequential couplings.

A basic property of the embedding is that propagating pulse packets generate background noise, which acts as a negative feedback signal that regulates the total number of pulse packets (Trengove et al. 2013b). Our first result is that the network of couplings gives rise to ongoing endogenously regenerated pulse packet activity. The creation and extinction of packets produces a fluctuating equilibrium in which multiple waves of propagating activity explore the structure in parallel in a quasi-stochastic fashion.

Ongoing activity consists chiefly of steady states that may be characterized by the relative mean rates of successful traversals of chains over intervals of 1–2 seconds or longer; that is, on time scales an order of magnitude greater than the mean chain traversal time of ∼140 ms. Variability in strength between chains induces an effective connectivity topography on the system. For models with zero strength variability, ongoing activity consists of a single steady state of high entropy. With moderate variability, the system typically exhibits multiple steady states of lower entropy, along with stochastic transitions between them. Durations of steady states range from too short to be characterized by traversal rates to much longer than the simulation duration. Within a steady state there is both variety and regularity, sometimes including periodicity, in the precise patterning of chain traversals. These patterns are reflected in large fluctuations in the number of waves. Indeed oscillations in the mean number of waves in a frequency range of 0.5–2 Hz are common.

Despite the complex structure of activity, the main characteristics of steady states can be largely understood in terms of a dynamically tuned *effective* meso-scale structure. Within this structure we identify effective connectivity graphs (ECGs) which distinguish chains of high traversal probability from the remaining chains. Within these graphs we find multiple islands of circulation, each such island being a strongly connected component (SC) along with its associated out-component (OC). While the SCs of different islands cannot overlap, their OCs may overlap, and the OC of one island may include the SC of another. These relationships are captured by a directed acyclic graph: the condensed ECG.

Within a suitable range of equilibrium levels of activity, the islands of circulation characterize the observed steady states, in the sense that most activity in a steady state is confined to one or more islands of circulation. Different steady states are distinguished according to which islands of circulation are active. Some transitions between steady states can be understood in terms of the extinction of activity on one SC while leaving activity on other SCs intact, while other transitions rely on the occasional successful traversal of sub-threshold chains lying outside of the ECG. Periodic temporal patterns arise when activity is driven by an SC that is a simple loop. In this case the period equals the time taken to traverse the loop.

When strength variability is low the system exhibits a single steady state of high entropy, while at higher strength variabilities multiple steady states of low entropy are exhibited. This contrast can be understood in terms of the scale of the topography relative to that of activity fluctuations. As per Eq. (5), activity fluctuations blur the sharp transition in traversal probability seen in Fig. 2a. When variations in topography are small compared with fluctuations in activity level they do not provide a clear distinction between reliable and unreliable chains, and the structure of islands of circulation is not robust. Propagation over the landscape is not effectively constrained, and the basis for multiple steady states dissolves.

A generic property of random directed graph models parametrized by the mean vertex degree is the emergence of a giant SC (i.e. one of order *N* in size) when the mean vertex degree rises above a threshold value (Newman (2010) and references therein). This phenomenon occurs in the $\bar {h}$-families of ECGs of our networks. In $G(\bar {h})$ the mean vertex degree decreases as $\bar {h}$ increases and nodes are pruned. Consequently, a giant SC is present at low $\bar {h}$, SCs are small in a critical $\bar {h}$ region, and no SC at all is present at high $\bar {h}$. Indeed, the $\text {Size}(\bar {h})$ curves in Fig. 6 signal that all of our systems cross the threshold for the emergence of the giant strongly connected component within the $\bar {h}$-range shown. When $\text {Size}(\bar {h})$ is large it is predominantly due to the giant OC (the OC of the giant SC). The ECGs that optimally characterize activity patterns occur where $\text {Size}(\bar {h})$ is small, i.e. where the largest SC exists but is small, and thus lie in the vicinity of the threshold for emergence of the giant SC. The appearance of short loops is also predicted to occur at this threshold (Newman 2010). This agrees with what we see in our optimal ECGs – most SCs are short loops, commonly a self-looping chain, while there is usually one rather large SC present, often dominating the activity (e.g. SC 0 in Figs. 8 and 9). Since a threshold for large-scale connectedness occurs in many random graph models, the tuning of the effective connectivity to lie near this threshold may be common to many models of coupled synfire chain landscapes.

The metastable steady states found in the present model are quite different to those recently exhibited in another balanced random recurrent cortical network model (Litwin-Kumar and Doiron 2012). In that model, a small fraction of excitatory connections have been rewired so as to form weakly segregated clusters. The metastable states involve activation of specific clusters at a higher rate of asynchronous irregular spiking than the rest of the network: rate-coding rather than temporal coding. Absent is the fine-grained meso-scale pool structure and precise timing relations found in the present model, along with the combinatorial potential.

### Limits to the effective connectivity analysis and potential improvements

The present effective connectivity analysis cannot explain in a principled way how the dynamical system comes to equilibrate at a particular level of activity. Our method of finding the islands of circulation responsible for the observed steady state patterns of EE activity partially explains why one model equilibrates at a higher or lower level than another. However, a causally closed explanation of equilibrium states, for instance in terms of attractor states of a reduced dynamical system, would be preferable.

This could perhaps be achieved via a reduced dynamical system based on a vector of mean rates of wave traversals over chains. Such an analysis would involve self-consistency equations for an equilibrium vector of mean rates together with an equilibrium probability distribution for the number of waves (*p*(*h*)) in a steady state. Nevertheless, it is doubtful whether an analysis at the level of mean rates will succeed in identifying the steady states correctly. It ignores the precise timing relations found between wave traversals within a steady state. These may not be essential to the steady state, since variations in the relative timing and frequency of traversals are often observed within steady states. However, because they determine the time course of the number of waves, ongoing circulation of activity may depend upon certain chains being traversed reliably during ebbs in the number of waves. In that case the set of time-averaged traversal probabilities would be inadequate to explain the steady state.

Another limitation of the method is that it can only produce ECGs that are acyclic. Suppose the system exhibits bidirectional transitions between two or more steady states, such that each is reachable from the others. Intuitively, one would like to capture this with an ECG in which there is a pathway from the island(s) of circulation associated with any one steady state to those associated with any other steady state. However this is ruled out by the very definition of an island of circulation as an SC plus its OC. If all the pathways mediating the steady state transitions are present in the ECG then the chains active in the mutually reachable steady states must merge into a single SC. In principle this limitation could be overcome by a two-stage analysis. After identifying and defining the SCs in an optimal ECG, the ECG could then be increased in size until it includes all desired pathways between the SCs already found in the optimal ECG. It might then be possible to define a condensation of the enlarged ECG in terms of the optimal SCs and thereby obtain a condensed ECG containing cycles.

Despite these limitations, the qualitative validity of the effective connectivity analysis is clear. By considering the topography of the coupled system in conjunction with the noise level, we can identify islands of circulation that to a large extent account for the steady state patterns of activity, and the observed transitions between steady states.

### Generalization of structure and dynamics and model extensions

We propose that the principle of a dynamically tuned effective connectivity structure, which both arises from and shapes ongoing activity, will be applicable to a much broader class of large-scale cortical networks: those which contain meso-scale structure that promotes propagation of spiking activity based on input-synchrony.

Within this broad class lie generalizations of our own models. The local circuit form of our models can be generalized from simple chains to structures such as polychronous assemblies (Izhikevich 2006), chains that admit multiple modes of wave propagation according to the timing patterns within pulse packets (Hopfield 1995; Maass and Natschläger 1997), and chains that are laterally extended with a Mexican-hat lateral coupling profile (Hamaguchi et al. 2005). As well as the sequentially excitatory couplings used here, there can be excitatory lateral couplings (Arnoldi and Brauer 1996; Abeles et al. 2004; Schrader et al. 2010) with varying degrees of temporal offset. Inhibitory couplings between chains could be included to implement mutually exclusive bindings through synchrony (Trengove 2006). All of these local variants can potentially exist together in the same local circuitry.

The meso-scale coupling structure may also be generalized. Instead of the present random network of couplings, the network may incorporate properties such as the small world property and hierarchical modularity, as found to emerge in certain neuron network models (Gong and van Leeuwen 2004; van den Berg and van Leeuwen 2004; Rubinov et al. 2009). In particular, within a complex network a module consisting of multiple densely inter-coupled local circuits could be organized according to the principle of diverging and converging pathways, whereby the propagation time over multiple poly-synaptic paths between two neurons or pools is the same, just as is the case within individual synfire chains and polychronous circuits (Bienenstock 1996). A system containing such temporally ordered modules would be in contrast to the present coupling system, in which waves that diverge from a common ancester chain rarely converge *simultaneously* on a common descendant chain. In the envisaged systems, noise feedback could in principle enable dynamic switching of effective connectivity between globally well-coupled small-world states and large-world states consisting of segregated modules (Trengove 2006).

Note that the role of background noise in restricting pathways of propagation on a landscape applies equally well to propagation via lateral couplings between chains, as modeled in a prototype RM formulation (Trengove et al. 2013a) based on FM simulations of pairs of laterally coupled chains (unpublished results). When there is variability in the dispersion of transmission delays within links, noise is selective for links with smaller dispersion. Likewise in the aforementioned modules containing diverging and converging pathways, those with less temporal dispersion will be more robust against noise.

The model may provide a new way to describe the neurodynamics underlying perceptual switching phenomena (Alais and Blake 2005); e.g. ambiguous figures (Ward and Scholl 2015) or binocular rivalry (Wade 1996). These are often modeled as bistability in the rate of activity in two neural populations, with competitive interaction. The present model suggests a different interpretation: alternation between activity on two islands of circulation, including brief periods when both patterns are active. Each mode of circulating activity will produce a distinctive profile of firing rates on the constituent neurons, which will depending on the chain embedding scheme (that is, the mapping from pools to neurons). Such a model would be in accordance with observations that perceptual switching takes time (in the order of 1 second) to complete and that there are multiple scenarios leading to a switching response (Nakatani and van Leeuwen 2006).

A further extension of the present network of embedded chains would be one which better reflects the spatial organization of the cortical connectome, by taking into account constraints on intracortical connectivity such as inter-layer connection densities (Thomson et al. 2002; Binzegger et al. 2004) and the hierarchical, layer-specific systematicity of cortico-cortical connections (Felleman and Van Essen 1991). The present model is concerned with intrinsic network topology ignoring constraints that arise from spatial organization. Given that the cortical network is left hugely undetermined by the known anatomical constraints alone, it is quite feasible for intrinsic network topology of the kind we suggest here to be present in a network that also meets those constraints. The assignment of neurons to pools and of links between pairs of pools in the embedding procedure could be statistically biased so as to meet the known spatial constraints on cortical organization.

While our model employs simple integrate-and-fire neurons with additive synaptic integration, it has recently been shown that incorporation of dendritic nonlinearities via non-additive summation increases the robustness of pulse packet propagation to balanced background input (Jahnke et al. 2013). Not only does this allow for more efficient embedding of chains; in such a model, balanced oscillations in background input can promote propagation on chains for which the pool-to-pool propagation time is a multiple of the oscillation period (Jahnke et al. 2014). In the present model, the large, random inter-link delay variability precludes any systematic utilization of this resonance effect. However, if the links within a given set of chains were to have nearly identical inter-link delays, their synchronous co-activation could induce a resonance effect that would facilitate propagation where it might otherwise fail: a novel kind of context-dependent activation.

## Electronic supplementary material

(PDF 197 KB)

(PDF 4.89 MB)

## Footnotes

1 In the original model the excitatory conductance response amplitudes (*g*_E, *j*_) all take the same value (*G*_*μ*_ in Table 1), but in the extension we describe below they will take different values according to the strength of the chain to which they belong.

2 Due to these couplings there is a net increase of *N* in the number of links. Hence the mean afferent excitatory connectivity increases slightly from *C*_E_ = 8000 to *C*_E_ = 8160 and *C*_I_ increases correspondingly.

3 Multiple waves may propagate successfully to the same pool simultaneously if they occupy the end pools of multiple chains having a common successor chain. In that case only a single wave will be activated at the target pool.

4 We choose to set *L* = *L*_0_ because it is the relevant length scale for the percolation of wave activity in the system. In the full model, the state of a pulse packet – its width and amplitude – follows a stochastic trajectory around an equilibrium and is correlated from one pool to the next, with extinctions resulting from excursions beyond the basin of attraction of stable propagation (Diesmann et al. 1999). Hence the relation $P_{\mathrm {s}}(h,g_{\mathrm {E}}, L^{\prime }) = P_{\mathrm {s}}(h,g_{\mathrm {E}}, L)^{L^{\prime }/L}$ which is true by definition for the RM is not necessarily true for the FM. The binary propagation probability in the FM should therefore be estimated at the length scale at which we want RM-FM agreement.

5 Setting the conductance values of all excitatory synaptic noise events to a single value *g*_E_ = *G*_*μ*_ is a simplification, since the strengths of the input synapses of a neuron will follow a distribution approximately the same as that of the chain strengths, *N*(*G*_*μ*_, *G*_*σ*_). The impact of this simplification is discussed in Online Resource 1, Section 3.

6 We set the pruning threshold to a moderately high value of *θ* = 0.8. Our rationale for this choice is that a chain should belong to the ECG if and only if it can contribute *reliably* to the circulation of ongoing activity constituting the steady state.

7 The proof relies on the way the vertices in CG are defined. All vertices of G in a given vertex of CG share the same profile of which SCs they are reachable from. If there was a cycle including 2 or more of the vertices of the CG, all these vertices would mutually ’infect’ each other with the property of being reachable or not from any given SC. Hence according to the definition of *S*_*π*_ they would have to belong to the same *S*_*π*_ which is a contradiction. On the other hand, there are no cycles of length one in the CG. Each SC in G would be a self-looping vertex in CG, but these are by convention excluded from the definition, as in the standard condensation of a directed graph (Yellen 2013).

8 This definition simply serves to explain what we mean by a steady state when we say that one is visually apparent in the data. We do not identify the steady states present in our data explicitly, although one could do so, for instance by cluster analysis.

9 RMP 9 is also distinctive in that it has the lowest mean number of waves. The reason for this will become apparent when we look at its effective connectivity structure in Section 3.2.
