# Supplementary material for: Dynamic effective connectivity in cortically embedded systems of recurrently coupled synfire chains
Source: J Comput Neurosci. 2015 Nov 11;40:1–26. doi: 10.1007/s10827-015-0581-5 (PMC4762935; doi:10.1007/s10827-015-0581-5)
Supplement: Supplementary file 1 — (PDF 197 KB) [file 10827_2015_581_MOESM1_ESM.pdf]

Online Resource 1: Comparison of Full and Reduced Models

for Journal of Computational Neuroscience article
Dynamic effective connectivity in cortically embedded systems of recurrently coupled synfire chains

Chris Trengove[1], Markus Diesmann, Cees van Leeuwen

1 The relationship between background input and number of waves

To translate $\hat{P}_{\mathrm{s}}(\lambda_{\mathrm{E}}, g_{\mathrm{E}}, L_0)$, which models wave propagation in the FM, into $P_{\mathrm{s}}(h, g_{\mathrm{E}}, L_0)$, which models wave propagation in the RM, we need λ_{E} as a function of h, the number of synfire waves. For the model of Trengove et al. (2013) this function $\lambda_{\mathrm{E}}(h)$ is determined by

$$\lambda_{\mathrm{E}} = C_{\mathrm{E}}(\nu_{\mathrm{S}}(\lambda_{\mathrm{E}}) + \nu_{\mathrm{W}}(h, \lambda_{\mathrm{E}}))\ , \tag{1}$$

where $\nu_{\mathrm{S}}(\lambda_{\mathrm{E}})$ is the rate of stochastic spiking per neuron in response to background input – its value is determined numerically by simulations of single neurons receiving background input of the same form as used in the simulations of individual chains. $\nu_{\mathrm{W}}(h, \lambda_{\mathrm{E}})$ is the rate per neuron of spikes belonging to waves, in Trengove et al. (2013) given by

$$\nu_{\mathrm{W}}(h, \lambda_{\mathrm{E}}) = h n_{\mathrm{E}} p_{\mathrm{f}}(\lambda_{\mathrm{E}})/(N_{\mathrm{E}} T(\lambda_{\mathrm{E}}))\,, \tag{2}$$

where $n_{\mathrm{E}} p_{\mathrm{f}}(\lambda_{\mathrm{E}})$ is the expected size of the pulse packet and $T(\lambda_{\mathrm{E}})$ is the mean pool-to-pool propagation time. Equations (1) and (2) determine λ_{E} as a function of h. However, in the present model, due to the variable chain strengths, equation (2) needs to be modified. The contribution to ν_{W} made by an individual wave, $\nu_{\mathrm{W},1}$, depends on the strength g_{E} of the chain on which it propagates:

$$\nu_{\mathrm{W},1}(\lambda_{\mathrm{E}}, g_{\mathrm{E}}) = n_{\mathrm{E}} p_{\mathrm{f}}(\lambda_{\mathrm{E}}, g_{\mathrm{E}})/(N_{\mathrm{E}} T(\lambda_{\mathrm{E}}, g_{\mathrm{E}}))\,, \tag{3}$$

where the dependencies of p_{f} and T on g_{E} and λ_{E} are determined via the same simulations that were used to determine $\hat{P}_{\mathrm{s}}(\lambda_{\mathrm{E}}, g_{\mathrm{E}}, L = L_0)$. Thus ν_{W} depends on the strengths of the specific chains on which the current set of waves $W(t)$ propagate:

$$\nu_{\mathrm{W}}(\lambda_{\mathrm{E}}) = \sum_{w \in W(t)} \nu_{\mathrm{W},1}(\lambda_{\mathrm{E}}, g_{\mathrm{E},x(w)})\,. \tag{4}$$

Eqns (4) and (1) determine λ_{E} as a function of the set of waves $W(t)$. However, we must approximate λ_{E} as a function of just the *number* of waves. To obtain this approximation, we note that Equation (4) can be written as

$$\nu_{\mathrm{W}}(\lambda_{\mathrm{E}}) = h n_{\mathrm{E}} \langle p_{\mathrm{f}}/T \rangle(\lambda_{\mathrm{E}})/N_{\mathrm{E}} \tag{5}$$

[1]Corresponding author, email: trengove.c@gmail.com

where $\langle p_\mathrm{f}/T\rangle(\lambda_\mathrm{E}) \equiv \langle p_\mathrm{f}(\lambda_\mathrm{E}, g_{\mathrm{E},x(w)})/T(\lambda_\mathrm{E}, g_{\mathrm{E},x(w)})\rangle_{w\in W(t)}$. Given a distribution $\rho(g_\mathrm{E};\lambda_\mathrm{E})$ for the strengths of the chains on which the current waves reside for a given background rate λ_E, we can calculate an expected value for $\langle p_\mathrm{f}/T\rangle(\lambda_\mathrm{E})$ with respect to this distribution:

$$\mathrm{E}\langle p_\mathrm{f}/T\rangle(\lambda_\mathrm{E}) = \int p_\mathrm{f}(\lambda_\mathrm{E}, g_\mathrm{E})/T(\lambda_\mathrm{E}, g_\mathrm{E})\rho(g_\mathrm{E};\lambda_\mathrm{E})\mathrm{d}g_\mathrm{E}\,. \tag{6}$$

We estimate $\rho(g_\mathrm{E};\lambda_\mathrm{E})$ using the heuristic that the underlying distribution of chain strengths in the network be weighted by the expected distance traversed by a wave at noise level λ_E before extinction:

$$\rho(g_\mathrm{E};\lambda_\mathrm{E}) = l(\hat{P}_\mathrm{s}(\lambda_\mathrm{E}, g_\mathrm{E}))\rho_0(g_\mathrm{E})/\int l(\hat{P}_\mathrm{s}(\lambda_\mathrm{E}, g'_\mathrm{E}))\rho_0(g'_\mathrm{E})\mathrm{d}g'_\mathrm{E} \tag{7}$$

where $\rho_0(g_\mathrm{E})$ is the underlying distribution of chain strengths, $g_\mathrm{E} \sim \mathrm{N}(G_\mu, G_\sigma)$, and $l(\hat{P}_\mathrm{s}) \equiv (\hat{P}_\mathrm{s} - 1)/\log(\hat{P}_\mathrm{s})$ is the expected fraction of a chain of length L_0 that will be traversed by a wave.

Replacing $\langle p_\mathrm{f}/T\rangle$ in Equation (5) with $\mathrm{E}\langle p_\mathrm{f}/T\rangle$ as given by Equations (6) and (7) we obtain an estimate for ν_W that depends on the number of waves:

$$\nu_\mathrm{W}(h, \lambda_\mathrm{E}) \approx h n_\mathrm{E}\mathrm{E}\langle p_\mathrm{f}/T\rangle(\lambda_\mathrm{E})/N_\mathrm{E}\,. \tag{8}$$

Equation (8) combined with Eqn (1) then determines h as a function of λ_E. This function is plotted in Fig. 2d for the 9 values of chain strength variability G_σ investigated (red curves).

We further simplified the λ_E-h relationship to remove the dependence on G_σ, noting that the curves are very similar over most of the domain and only begin to deviate from one another when $h \gtrsim 11$ ($\lambda_\mathrm{E} \gtrsim 65$ kHz), the curves for small G_σ deviating the most. Specifically, we replaced the λ_E and G_σ-dependent term $\mathrm{E}\langle p_\mathrm{f}/T\rangle(\lambda_\mathrm{E}, G_\sigma)$ in Equation (8) with its value when evaluated at $\lambda_\mathrm{E} = \lambda_{\mathrm{E,th}}(G_\mu)$, $G_\sigma = 0.0015$:

$$\nu_\mathrm{W} \approx h n_\mathrm{E}\mathrm{E}\langle p_\mathrm{f}/T\rangle(\lambda_{\mathrm{E,th}}(G_\mu), 0.0015)/N_\mathrm{E}\,. \tag{9}$$

Equation (9) combined with Eqn (1) determines the $\lambda_\mathrm{E}(h)$ function that we used in the RM update rule. This function is plotted (in blue) in Fig. 2d.

2 Comparison of RM and FM NEEC data sets

We compared the behaviors of RMs and their corresponding FMs on the basis of their respective sets of M_ρ NEEC vectors obtained from the M_ρ acceptable runs of each. The method of comparison needed to take into account the high dimensionality of the data ($N = 1020$), the small number of FM runs for each RM instance, and our observations that for many RM instances the set of NEEC vectors exhibits substantial variability while being constrained to fall approximately within a low dimensional subspace. Accordingly, we defined two scalar measures of distance between a target vector and the set of NEEC vectors of a given RM.

For the first measure, we defined the distance between a target vector $\boldsymbol{D}$ and the NEEC vector set for RM model ρ to be the shortest distance between $\boldsymbol{D}$ and any $\boldsymbol{D}' \in \mathcal{D}^\rho, \boldsymbol{D}' \neq \boldsymbol{D}$ under the L1 norm:

$$d_{\min}(\boldsymbol{D}, \mathcal{D}^\rho) = \min\{||\boldsymbol{D} - \boldsymbol{D}'||_1\,;\, \boldsymbol{D}' \in \mathcal{D}^\rho, \boldsymbol{D}' \neq \boldsymbol{D}\} \tag{10}$$

where $||\boldsymbol{D}||_1 = \sum_{k=1}^{N} |D_k|$.

For the second measure, we defined a function f^ρ which maps a target vector $\boldsymbol{D}$ into a plane defined by the mean of the NEEC vector set for RM model ρ and the first two PCs of the mean-subtracted NEEC vector set:

$$f^\rho(\boldsymbol{D}) = \sum_{k=1}^{2} \boldsymbol{E}_k^{\rho T}(\boldsymbol{D} - \bar{\boldsymbol{D}}^\rho)\boldsymbol{E}_k^\rho + \bar{\boldsymbol{D}}^\rho .$$

We refer to $f^\rho(\boldsymbol{D})$ as the '2PC reduction' of $\boldsymbol{D}$ with respect to RM model ρ. The 2PC reduction of RM vector set $\mathcal{D}^\rho$ itself, $\{f^\rho(\boldsymbol{D})\,;\, \boldsymbol{D} \in \mathcal{D}^\rho\}$ is a planar approximation of the NEEC vector set for RM model ρ. (In cases where RM outliers were found to have a disproportionate impact on the 2PC projection, the 2PC projection was obtained from PCA with the outliers removed.) We defined the distance between a target vector $\boldsymbol{D}$ and the NEEC vector set for RM model ρ to be:

$$d_{2\mathrm{PC}}(\boldsymbol{D}, \mathcal{D}^\rho) = ||\boldsymbol{D} - f^\rho(\boldsymbol{D})||_1 \, . \tag{11}$$

For each distance measure $d \in \{d_{\min}, d_{2\mathrm{PC}}\}$ we defined the set of distances between target vectors of model ρ' and the vector set of RM model $\rho = (\alpha, \gamma, \mathrm{RM})$:

$$d(\mathcal{D}^{\rho'}, \mathcal{D}^\rho) \equiv \{d(\boldsymbol{D}, \mathcal{D}^\rho)\,;\, \boldsymbol{D} \in \mathcal{D}^{\rho'}\} \, , \tag{12}$$

We computed two such distance sets according to target vectors being (a) the RM data set itself ($\rho' = \rho$), or (b) the *corresponding* FM data set ($\rho' = (\alpha, \gamma, \mathrm{FM})$). For reference we considered the union of distance sets $d(\mathcal{D}^{\rho'}, \mathcal{D}^\rho)$ obtained using the target vector sets $\mathcal{D}^{\rho'}$ of *non-corresponding* FM models with the same chain strength variability: $\rho' = (\alpha', \gamma, \mathrm{FM}), \alpha' \neq \alpha$.

Finally, we generated a single scalar measure of discrepancy between FM model ρ' and corresponding RM model ρ for each $d \in \{d_{\min}, d_{2\mathrm{PC}}\}$:

$$\mathrm{Discrepancy} = \frac{1}{2}\left[\langle d(\mathcal{D}^{\rho'}, \mathcal{D}^\rho)\rangle - \langle d(\mathcal{D}^{\rho}, \mathcal{D}^\rho)\rangle\right] \, . \tag{13}$$

The divisor of 2 is present because NEEC vectors are non-negative unit vectors in the L1 norm and hence the maximum distance between two NEEC vectors is 2 (which occurs when they are orthogonal).

Scatter plots of all distance sets $d(\mathcal{D}^{\rho'}, \mathcal{D}^\rho)$ are depicted in Supplementary Fig. 11. The difference between the means of distance sets (a) and (b) constitutes a single scalar measure of overall RM-FM mismatch. Referred to as Discrepancy, this measure is shown in Supplementary Fig. 12.

For all 90 RM-FM model instance pairs and for both distance measures the Discrepancy is positive. The Discrepancies for the two distance measure are usually very similar. Discrepancies increase with strength variability but the rate of increase varies greatly over RMPs. Discrepancies are below 0.05 for all models at low strength variabilities ($G_\sigma/G_\mu \leq 0.15$), and remain below 0.05 for some models at considerably higher levels of strength variability. In these cases the activity patterns (NEECs) found in the FM model are very similar to those typical of the RM, even when there are quite large differences in the mean number of waves.

Note that the normalization of the NEEC vectors factors out the contribution of the mean number of waves so that we compare vectors of *relative* amounts

of end event activity on chains. Nevertheless, the steep decline in the mean number of waves in the FM at high strength variabilities coincides with an increase in RM-FM Discrepancies. For instance, RMP 6 has the highest RM-FM discrepancy, its FM version exhibits the lowest mean number of waves of all RMPs at $G_\sigma/G_\mu = 0.3$, and for $G_\sigma/G_\mu \geq 0.25$ all its runs activity died out before the end, presumably being vulnerable to extinction due to the low mean number of waves. For $G_\sigma/G_\mu \geq 0.25$, RMP 6 gave no acceptably long FM runs on which to make the comparison.

A strong indicator of RM-FM discrepancy in NEECs is RM entropy: the five RMPs showing the greatest Discrepancy values (in descending order, RMPs 6, 5, 4, 7 and 1) also have the five lowest RM entropies at high strength variabilities. In these cases there is substantial RM-FM discrepancy in entropy (Fig. 4b). The increase in FM NEEC entropy relative to RM NEEC entropy is contributing to the overall RM-FM discrepancy. An example of high RM-FM discrepancy associated with high entropy discrepancy is RMP 4, $G_\sigma/G_\mu \geq 0.35$ (Supplementary Fig. 4). From visual inspection it appears that the minority of chains which are active is essentially the same for both the RM and FM runs and hence the increase in entropy must be due to a more uniform pattern of activation of this minority.

Some further observations can be made about the scatter plots of distance sets in Supplementary Fig. 11. Consider first the RM-RM distances, which form the baseline from which RM-FM differences are inferred. We find that RM target-to-population distances are generally low for the $d = d_{\rm min}$ measure, trending slightly downwards and becoming more heterogeneous as strength variability increases. The higher RM-RM $d_{\rm min}$ distances at zero strength variability imply that the data is spread over a larger volume, despite the low variance of the data at zero strength variability. This is a consequence of the data being less dimensionally constrained: the volume occupied by the data can be larger even though the variance is smaller. Likewise, the RM-RM $d_{\rm 2PC}$ distances also trend downwards with strength variability at low strength variabilities. This is a natural consequence of the planar approximation becoming more valid.

Next, consider the corresponding FM-RM distances. As noted, these are on average always higher than the RM-RM distances. We note that at zero strength variability there are seven outlier points of unusually high distance. These outliers break a pattern of otherwise good RM-FM agreement at $G_\sigma/G_\mu = 0$. These outliers are the seven runs with the shortest activity durations among the ten runs at $G_\sigma/G_\mu = 0$ with transient activity longer than 10,000 ms (top right panel of Supplementary Fig. 1). Their high distances arise merely because high entropy steady states require a longer duration of ongoing activity in order for the NEEC vector to converge to the long term mean. This effect was *not* responsible for the high distances at non-zero strength variabilities which, where present, reflect genuine RM-FM discrepancies.

By way of contrast, the distances between non-corresponding FM vectors and RM populations are always larger, and typically hover within a range not far below the value of 2, the maximum distance between NEEC vectors, attained when they are orthogonal; that is to say when they have zero overlap. Only for the two lowest values of strength variability are they somewhat lower: around 1.1 at $G_\sigma/G_\mu = 0.$ and 1.65 at $G_\sigma/G_\mu = 0.05$. This breakdown of orthogonality can be attributed to the higher entropy of NEEC vectors at low strength variability. Whereas two randomly chosen vectors of low entropy will as a rule be nearly

orthogonal, two vectors of high entropy, being more uniform, will tend to have substantial overlap.

3 Sources of RM-FM discrepancies

For most model instances there is a great deal of similarity between the steady states observed in the FM and those found in the RM. At high strength variabilities discrepancies between the NEECs of RM and FM instances arise. However, even when the RM-FM discrepancy is large, the islands of circulation in the optimal ECGs include a large majority of the chains on which activity is concentrated, in both RM and FM versions.

The main source of discrepancy between the FM and the RM is in the equilibrium number of waves. This discrepancy is probably due to the approximations involved in the functions defining the RM which neglect the effect of chain strength variability on the stochastic spiking rate and on the probability of pulse packet transmission. Setting the conductance values of all excitatory synaptic noise events to a single value ($g_{\mathrm{E}} = G_\mu$) is an oversimplification, since the strengths of the input synapses of a neuron will follow a distribution approximately the same as that of the chain strengths, $N(G_\mu, G_\sigma)$.

This simplification impacts on the mean number of waves in two ways. Firstly, the stochastic spiking rate $\nu_{\mathrm{S}}(\lambda_{\mathrm{E}})$ is increasingly underestimated in the RM model as chain strength variability increases. This underestimate is because increased variability in synaptic strengths increases the spike rate response to balanced stochastic input (Amit & Brunel, 1997). If the mean spiking rate is unchanged, the higher-than-predicted rate of stochastic spikes will leave less spikes available to participate in synfire waves and thus the mean number of waves will be less. This seems to be the main effect at low strength variabilities because the mean spiking rate in the FM does not deviate much from that estimated from the mean number of waves in the RM using the relation $\nu = \lambda_{\mathrm{E}}(h)/C_{\mathrm{E}}$ (results not shown). Secondly, at higher strength variabilities the mean spiking rate of the FM *does* decrease relative to that of the RM, resulting in a further reduction in the mean number of waves. This means that wave propagation must be less robust to background input when strength variability is taken into account. Thus the RM significantly overestimates $\lambda_{\mathrm{E,th}}(g_{\mathrm{E}})$ at higher chain strength variabilities.

In principle it would be straightforward although computationally intensive to overcome these shortcomings via simulations of synfire chains to compute wave traversal probability as a function not just of chain strength and background input rate, but of strength variability of background input synapses as well. Likewise the stochastic spiking rate function could be extended to take synaptic strength variability into account. With such an improved RM-FM correspondence, we would expect to obtain a similar downward trend in number of waves with strength variability in the RM as well.

The low mean number of waves in the FM at high strength variability could be remedied by reducing the effect of noise feedback on chain traversals and stochastic spiking. This can be achieved by reducing the number of pools in two ways: (a) reducing the total connectivity (to reduce the amount of noise feedback and stochastic spiking); and (b) having bigger but fewer pools for a

given amount of connectivity, in order to increase the robustness of chains.

References

Amit, D. J. & Brunel, N. (1997). Model of global spontaneous activity and local structured activity during delay periods in the cerebral cortex. *Cerebral Cortex*, *7*, 237–252.

Trengove, C., van Leeuwen, C., & Diesmann, M. (2013). High capacity embedding of synfire chains in a cortical network model. *Journal of Computational Neuroscience*, *34*(2), 185–209.
