# Supplementary material for: Dynamic effective connectivity in cortically embedded systems of recurrently coupled synfire chains
Source: J Comput Neurosci. 2015 Nov 11;40:1–26. doi: 10.1007/s10827-015-0581-5 (PMC4762935; doi:10.1007/s10827-015-0581-5)
Supplement: Supplementary file 2 — (PDF 4.89 MB) [file 10827_2015_581_MOESM2_ESM.pdf]

Online Resource 2: Supplementary Figures

for Journal of Computational Neuroscience article Dynamic effective connectivity in cortically embedded systems of recurrently coupled synfire chains

Chris Trengove[1], Markus Diesmann, Cees van Leeuwen

Supplementary Figure captions

Here are included supporting figures for the durations of ongoing activity (Supplementary Figure 1), the EE rasters of selected runs and their pairwise distances for selected model instances (Supplementary Figures 2, 3, 4, 5, 6, 7, 8), the contributions of the principal components to NEEC variance (Supplementary Figures 9, 10), a figure showing all RM-RM and RM-FM distance sets (Supplementary Figure 11), RM-FM Discrepancies (Supplementary Figure 12), and for selected model instances figures showing the ECGs with EE rasters colored and sorted according to condensed ECG parts (Supplementary Figures 13, 14, 15, 16, 17).

Supplementary Figure 1: Transient activity runs in the RM model (left column) and the FM model (right column). The heights of the vertical bars in the top row give the fraction of runs in which activity was transient or unstable activity. The bar is broken up into sub-bars for the fraction of runs with (i) transient activity of duration less than 10000 ms (blue), (ii) transient activity of duration more than 10000 ms (green), (iii) unstable activity (red). In the bottom row the bar heights give the mean (red) and the maximum (blue) durations of activity for runs in which activity is transient. The horizontal axis is the extended RMP index, 10 $\times G_\sigma$-index + RMP-index.

Supplementary Figure 2: Behavior at zero strength variability. RM & FM EE activity for RMP 4, $G_\sigma/G_\mu = 0$. Top left panel: 2PC projection of NEECs of RM (blue) and FM (red) runs. Three RM runs (green, labels a, b, c) and three FM runs (labels A, B, C) are selected. Top right panel: matrix of distances between these six NEECs. Remaining panels: EE rasters for RM runs 'a'-'c' (left column) and FM runs 'A'-'C' (right column). Note the uniformity of activity across runs, the unstructured 2PC projection with small component magnitudes, the moderate RM-RM distances and slightly higher RM-FM distances.

Supplementary Figure 3: RM EE activity for RMP 1, $G_\sigma/G_\mu = 0.25$. Top left panel: 2PC projection of NEECs. Two RM runs are selected (green, labels 'a', 'b'). Top right panel: matrix of pairwise distances NEECs. Lower panels: EE rasters for RM runs 'a', 'b'. The 1st PC distinguishes two clusters. In run 'a' is one of 4 runs making up the right cluster for which the initially stimulated chain is self looping and activity is confined to this chain for the entire duration

[1]Corresponding author, email: trengove.c@gmail.com

of the run. Run ‘b’ is one of the remaining runs in the left cluster. Excluding the runs in the right cluster before doing PCA allows the left cluster to be better resolved.

Supplementary Figure 4: An example of high RM-FM discrepancy. RM & FM EE activity over runs for RMP 4, $G_\sigma/G_\mu = 0.35$. Top left panel: 2PC projection of NEECs of RM (blue) and FM (red) runs. One RM run (green, labels a, b) and two FM runs (labels A, B) are selected. Top right panel: matrix of distances between these 4 NEECs. Remaining panels: EE rasters for RM runs ‘a’, ‘b’ (left column) and FM runs ‘A’, ‘B’ (right column). Activity in the FM runs largely occurs on the same chains that it occurs on in the RM runs. The large RM-FM distances are due to a strong reduction in the FM runs of activity on certain chains which are highly active if the RM runs, notably in the case of FM run ‘B’. Hence the FM runs show an increase in entropy relative to the RM runs.

Supplementary Figure 5: RM EE activity for RMP 5, $G_\sigma/G_\mu = 0.2$. 2PC projection of all NEECs (top left); for eight RM runs (‘a’ through ‘h’, green circles in 2PC projection plot) their NEEC distance matrix (top right) and EE raster plots (lower panels). The first PC reflects the relative amounts of time spent in two almost orthogonal steady states, exemplified by runs ‘c’ and ‘f’ respectively. In run ‘d’ a superposition of these two states holds sway between 80 and 85 seconds into the run, while in run ‘e’ a superposition is present between 75 and 125 seconds into the run. The second PC measures the activity of a self-looping chain (id 636): both the amount of run time for which it is active and the number of waves simultaneously present on it. In runs ‘b’ and ‘h’ a single wave traverses this chain for the entire run duration. In runs ‘e’ and ‘g’ a single wave appears on this chain about midway through the run. In run ‘a’ *two* waves traverse the chain simultaneously.

Supplementary Figure 6: RM EE activity over runs for RMP 6, $G_\sigma/G_\mu = 0.35$. Top left panel: 2PC projection of NEECs. Four RM runs are selected (green, labels a-d). Top right panel: matrix of pairwise distances NEECs. Remaining panels: EE rasters for runs ‘a’-‘d’. Runs ‘a’-‘c’ exhibit bidirectional transitions between two steady states. Their positions along the major axis of the elongated lower cluster reflect the relative amounts time spend in these two states. Run ’d’ is one of a small cluster that exhibit a third steady state.

Supplementary Figure 7: RM EE activity over runs for RMP 8, $G_\sigma/G_\mu = 0.15$. Top left panel: 2PC projection of NEECs. Six RM runs are selected (green, labels a-f). FM runs in red. Top right panel: matrix of pairwise distances NEECs. Remaining panels: EE rasters for runs ‘a’-‘f’. Comparing the EE raster of run ‘a’ (in the main cluster) to that of ‘f’ (in the smaller cluster) we see that run ‘f’ has strong activity throughout the run duration on three chains that are silent in run ‘a’. Intermediately positioned NEECs of runs ‘b’ through ‘e’ vary according to the time at which activity appeared on these three chains.

Supplementary Figure 8: RM EE activity over runs for RMP 9, $G_\sigma/G_\mu = 0.3$. Top left panel: 2PC projection of NEECs. Four RM runs are selected

(green, labels a-d). FM runs in red. Top right panel: matrix of pairwise distances NEECs. Remaining panels: EE rasters for runs 'a'-'d'. All runs exhibit a single steady state of high entropy.

Supplementary Figure 9: Variance contributions of the first 20 PCs for each RMP. Colors from black to copper correspond to chain strength variabilities $G_\sigma/G_\mu = 0$, 0.05, 0.1, ..., 0.4. As chain strength variability increases, the first few variance components increase while the later ones decrease.

Supplementary Figure 10: Proportion of total variance contributed by the first k PCs versus chain strength variability, $k \in \{1, \dots, 10\}$.

Supplementary Figure 11: Distances $d(\mathcal{D}^\sigma, \mathcal{D}^\rho)$ between target vectors of model σ and the vector set of RM model $\rho = (\alpha, \gamma, \mathrm{RM})$ for: (blue) $\sigma = \rho$ (the RM set itself); (red) $\sigma = (\alpha, \gamma, \mathrm{FM})$ (the corresponding FM set); (green) $\sigma = (\alpha', \gamma, \mathrm{FM}), \alpha' \neq \alpha$ (non-corresponding FM data sets). Odd-numbered columns: $d = d_{\mathrm{min}}$. Even-numbered columns: $d = d_{\mathrm{2PC}}$. Chain strength variability in indexed by γ: $G_\sigma/G_\mu = 0.05\gamma$.

Supplementary Figure 12: RM-FM Discrepancies (based on RM-RM and FM-RM NEEC distance sets) versus G_σ/G_μ for RMPs $0, \dots, 9$. Distance sets using $d = d_{\mathrm{min}}$ (left panel), $d = d_{\mathrm{2PC}}$ (right panel). Error bars: ± 1 standard deviation.

Supplementary Figure 13: Condensed ECG (cECG), 2PC projection, and selected EE rasters colored and sorted according to cECG for RMP 2, $G_\sigma/G_\mu = 0.3$. The first PC reflects the relative proportion of time in the 'a' state and the 'c' state, driven primarily by SC 4 and SC 0 respectively. The 'c' state also includes intermittent recurrent activation of SC 1 via SC 2 or both SC 3 and SC 2. The second PC primarily reflects the additional presence of one or two co-active waves on SC 5 (a self-looping chain).

Supplementary Figure 14: Optimal condensed ECG (cECG), 2PC projection, and selected EE rasters colored and sorted according to cECG for RMP 4, $G_\sigma/G_\mu = 0.4$. The second PC reflects the amount of activity on the SC1, OC1, SC0, OC0 pathway. The first PC reflects the average number of waves present simultaneously on the simple loop SC3 (brown). The discrete sub-clumps that are apparent when activity on the SC1, OC1, SC0, OC0 pathway is low (i.e. when the 2nd PC is low) are due to approximately integral values of the average number of waves on the simple loop SC3. Thus runs 'f', 'g', 'h', 'i' and 'j' have for most of their duration 7, 6, 5, 4 and 3 waves present simultaneously on SC3, respectively.

Supplementary Figure 15: Optimal condensed ECG (cECG), 2PC projection, and selected EE rasters colored and sorted according to cECG for RMP 5, $G_\sigma/G_\mu = 0.2$. The first PC distinguishes two island of circulation driven by SC 0 and SC 1 activity respectively. The second PC distinguishes the additional presence of one or two co-active waves on SC 2 (a self-looping chain).

Supplementary Figure 16: Optimal condensed ECG (cECG), 2PC projection, and selected EE rasters colored and sorted according to cECG for RMP 6, $G_\sigma/G_\mu = 0.35$. The predominant pattern of activity involves bidirectional transitions between a low entropy state driven by SC 0 and a high entropy state driven by SC 2 (runs 'a', 'b' and 'c'). A more rarely observed steady state is driven by activity on SC 1.

Supplementary Figure 17: Optimal condensed ECG (cECG), 2PC projection, and selected EE rasters colored and sorted according to cECG for RMP 9, $G_\sigma/G_\mu = 0.3$. A single high-entropy steady state is observed, consisting primarily of activity on SC 0, SC 2, and their respective out-components, excluding SC 1 and the remainder of its outcomponent which are only intermittently and briefly activated. The intermittent activation on SC 0 and SC 2 shows that on-going activity also relies on weaker chains which are excluded from this ECG (coloured black in raster plot).

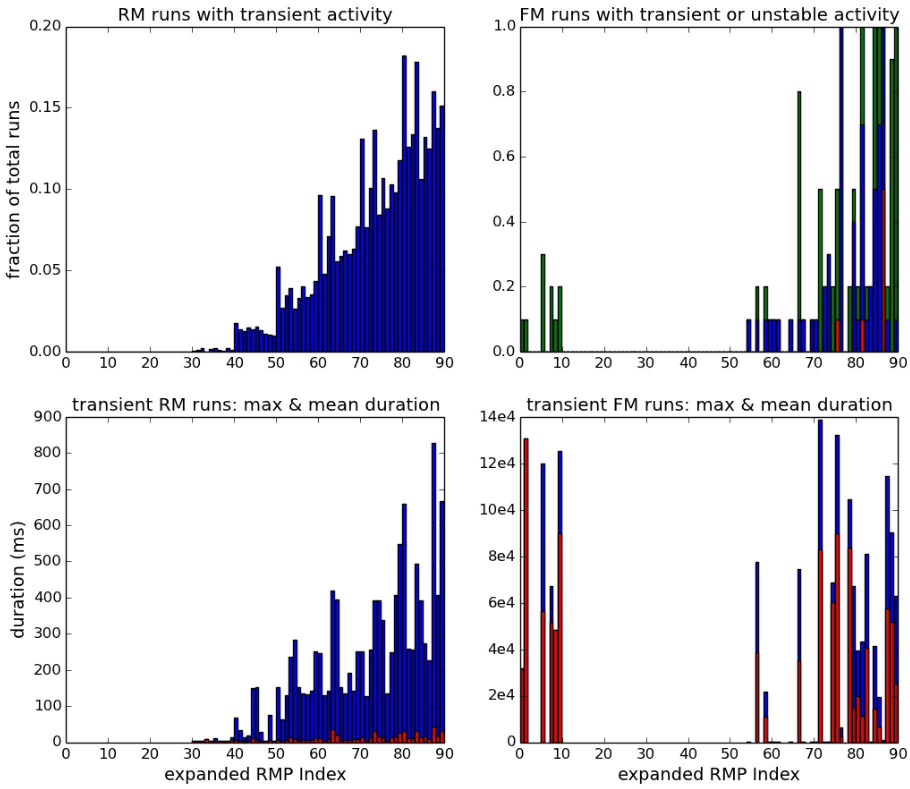

Figure 1: Transient activity runs in the RM and FM models.

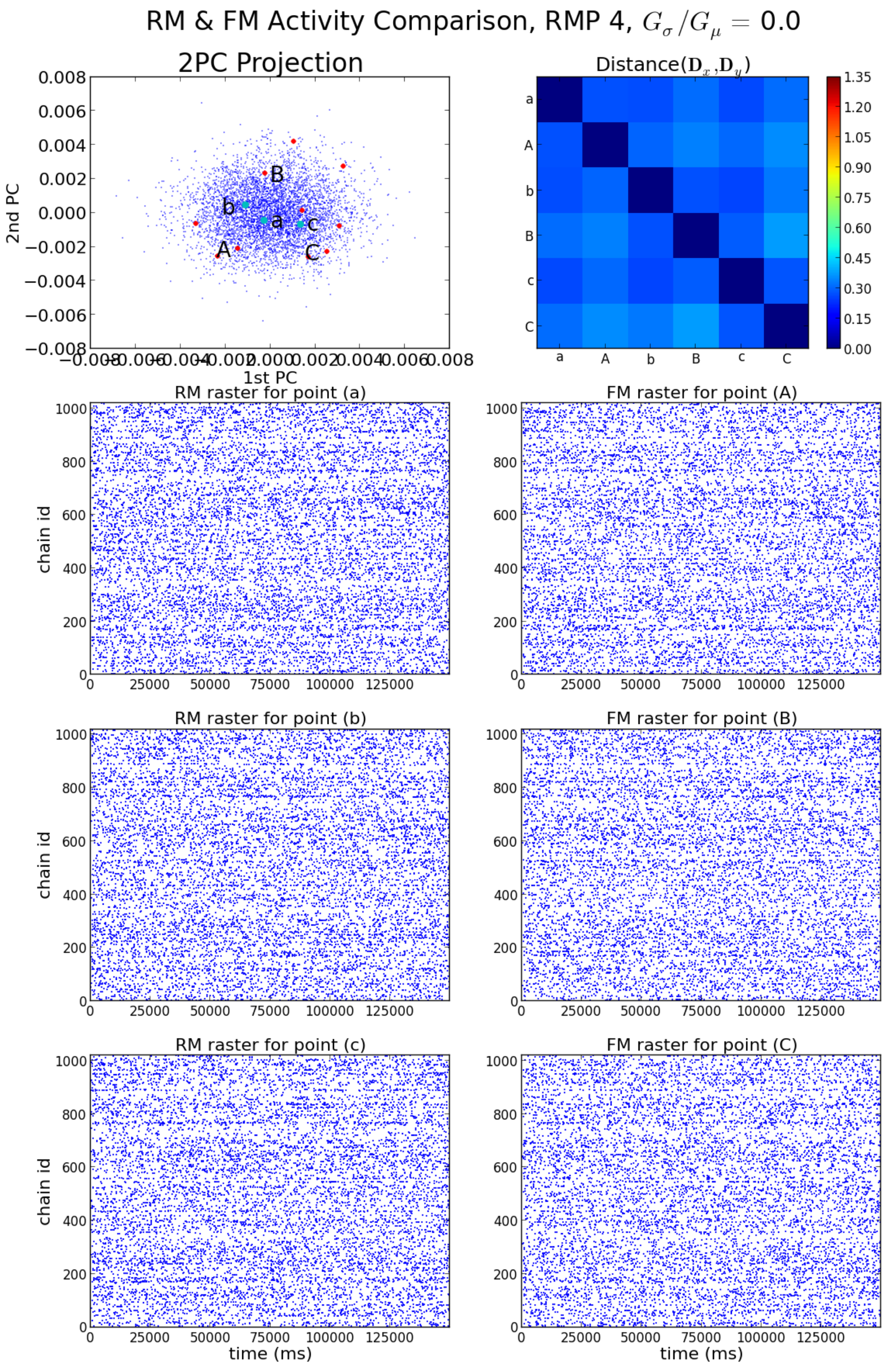
RM & FM Activity Comparison, RMP 4, $G_\sigma/G_\mu = 0.0$
2PC Projection
2nd PC
1st PC
Distance($\mathbf{D}_x$,$\mathbf{D}_y$)
RM raster for point (a)
FM raster for point (A)
RM raster for point (b)
FM raster for point (B)
RM raster for point (c)
FM raster for point (C)
chain id
time (ms)

Figure 2

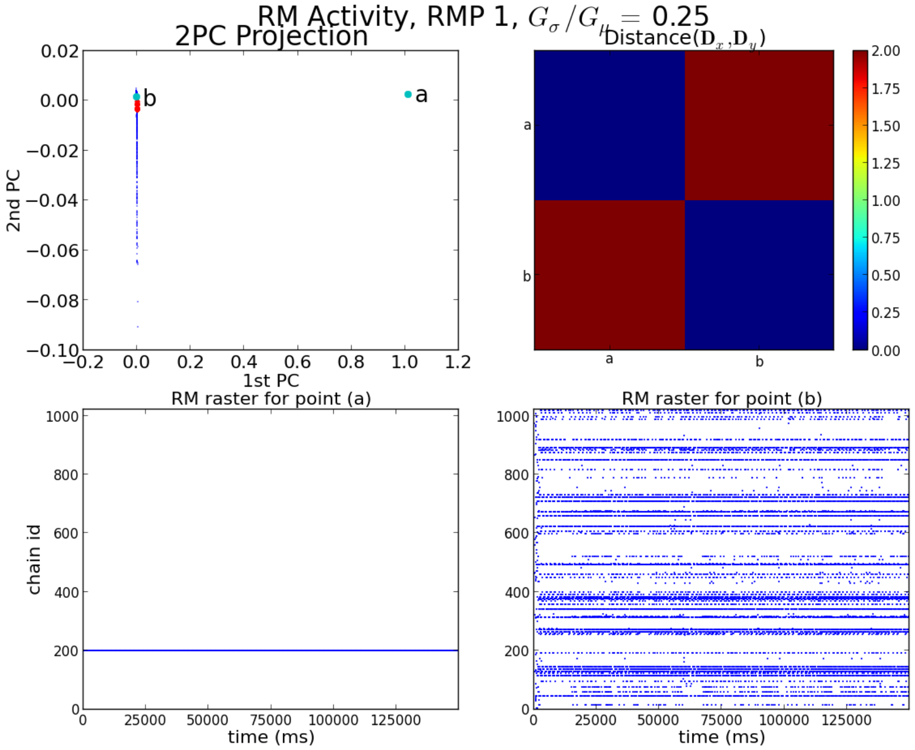

RM Activity, RMP 1, $G_\sigma/G_\mu = 0.25$
2PC Projection
2nd PC
1st PC
a
b
Distance($\mathbf{D}_x$,$\mathbf{D}_y$)
RM raster for point (a)
RM raster for point (b)
chain id
time (ms)

Figure 3

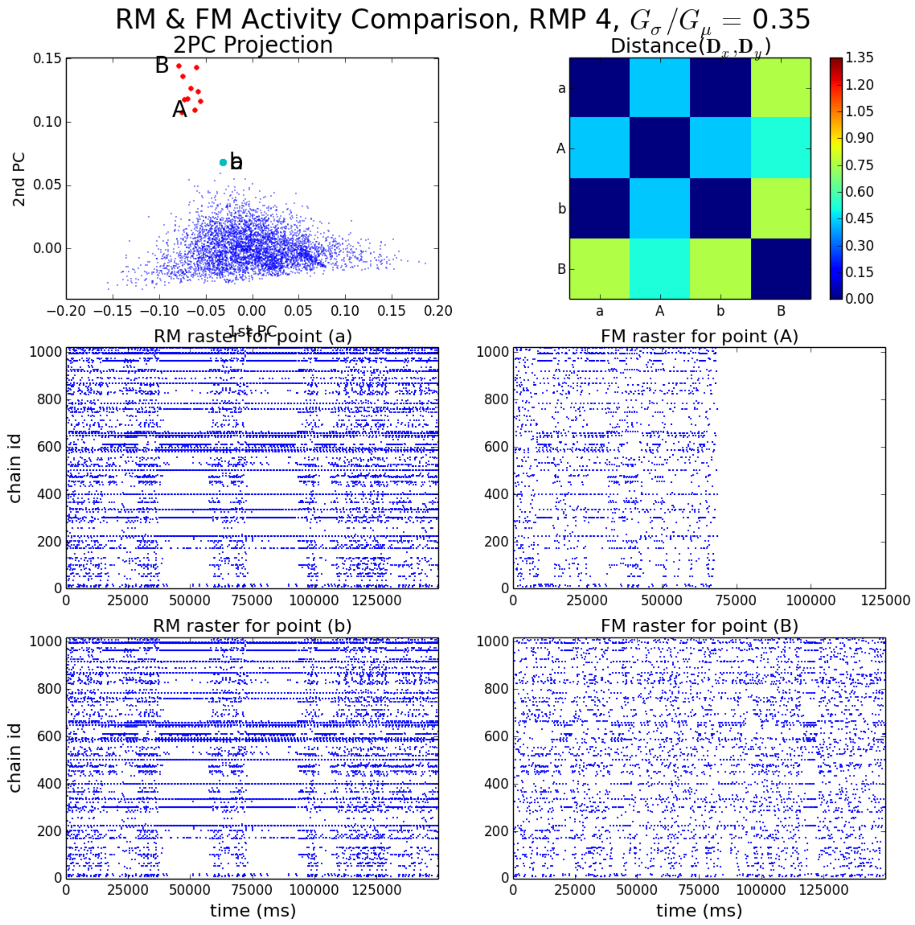
RM & FM Activity Comparison, RMP 4, $G_\sigma/G_\mu = 0.35$
2PC Projection
1st PC
2nd PC
Distance($\mathbf{D}_x, \mathbf{D}_y$)
RM raster for point (a)
FM raster for point (A)
RM raster for point (b)
FM raster for point (B)
chain id
time (ms)

Figure 4

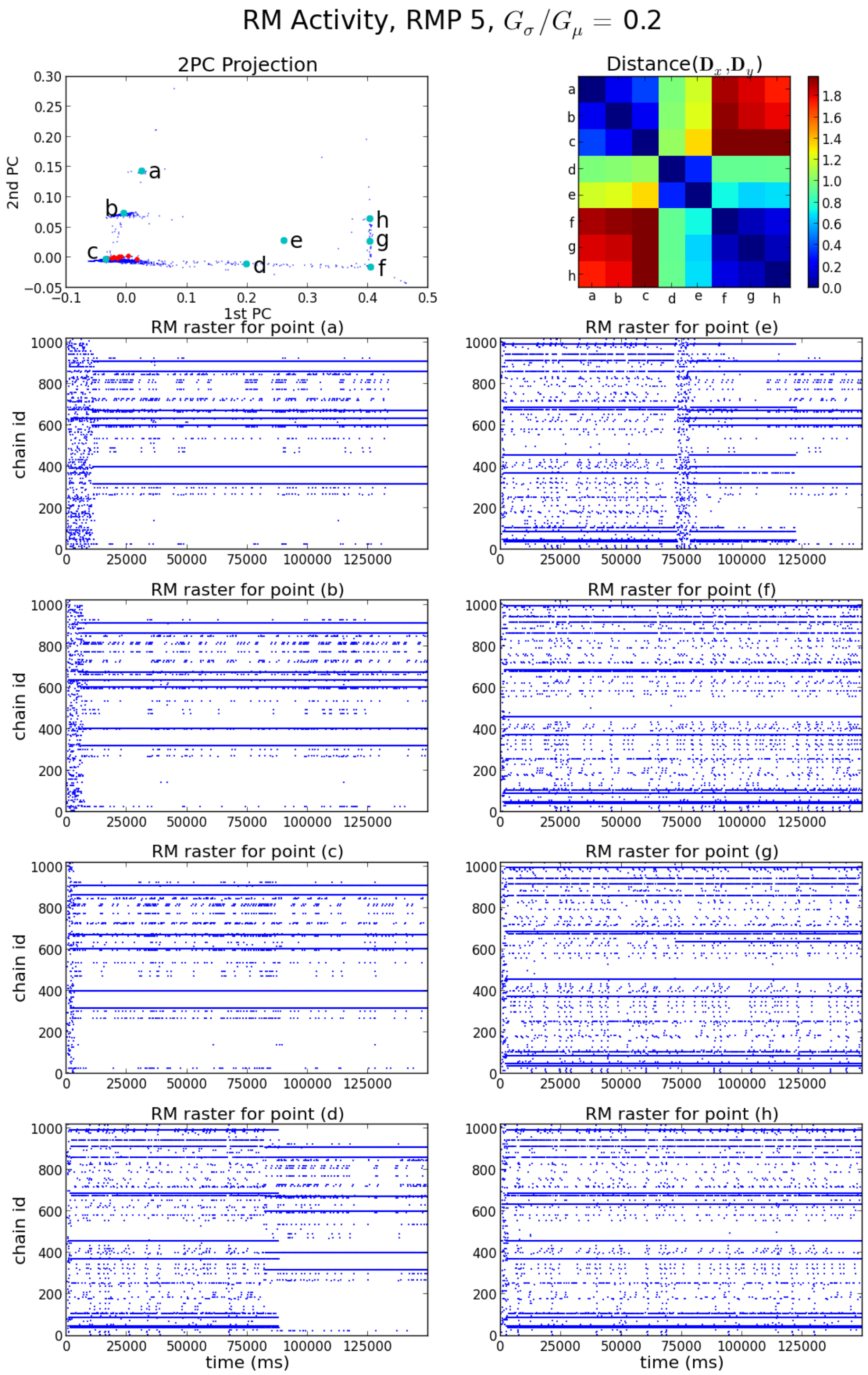
RM Activity, RMP 5, $G_\sigma/G_\mu = 0.2$
2PC Projection
2nd PC
1st PC
Distance($\mathbf{D}_x$,$\mathbf{D}_y$)
RM raster for point (a)
RM raster for point (b)
RM raster for point (c)
RM raster for point (d)
RM raster for point (e)
RM raster for point (f)
RM raster for point (g)
RM raster for point (h)
chain id
time (ms)

Figure 5

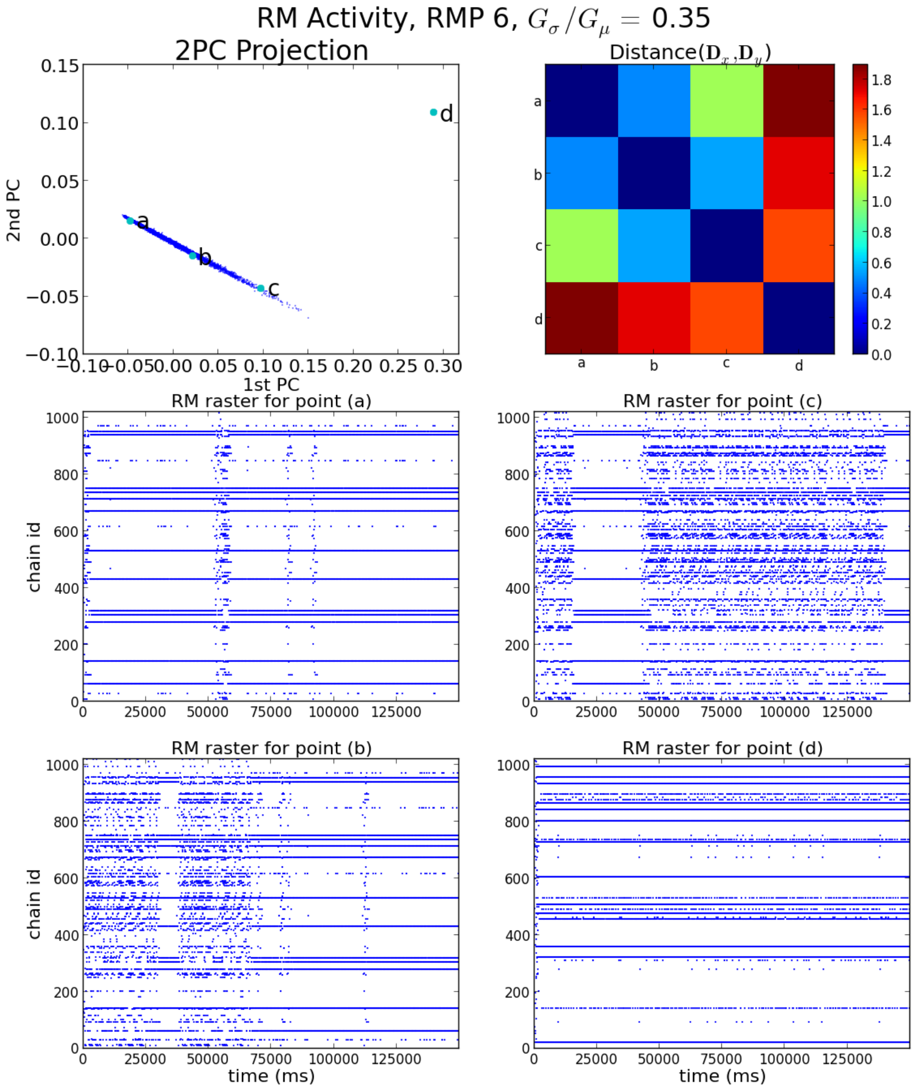
RM Activity, RMP 6, $G_\sigma/G_\mu = 0.35$
2PC Projection
Distance($\mathbf{D}_x$,$\mathbf{D}_y$)
1st PC
2nd PC
RM raster for point (a)
RM raster for point (c)
RM raster for point (b)
RM raster for point (d)
chain id
time (ms)

Figure 6

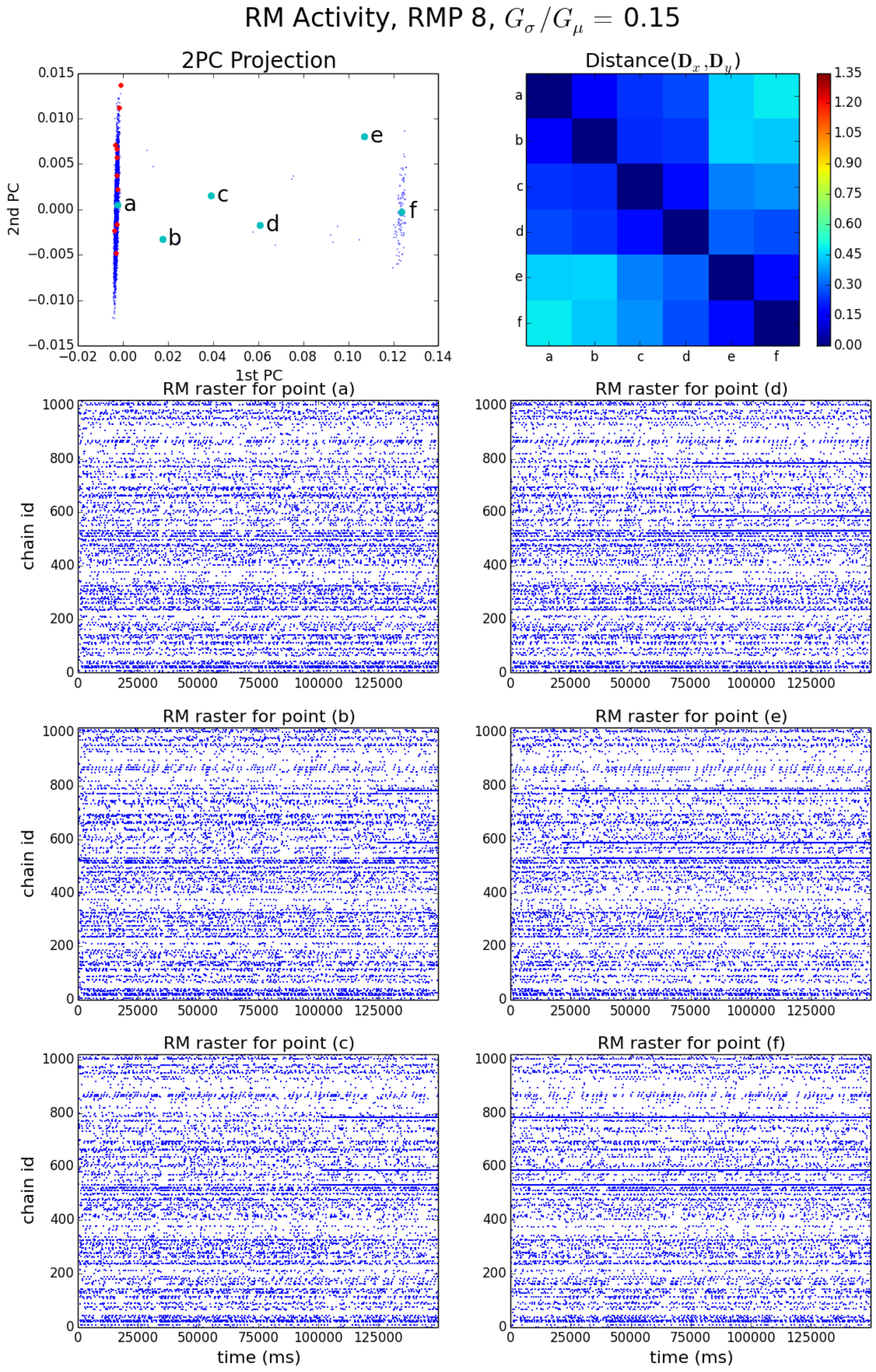
RM Activity, RMP 8, G_σ/G_μ = 0.15
2PC Projection
2nd PC
1st PC
Distance($\mathbf{D}_x$,$\mathbf{D}_y$)
RM raster for point (a)
RM raster for point (b)
RM raster for point (c)
RM raster for point (d)
RM raster for point (e)
RM raster for point (f)
chain id
time (ms)

Figure 7

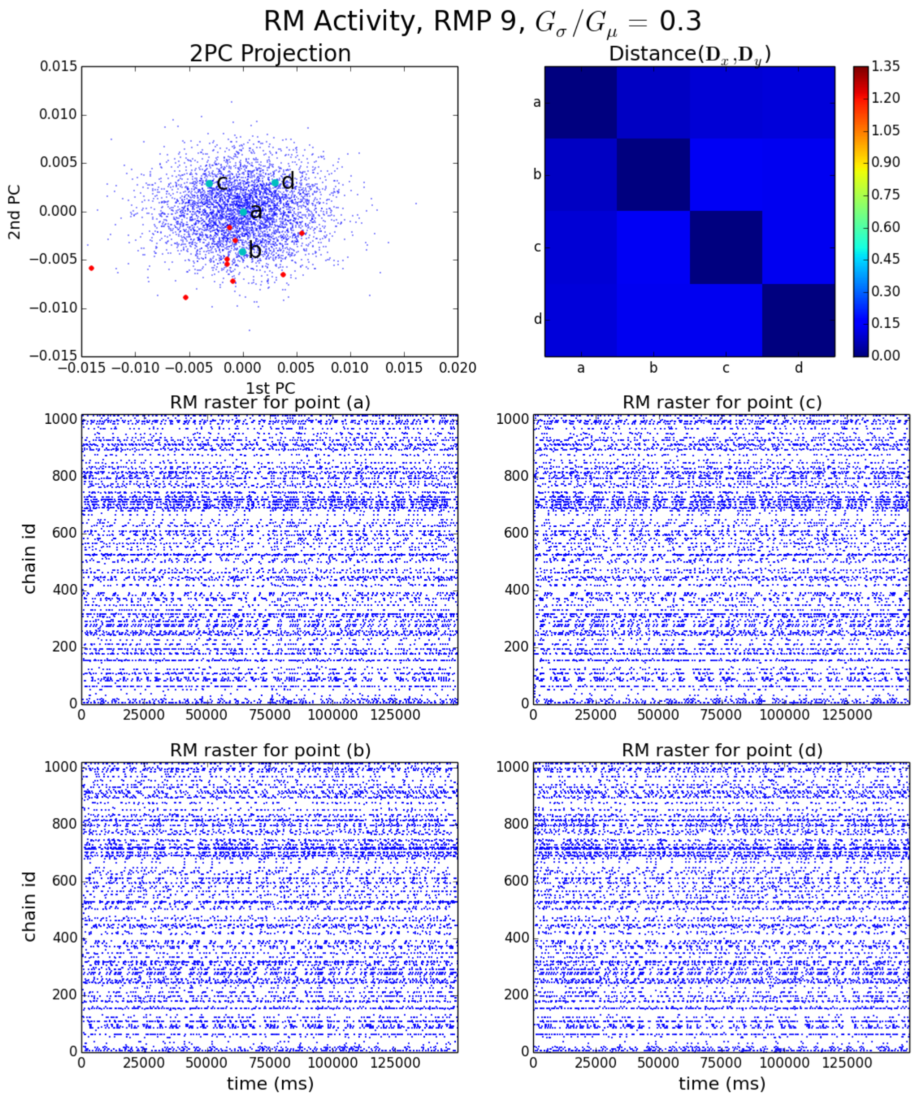
RM Activity, RMP 9, G_σ/G_μ = 0.3
2PC Projection
2nd PC
1st PC
Distance($\mathbf{D}_x$,$\mathbf{D}_y$)
RM raster for point (a)
RM raster for point (c)
RM raster for point (b)
RM raster for point (d)
chain id
time (ms)

Figure 8

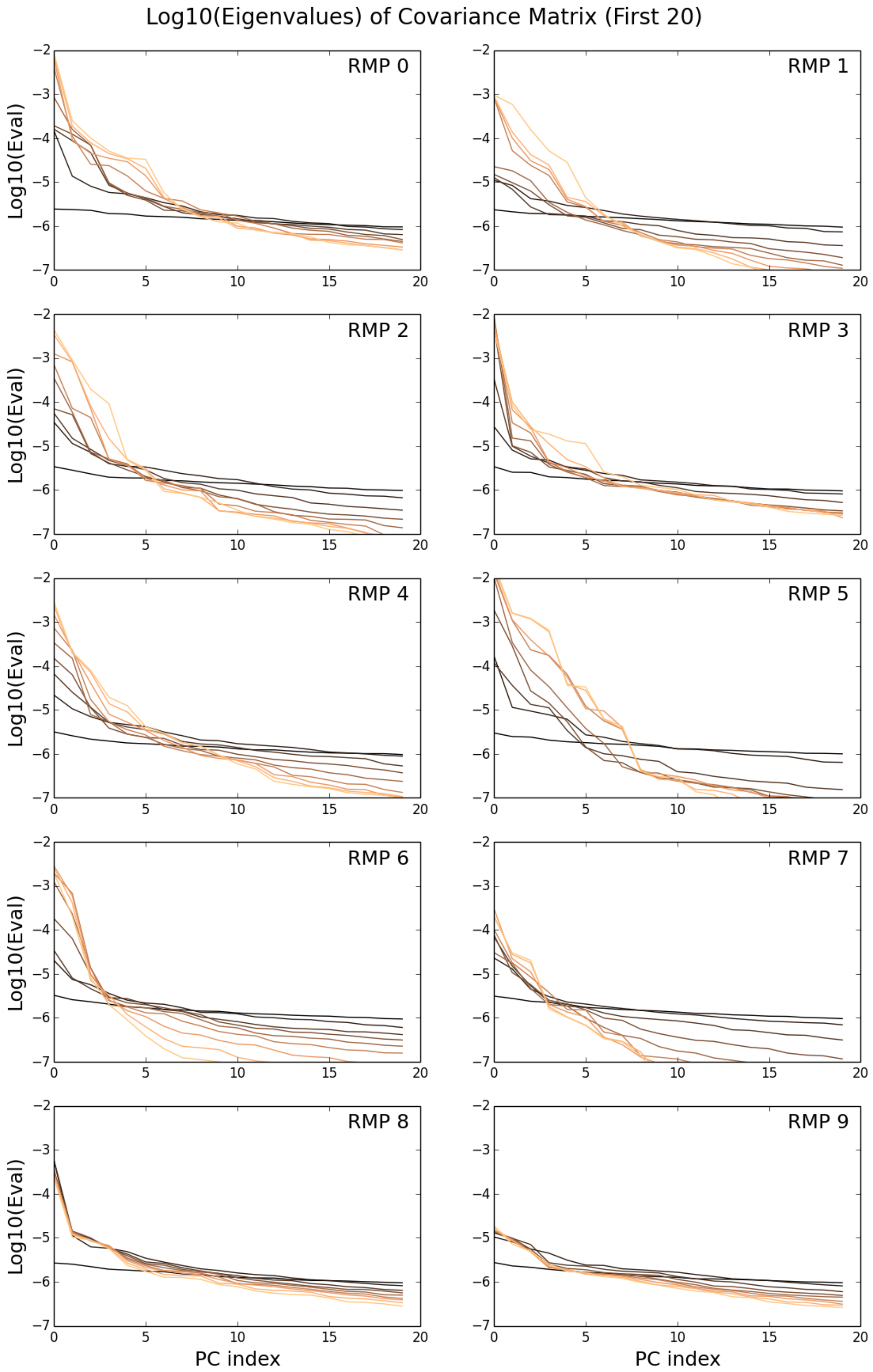

Log10(Eigenvalues) of Covariance Matrix (First 20)
RMP 0
RMP 1
RMP 2
RMP 3
RMP 4
RMP 5
RMP 6
RMP 7
RMP 8
RMP 9
Log10(Eval)
PC index

Figure 9

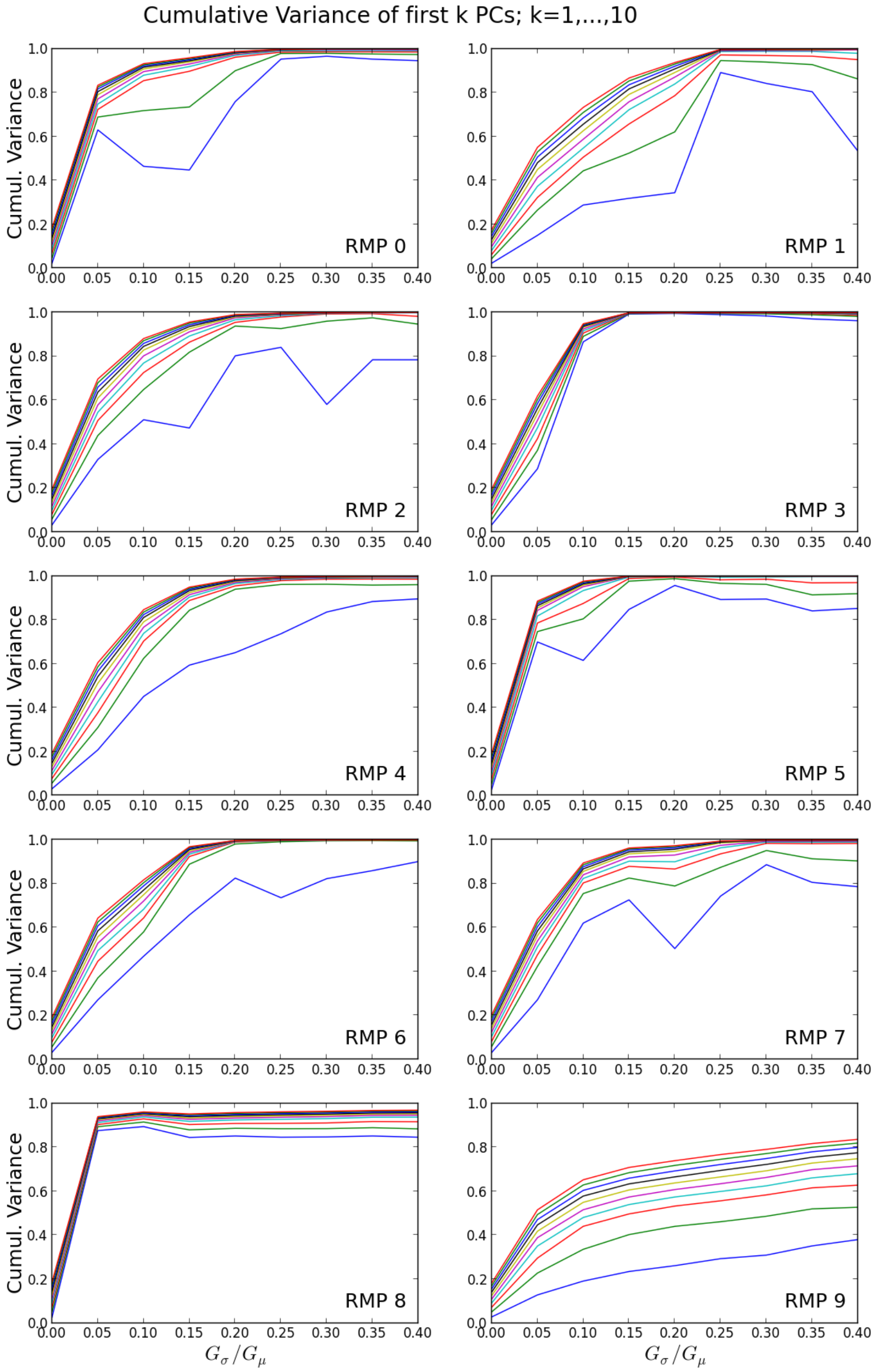
Cumulative Variance of first k PCs; k=1,...,10
Cumul. Variance
RMP 0
RMP 1
RMP 2
RMP 3
RMP 4
RMP 5
RMP 6
RMP 7
RMP 8
RMP 9
G_σ/G_μ

Figure 10

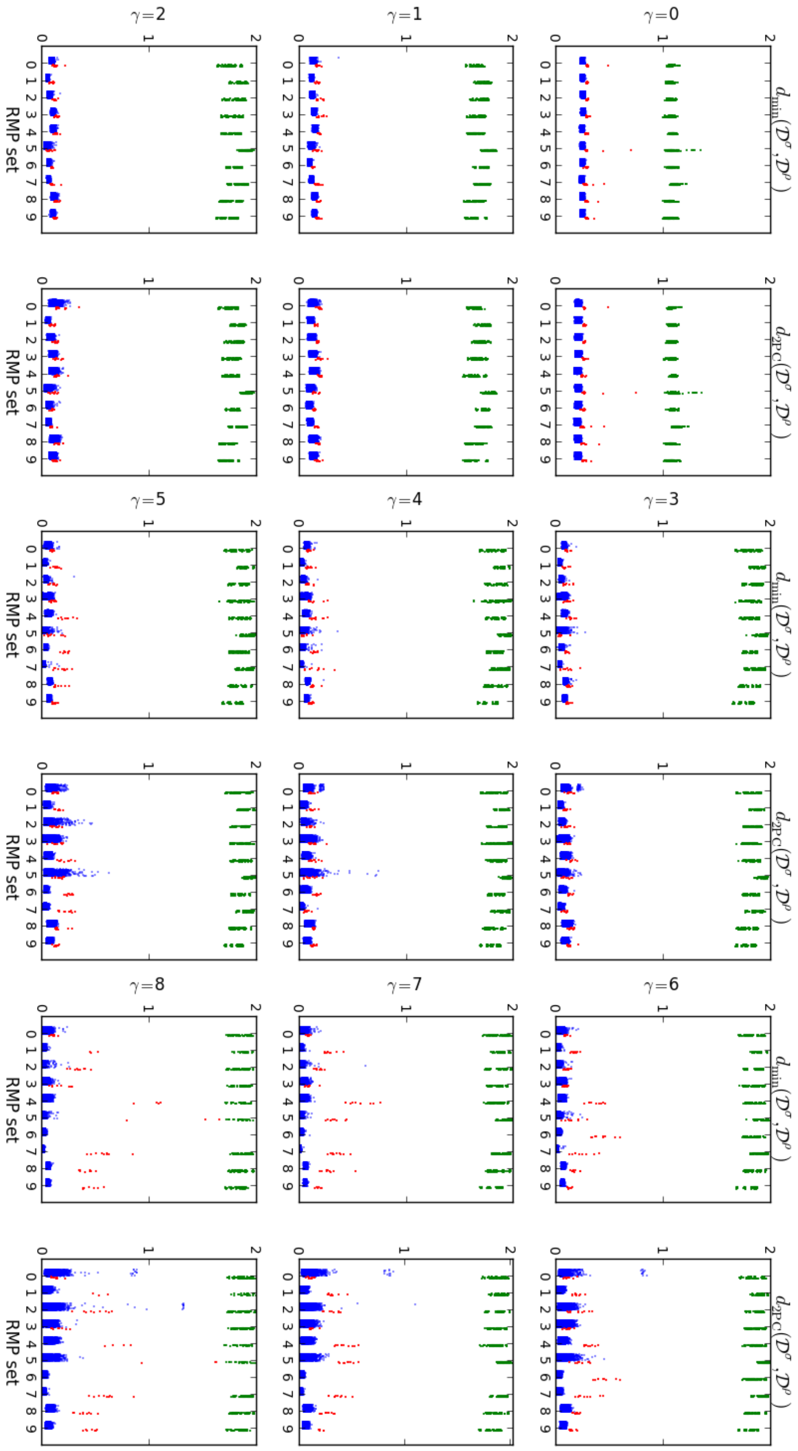

Figure 11

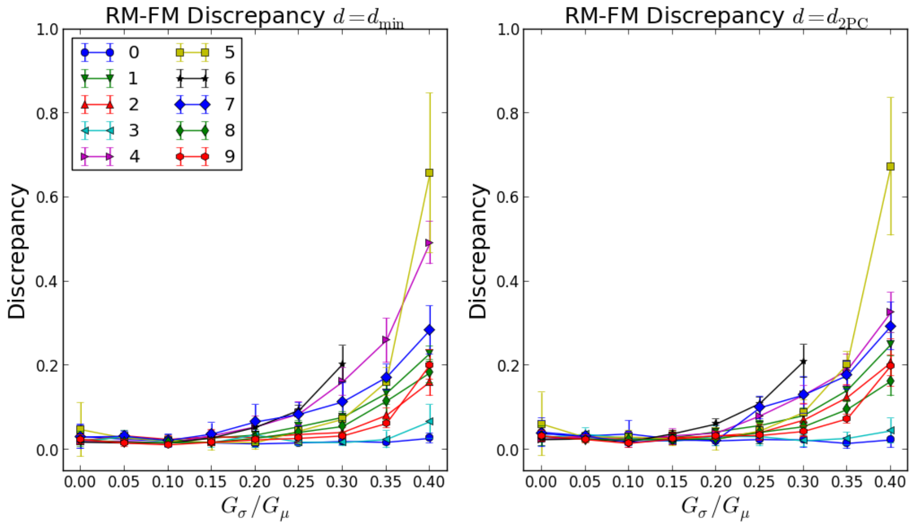

RM-FM Discrepancy $d=d_{\min}$
RM-FM Discrepancy $d=d_{2PC}$
Discrepancy
G_σ/G_μ
0
1
2
3
4
5
6
7
8
9

Figure 12

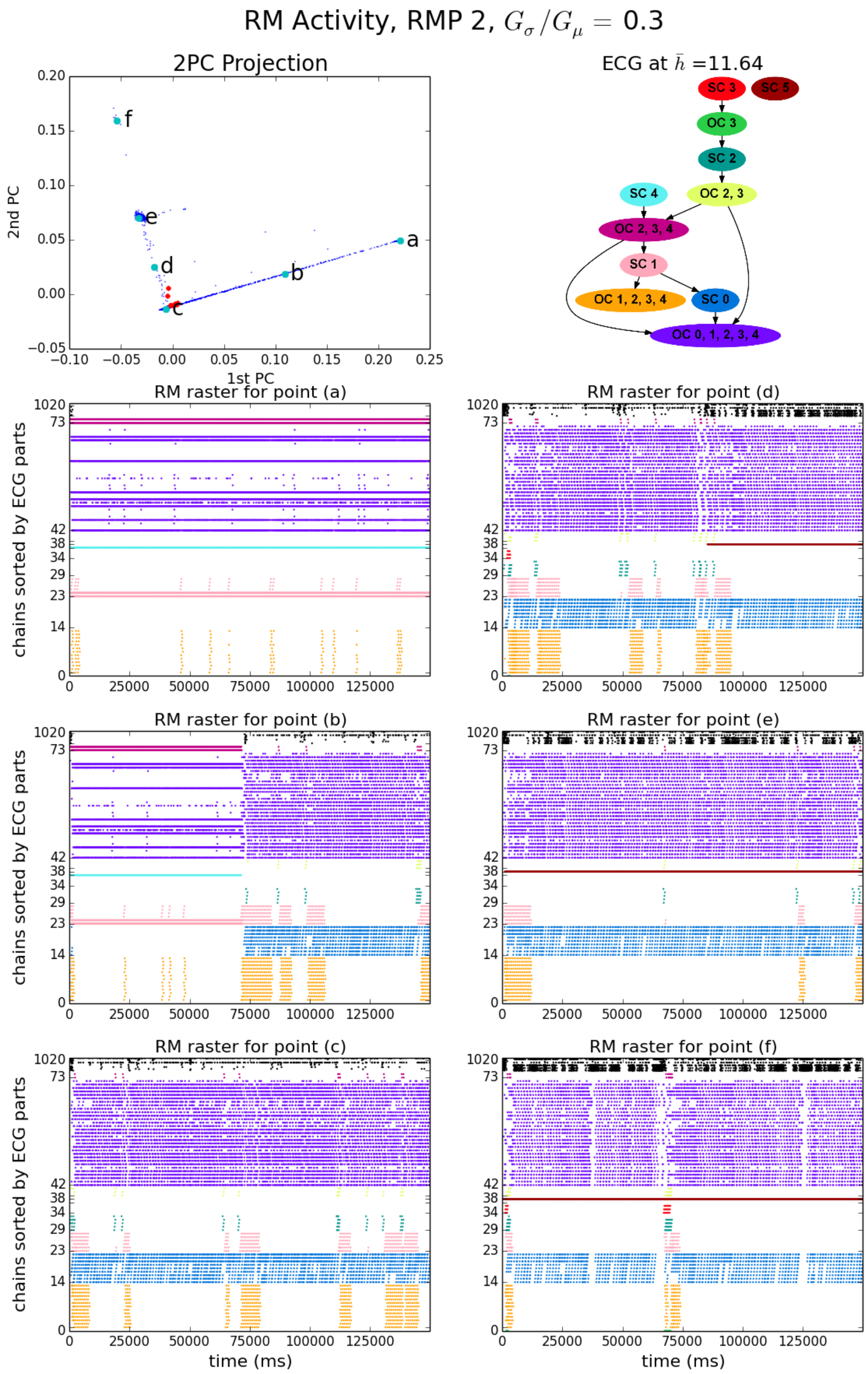
RM Activity, RMP 2, G_σ/G_μ = 0.3
2PC Projection
1st PC
2nd PC
a
b
c
d
e
f
ECG at $\bar{h}$ =11.64
SC 3
SC 5
OC 3
SC 2
SC 4
OC 2, 3
OC 2, 3, 4
SC 1
OC 1, 2, 3, 4
SC 0
OC 0, 1, 2, 3, 4
RM raster for point (a)
RM raster for point (b)
RM raster for point (c)
RM raster for point (d)
RM raster for point (e)
RM raster for point (f)
chains sorted by ECG parts
time (ms)

Figure 13

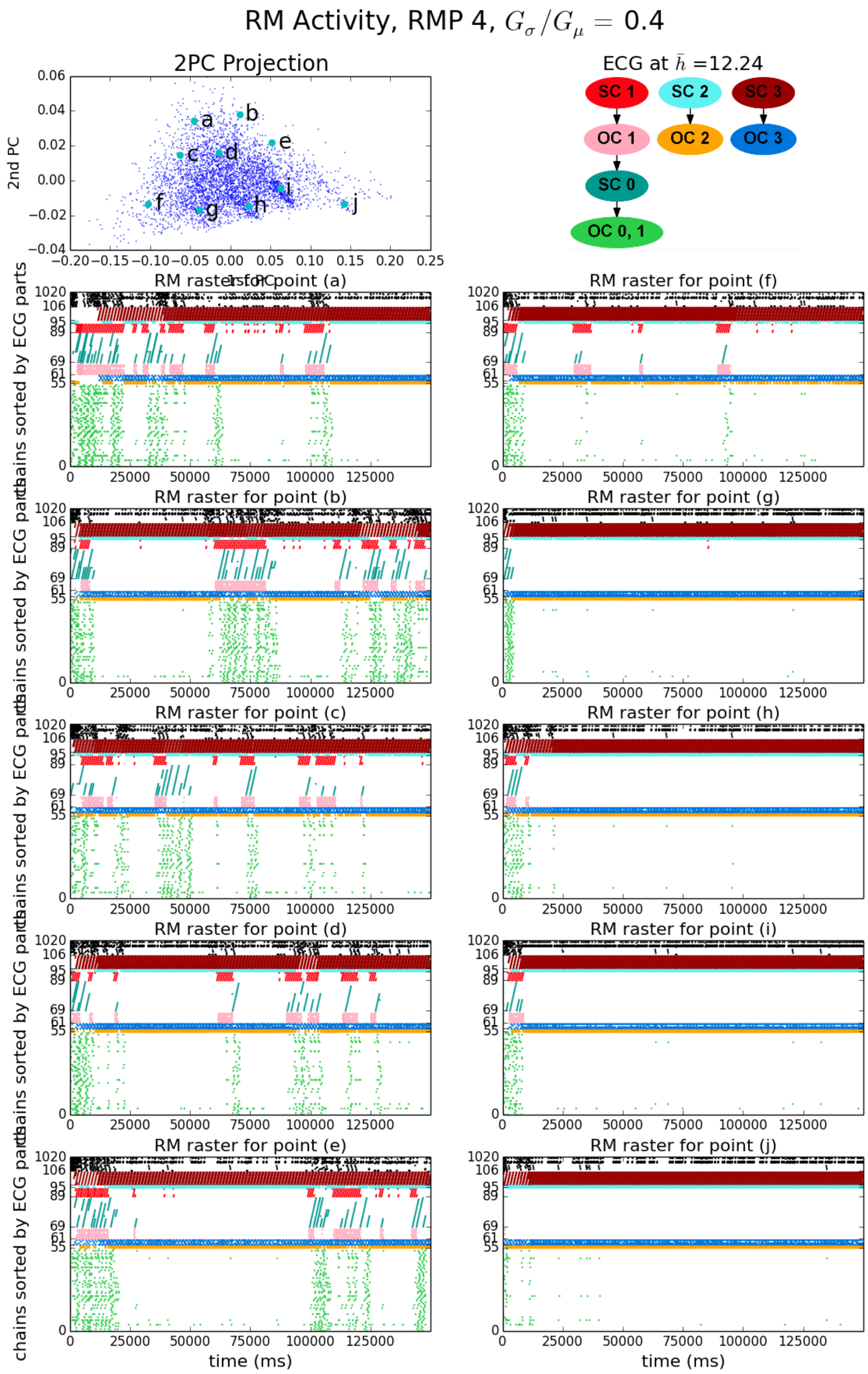
RM Activity, RMP 4, G_σ/G_μ = 0.4
2PC Projection
2nd PC
1st PC
ECG at $\bar{h}$ =12.24
SC 1
SC 2
SC 3
OC 1
OC 2
OC 3
SC 0
OC 0, 1
RM raster for point (a)
RM raster for point (b)
RM raster for point (c)
RM raster for point (d)
RM raster for point (e)
RM raster for point (f)
RM raster for point (g)
RM raster for point (h)
RM raster for point (i)
RM raster for point (j)
chains sorted by ECG parts
time (ms)

Figure 14

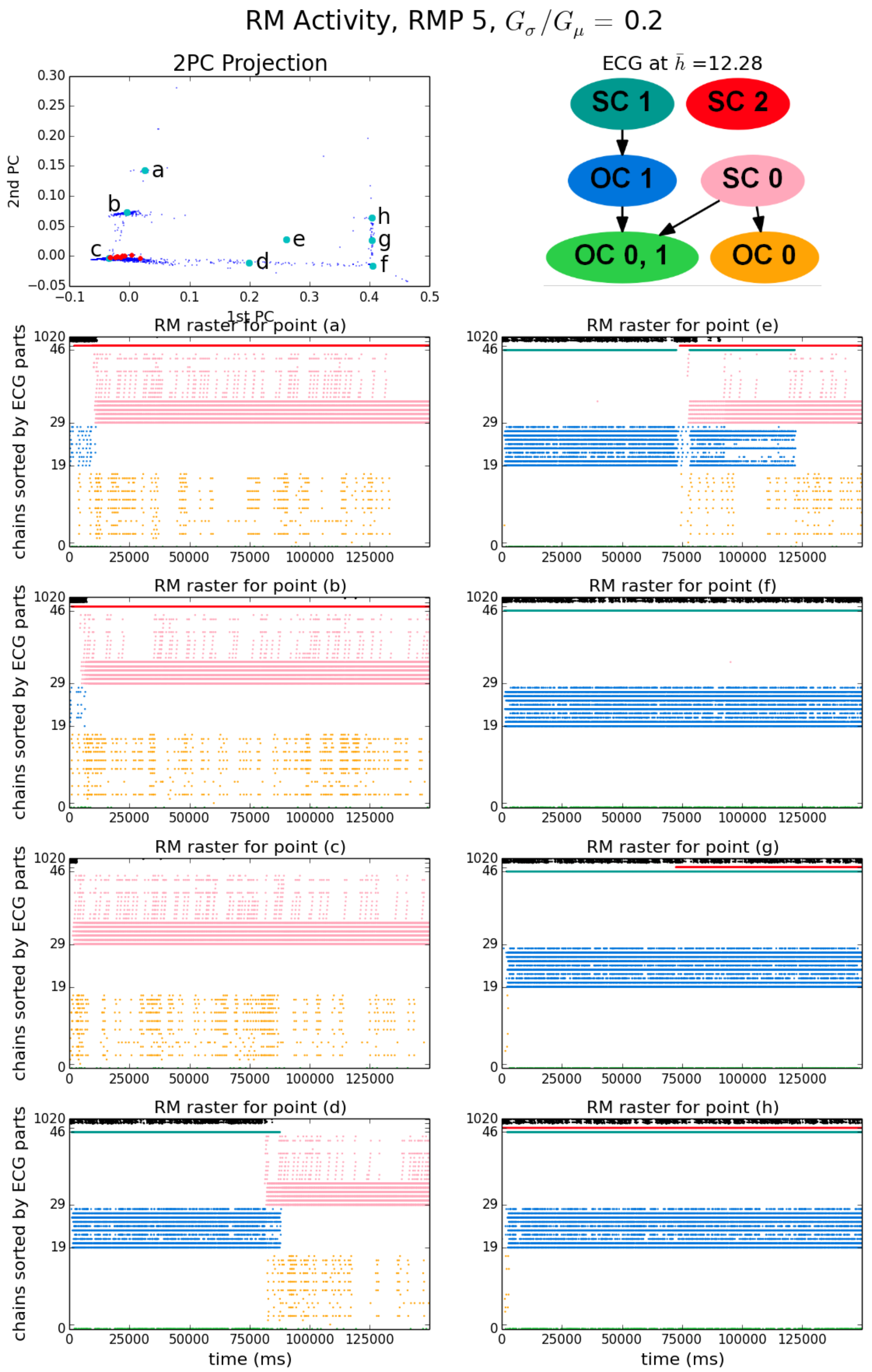
RM Activity, RMP 5, G_σ/G_μ = 0.2
2PC Projection
2nd PC
1st PC
a
b
c
d
e
f
g
h
ECG at $\bar{h}$ =12.28
SC 1
SC 2
OC 1
SC 0
OC 0, 1
OC 0
RM raster for point (a)
RM raster for point (b)
RM raster for point (c)
RM raster for point (d)
RM raster for point (e)
RM raster for point (f)
RM raster for point (g)
RM raster for point (h)
chains sorted by ECG parts
time (ms)

Figure 15

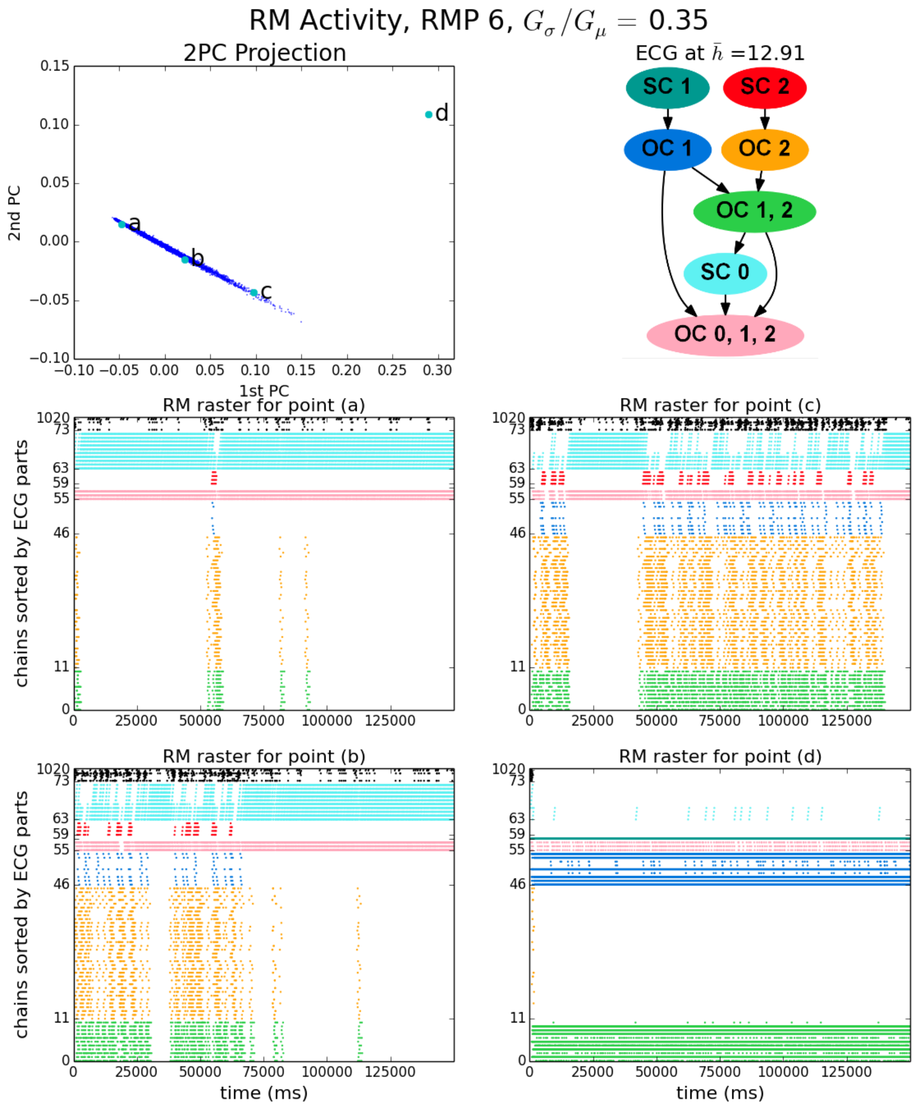
RM Activity, RMP 6, $G_\sigma/G_\mu = 0.35$
2PC Projection
1st PC
2nd PC
a
b
c
d
ECG at $\bar{h}$ =12.91
SC 1
SC 2
OC 1
OC 2
OC 1, 2
SC 0
OC 0, 1, 2
RM raster for point (a)
RM raster for point (c)
RM raster for point (b)
RM raster for point (d)
chains sorted by ECG parts
time (ms)

Figure 16

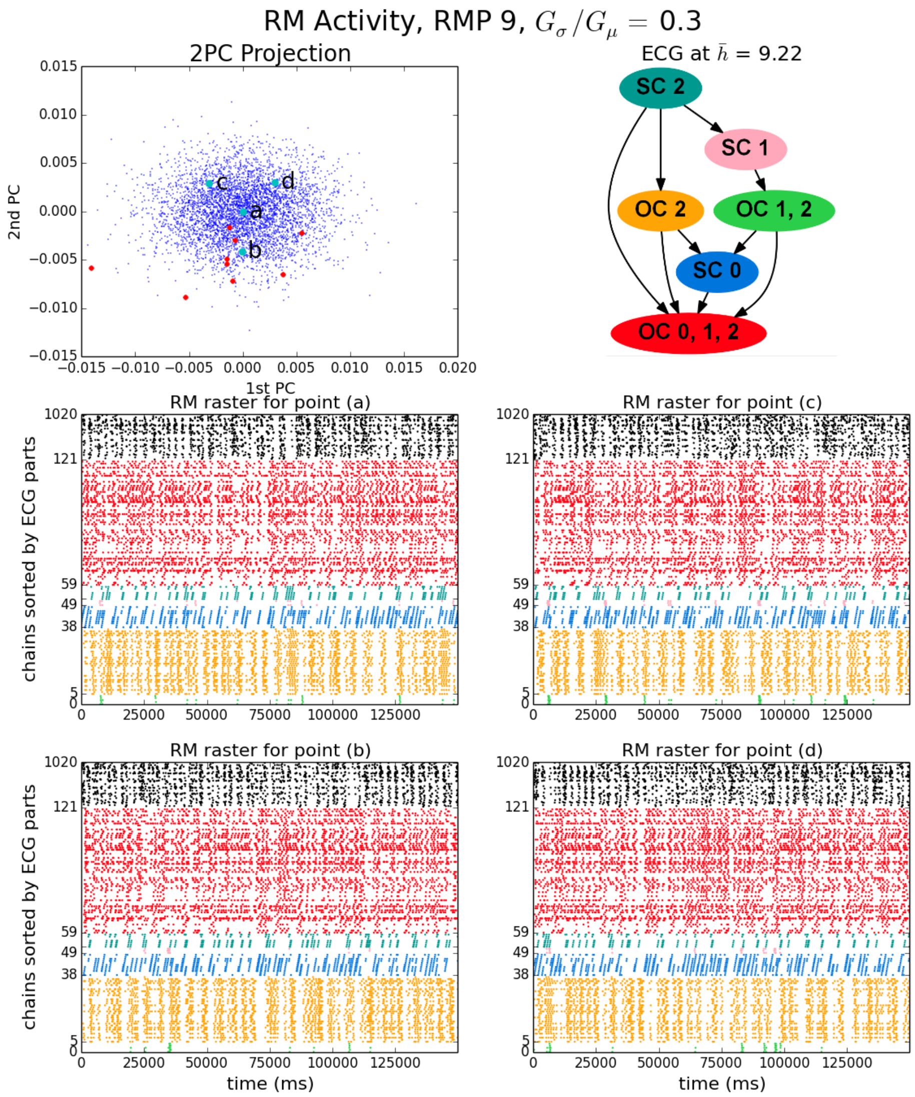

RM Activity, RMP 9, G_σ/G_μ = 0.3
2PC Projection
2nd PC
1st PC
c
d
a
b
ECG at $\bar{h}$ = 9.22
SC 2
SC 1
OC 2
OC 1, 2
SC 0
OC 0, 1, 2
RM raster for point (a)
RM raster for point (c)
RM raster for point (b)
RM raster for point (d)
chains sorted by ECG parts
time (ms)

Figure 17
